# Ruxolitinib plus steroids for acute graft versus host disease: a multicenter, randomized, phase 3 trial

**DOI:** 10.1038/s41392-024-01987-x

**Published:** 2024-10-23

**Authors:** Liping Dou, Yanli Zhao, Jingjing Yang, Lei Deng, Nan Wang, Xiawei Zhang, Qingyang Liu, Yan Yang, Zhijie Wei, Fuxu Wang, Yifan Jiao, Fei Li, Songhua Luan, Liangding Hu, Sujun Gao, Chuanfang Liu, Xiangjun Liu, Jinsong Yan, Xuejun Zhang, Fang Zhou, Peihua Lu, Daihong Liu

**Affiliations:** 1 State Key Laboratory of Experimental Hematology, Senior Department of Hematology, The Fifth Medical Center of PLA General Hospital, Beijing, China; 2 Department of Hematology, Hebei Yanda Lu Daopei Hospital, Langfang, China; 3 Department of Hematology, The 960th Hospital of The People’s Liberation Army (PLA) Joint Logistics Support Force, Jinan, China; 4 Department of Hematology, The Second Hospital of Dalian Medical University, Dalian, China; 5 Department of Hematology, The Second Hospital of Hebei Medical University, Shijiazhuang, China; 6 Department of Hematology, The First Hospital of Jilin University, Changchun, China; 7 Department of Hematology, Qilu Hospital, Shandong University, Jinan, China; 8 Beijing BFR Gene Diagnostics, Beijing, China

**Keywords:** Haematological diseases, Drug development

## Abstract

Newly diagnosed patients with high-risk acute graft-versus-host disease (aGVHD) often experience poor clinical outcomes and low complete remission rates. Ruxolitinib with corticosteroids showed promising efficacy in improving response and failure free survival in our phase I study. This study (ClinicalTrials.gov: NCT04061876) sought to evaluate the safety and effectiveness of combining ruxolitinib (RUX, 5 mg/day) with corticosteroids (1 mg/kg/day methylprednisolone, RUX/steroids combined group) versus using methylprednisolone alone (2 mg/kg/day, steroids-only group). Newly diagnosed patients with intermediate- or high-risk aGVHD were included, with risk levels classified by either the Minnesota aGVHD Risk Score or biomarker assessment. Patients were randomized in a ratio of 1:1 into 2 groups: 99 patients received RUX combined with methylprednisolone, while the other 99 received methylprednisolone alone as the initial treatment. The RUX/steroids group showed a significantly higher overall response rate (ORR) on day 28 (92.9%) compared to the steroids-only group (70.7%, Odds Ratio [OR] = 5.8; 95% Confidence Interval [CI], 2.4–14.0; *P* < 0.001). Similarly, the ORR on day 56 was higher in the RUX/steroids group (85.9% vs. 46.5%; OR = 7.07; 95% CI, 3.36–15.75; *P* < 0.001). Additionally, the 18-month failure-free survival was significantly better in the RUX/steroids group (57.2%) compared to the steroids-only group (33.3%; Hazard Ratio = 0.46; 95% CI, 0.31–0.68; *P* < 0.001). Adverse events (AEs) frequencies were comparable between both groups, with the exception of fewer grade 4 AEs in the RUX/steroids group (26.3% vs. 50.5% *P* = 0.005). To our knowledge, this study is the first prospective, randomized controlled trial to demonstrate that adding ruxolitinib to the standard methylprednisolone regimen provides an effective and safe first-line treatment for newly diagnosed high-risk acute GVHD.

## Introduction

The standard initial treatment for Acute graft-versus-host disease (aGVHD) has traditionally consisted of methylprednisolone at a dose of 2 mg/kg/day or prednisone at 2.0–2.5 mg/kg/day.^1–3^ The response rate to corticosteroids monotherapy is around 50%, and the 6-month overall survival rate for patients with intermediate- and high-risk aGVHD, as determined by the Minnesota aGVHD risk score and biomarker risk, is approximately 60%.^4–6^ There is currently a lack of new treatment strategies for intermediate- and high-risk aGVHD that can improve treatment effectiveness and patient outcomes. Current guidelines do not recommend combined first-line therapy. Previous studies have shown that using corticosteroids at a dose of 2 mg/kg/day with a second agent as first-line treatment for newly diagnosed aGVHD offered no significant clinical benefit compared to standard methylprednisolone treatment.^7–17^ Importantly, survival at 100 days was shorter with those escalated immunosuppression.^18^ Effectively controlling GVHD without subjecting aGVHD patients to more intense and prolonged immunosuppression remains a primary concern.

Ruxolitinib, a selective Janus kinase (JAK) 1/2 inhibitor, is approved for managing steroid-refractory aGVHD.^19–23^ Jan H reported that neutrophils infiltrating the ileum migrate to the mesenteric lymph nodes during the early phase of GVHD, contributing to its development.^24^ Ruxolitinib was shown to reduce neutrophil infiltration into the mesenteric lymph nodes and decrease MHC-II expression, thus mitigating an early event in the pathogenesis of acute GVHD. Additionally, enhanced JAK-STAT signaling has been identified as a factor responsible for the severe GVHD phenotype induced by MicroRNA-146a deficient dendritic cells.^25^ Early inhibition of JAK1/JAK2 in clinic setting has emerged as a potential therapeutic strategy, which may minimize GVHD evolution and decrease second line therapies. There are no previous prospective controlled studies reporting the efficacy of ruxolitinib for newly diagnosed aGVHD. Further research suggests that ruxolitinib could boost the effectiveness of corticosteroids in T cells.^23,26^ The combination appears to shift the balance of apoptotic factors, possibly overcoming corticosteroid resistance.^26^ Therefore, it is plausible to hypothesize that incorporating ruxolitinib could enhance corticosteroid efficacy as a first-line treatment while minimizing steroid exposure in patients with newly diagnosed aGVHD. The pharmacokinetics study on ruxolitinib demonstrated that inhibition of cytokine-induced pSTAT3 by ruxolitinib is dose-dependent with maximal inhibition occurring 1–2 h for all doses.^27,28^ The maximal mean inhibition of pSTAT3 was 40% at a dose of 5 mg and 54% at the dose of 10 mg, while 90% inhibition was achieved at the highest tolerable dose of 200 mg.^27^ This indicates that increasing the dose of ruxolitinib from 5 mg to 10 mg did not result in a doubling of the inhibitory effect on pSTAT3; the inhibitory at 5 mg was comparable to that at 10 mg. Therefore, it is reasonable to hypothesize that ruxolitinib at a 5 mg dose may be effectively utilized as part of first-line therapy for newly diagnosed aGVHD patients.

Accumulating evidence shows that ruxolitinib is associated with substantial myelosuppression activity, especially for the patients with aGVHD.^20–22^ The Food and Drug Administration’s approval of ruxolitinib for steroid-resistant aGVHD was based on REACH Study.^21^ In the REACH1 study,^21^ patients with steroid-resistant aGVHD were given an initial oral dose of ruxolitinib at 5 mg twice daily, with the option to increase to 10 mg twice daily after 3 days if there was no occurrence of cytopenia. However, in the REACH1 study, adverse events (AEs) led to ruxolitinib discontinuation in 32.4% of patients and dose reduction in 35.2% of patients.^21^ The median average daily dose of ruxolitinib was 10.3 mg/day (ranging from 5–20 mg/day), and the response on day 28 was not found to be associated with ruxolitinib doses in the REACH1 study.^21^ Similarly, in the REACH2 study,^20^ 37.6% of patients experienced AEs that led to dose modifications, and 16.4% had events that resulted in the discontinuation of ruxolitinib. In a study involving aGVHD patients with fibrosis, ruxolitinib at 5 mg/day was found to be effective without causing severe cytopenia, whereas 50% of patients treated with ruxolitinib 10 mg/day experienced severe cytopenia.^28^ In addition, prompt discontinuation or rapid tapering of ruxolitinib may lead to additional AEs such as aggressive, fatal GVHD.^20–23,26–28^ The patients’ haematopoietic reconstitutions are more fragile in the early stage after engraftment compared to those in later stages. As the timing is earlier for aGVHD patients who receive first-line therapy than for those who receive second-line therapy, the haematopoietic reconstitutions after the first onset of aGVHD are more susceptible to the hematologic toxicity of ruxolitinib. Therefore, it is reasonable to hypothesize that ruxolitinib at 5 mg/day can be effectively and safely utilized as a first-line treatment for newly diagnosed aGVHD patients.

We previously conducted a phase I dose-finding study to identify the optimal treatment for patients with newly diagnosed aGVHD, assessing various doses of ruxolitinib in combination with corticosteroids.^19^ This preliminary study suggested that initiating ruxolitinib at 5 mg/day, rather than 5 mg twice daily or 10 mg twice daily, could be both effective and safe. As a result, we designed the current phase 3 study to evaluate the efficacy and safety of a combined regimen of ruxolitinib (5 mg/day) and methylprednisolone (1 mg/kg/day) compared to a control regimen of methylprednisolone (2 mg/kg/day) in patients with newly diagnosed intermediate- or high-risk aGVHD.^29^

## Results

### Patients and baseline characteristics

**Fig. 1:**
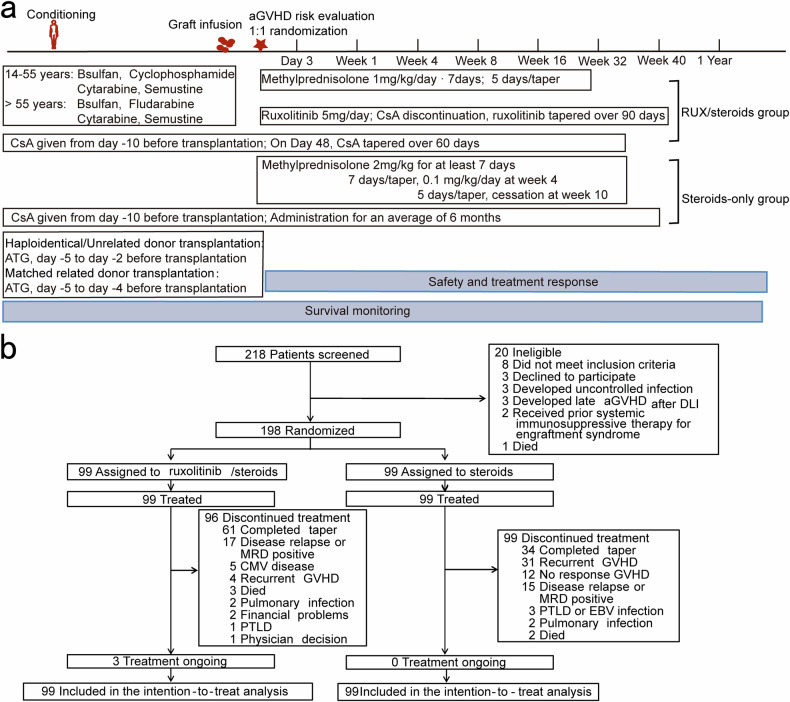
Study design and consort diagram. **a** Flow chart of treatment schedule. Newly diagnosed intermediate or high-risk acute graft-versus-host disease (aGVHD) patients underwent randomization and treatment of RUX/steroids combined or steroids only regimen after aGVHD risk evaluation. The medication of conditioning, GVHD prophylaxis regimen, and taper process of aGVHD treatment were described detail in “Methods”. Patients were monitored for safety, treatment response, and clinical endpoints. “Day”, “Week”, and “Year” referred to time after aGVHD treatment. **b** Enrollment of patients into the study. ATG anti-thymocyte globulin; CMV cytomegalovirus; CsA cyclosporine A; DLI donor lymphocyte infusion; EBV Epstein–Barr virus; GVHD graft versus host disease; MRD measurable residual disease; PTLD post-transplantation lymphoproliferative disorder; RUX ruxolitinib

From August 25, 2019, to June 1, 2022, a total of 198 patients with newly diagnosed intermediate- or high-risk aGVHD were enrolled from seven centers. These patients were randomized to the RUX/steroids combined group (n = 99) or the steroids-only group (n = 99) (Fig. 1 and Table 1). As of December 1, 2023, the median follow-up for surviving patients was 977.0 days (range, 553.0–1606.0 days) in the RUX/steroids combination group and 1052.0 days (range, 586.0–1600.0 days) in the steroids-only group. The baseline demographic, transplantation-related, and disease-related characteristics of the patients were comparable between the two groups (Table 1). Detailed biopsy data for acute GVHD target organs are provided in Supplemental data (Supplementary Table 1). All patients had neutrophil engraftment. There was no significant difference in the time to neutrophil engraftment between the two groups (median time: 12 days [interquartile range (IQR), 11–16] for the RUX/steroids combined group; 13 days [IQR, 11–15] for the steroids-only group; *P* = 0.889). Similarly, there was no significant difference in the time to platelet engraftment between the two groups (median time: 14 days [IQR, 11–19] for both groups; *P* = 0.897). In the RUX/steroids group, four patients did not complete the planned 56 days of therapy due to malignancy relapse/progression (n = 3) or death due to viral gastroenteritis (n = 1). Three patients in the steroids-only group did not complete the 56-day treatment due to malignancy relapse/progression (n = 1) or death from infection (n = 2). The distribution of aGVHD grades was similar between the two treatment groups (Table 1).

**Table 1:** Characteristics of the patients at baseline^a^

Characteristic	No. (%)	
	Ruxolitinib/steroids (*n* = 99)	Steroids only (*n* = 99)
Age, median (range), y	35.0 (14.0–65.0)	34.0 (14.0–64.0)
Male	65 (65.7)	67 (67.7)
Weight, median (range), kg	64.0 (42.8–98.1)	67.0 (42.2–118.0)
Body-mass index, median (range), kg/m^2^	22.5 (16.0–32.8)	23.3 (15.0–38.5)
Diagnosis of underlying malignant disease		
Acute myelogenous leukemia	54 (54.5)	40 (40.4)
Acute lymphoid leukemia	28 (28.3)	34 (34.3)
Myelodysplastic syndrome	5 (5.1)	11 (11.1)
Chronic myelogenous leukemia	1 (1.0)	2 (2.0)
Non-Hodgkin lymphoma	5 (5.1)	3 (3.0)
Other acute leukemia	2 (2.0)	5 (5.1)
Other leukemia	4 (4.0)	4 (4.0)
Disease Risk Index		
low	15 (15.2)	11 (11.1)
Intermediate	46 (46.5)	48 (48.5)
High	32 (32.3)	33 (33.3)
Very High	5 (5.1)	6 (6.1)
missing	1 (1.0)	1 (1.0)
HCT-CI		
0	59 (59.6)	47 (47.5)
1	22 (22.2)	40 (40.4)
2	9 (9.1)	5 (5.1)
3	5 (5.1)	3 (3.0)
4	2 (2.0)	3 (3.0)
5	1 (1.0)	0 (0.0)
missing	1 (1.0)	1 (1.0)
Time from diagnosis to HCT, median (range), d	169 (12–859)	212 (14–625)
Time from HCT to screening, median(range), d	25 (17–89)	25 (15–77)
Conditioning regimen		
Modified Bu/Cy	85 (85.9)	85 (85.9)
Bu/Flu	8 (8.1)	10 (10.1)
Modified TBI/Cy	6 (6.1)	4 (4.0)
Donor’s age, median (range), y	37 (14.0–63.0)	34 (8.0–57.0)
Donor-recipient ABO match		
Match	56 (56.6)	56 (56.6)
Major mismatch	21 (21.2)	19 (19.2)
Minor mismatch	17 (17.2)	18 (18.2)
Bidirectional mismatch	5 (5.1)	6 (6.1)
Donor-recipient gender match		
Female to male	24 (24.5)	19 (19.2)
Female to female	9 (9.1)	9 (9.1)
Male to female	26 (26.3)	23 (23.2)
Male to male	40 (40.4)	48 (48.5)
Graft		
MNCs, median (range), ×10^8^/kg	10.7 (2.8–28.0)	10.3 (2.3–32.7)
CD34^+^, median (range), ×10^6^/kg	4.9 (0.8–11.8)	4.9 (0.9–20.0)
Source of graft		
Matched sibling donor	12 (12.1)	10 (10.1)
Haploidentical donor	76 (76.8)	78 (78.8)
Unrelated donor	11 (11.1)	11 (11.1)
Cytomegalovirus positive at HCT	99 (100)	99 (100)
Donor cytomegalovirus positive at HCT	99 (100)	99 (100)
Overall aGVHD grade at baseline^b^		
Grade II	86 (86.9)	88 (88.9)
Grade III	10 (10.1)	6 (6.1)
Grade IV	3 (3.0)	5 (5.1)
aGVHD organ involvement at baseline		
Skin	63 (63.6)	72 (72.7)
Liver	6 (6.1)	7 (7.1)
Lower GI	24 (24.2)	30 (30.3)
Biomarker risk at baseline^b^		
Intermediate	55 (55.6)	55 (55.6)
High	44 (44.4)	44 (44.4)
Overall aGVHD risk		
Minnesota high and biomarker high	13 (13.1)	11 (11.1)
Minnesota standard and biomarker high	31 (31.3)	33 (33.3)
Minnesota standard and biomarker intermediate	55 (55.6)	55 (55.6)
Coadministrations of antifungal drugs		
Voriconazole	83 (83.8)	82 (82.8)
Posaconazole	8 (8.1)	7 (7.1)
Caspofungin	8 (8.1)	10 (10.1)

*aGVHD* acute graft versus host disease, *Bu* busulfan, *Cy* cyclophosphamide, *Flu* fludarabine, *GI* gastrointestinal tract, *HCT* hematopoietic stem cell transplant, *HCT-CI* hematopoietic cell transplant -comorbidity index, *MNC* mononuclear cells, *TBI* total body irradiation

^a^Data are presented as number (percentage) of patients unless otherwise indicated

^b^Baseline defined as the last acute GVHD assessment prior to or on randomization date +3 days, but no later than the treatment start date

### Primary endpoint

**Fig. 2:**
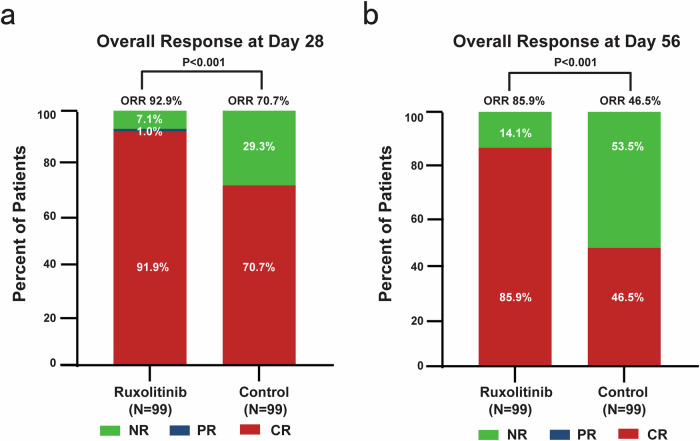
Overall response at day 28 and day 56. The primary endpoint was an overall response (complete or partial response) on day 28 (**a**), and the secondary endpoint was a overall response on day 56 (**b**). Two-sided *P* values were calculated using a stratified Cochran–Mantel–Haenszel test

The ORR on day 28 was significantly higher in the RUX/steroids combination group (92.9%) compared to the steroids-only group (70.7%, odds ratio [OR] = 5.8, 95% CI, 2.4–14.0; *P* < 0.001, Fig. 2a). For patients with grade III-IV aGVHD, the ORR on day 28 was 92.3% in the RUX/steroids combination group, compared to only 36.4% in the steroids-only group (Table 2). Among patients with grade II aGVHD, the ORR on day 28 was 93.0% in the RUX/steroids combination group, while it was 75.0% in the steroids-only group (OR = 4.4, 95% CI 1.7–11.6, *P* = 0.001, Table 2).

**Table 2:** Overall response rate at day 28 (Full analysis set)

	Ruxolitinib/steroidsn (%)	Steroids *n* (%)	Odds ratio (Ruxolitinib/ Steroids)	*P* value^a^
Intermediate and high-risk Number of patients	99	99		
ORR	92 (92.9)	70 (70.7)	5.8	<0.001
95% CI	86.0–97.1	60.7–79.4	2.4–14	
CR	91 (91.9)	70 (70.7)	4.79	<0.001
95% CI	84.7–96.4	60.7–79.4	2.06–11.12	
Intermediate-risk				
Number of patients	55	55		
ORR	51 (92.7)	45 (81.8)	2.8	0.086
95% CI	82.4–98.0	69.1–90.9	0.8–9.7	
High-risk				
Number of patients	44	44		
ORR	41 (93.2)	25 (56.8)	10.4	<0.001
95% CI	81.3–98.6	41.0–71.7	2.7–38.7	
Intermediate-biomarker-risk				
Number of patients	55	55		
ORR	51 (92.7)	45 (81.8)	2.8	0.086
95% CI	82.4–98.0	69.1–90.9	0.8–9.7	
High-biomarker-risk				
Number of patients	44	44		
ORR	41 (93.2)	25 (56.8)	10.4	<0.001
95% CI	81.3–98.6	41.0–71.7	2.7–38.7	
aGVHD grade				
Grade II				
Number of patients	86	88		
ORR	80 (93.0)	66 (75.0)	4.4	0.001
95% CI	85.4–97.4	64.6–83.6	1.7–11.6	
Grade III-IV				
Number of patients	13	11		
ORR	12 (92.3)	4 (36.4)	21.0	0.008
95% CI	64.0–99.8	10.9–69.2	1.9–227.205	
aGVHD risk by Minnesota				
aGVHD high risk				
Number of patients	13	11		
ORR	12 (92.3)	4 (36.4)	21.0	0.008
95% CI	64.0–99.8	10.9–69.2	1.9–227.205	
Patients’ age				
50 to 65 years				
Number of patients	24	20		
ORR	23 (95.8)	14 (70.0)	9.9	0.043
95% CI	78.9–99.9	45.7–88.1	1.1–90.7	
aGVHD organ involvement				
Skin				
Number of patients	63	72		
ORR	57 (90.5)	48 (66.7)	4.75	0.002
95% CI	80.4–96.4	54.6–77.3	1.79–12.57	
Liver				
Number of patients	6	7		
ORR	5 (83.3)	5 (71.4)	2.00	0.615
95% CI	35.9–99.6	29.0–96.3	0.13–29.81	
Lower GI				
Number of patients	24	30		
ORR	24 (100)	17 (56.7)	2.403975e + 08	<0.001
95% CI	85.8–100	37.4–74.5	2-inf	

*aGVHD* acute graft versus host disease, *ORR* overall response rate, *CR* complete remission, *CI* confidence interval, *GI* gastrointestinal

^a^*P* values are two-sided and unadjusted for multiplicity of analyses

Among patients with high-risk aGVHD based on biomarker measurement, the ORR on day 28 was 93.2% in the RUX/steroids combination group compared to 56.8% in the steroids-only group (OR = 10.4, 95% CI 2.7–38.7; *P* < 0.001, Table 2). Among patients with intermediate-risk aGVHD based on biomarker measurements, the corresponding ORRs on day 28 were 92.7% vs. 81.8% (OR = 2.8, 95% CI, 0.8–9.7; *P* = 0.086, Table 2).

The ORR on day 28 was significantly higher in the RUX/steroids combined group, regardless of the organs involved: 100.0% for RUX/steroids vs 56.7% for steroids (lower gastrointestinal disease, *P* < 0.001); 90.5% for RUX/steroids vs 66.7% for steroids (skin disease, *P* = 0.002); 95.7% for RUX/steroids vs 76.3% for steroids (upper gastrointestinal disease, *P* = 0.017); 83.3% for RUX/steroids vs 71.4% for steroids (liver disease, *P* = 0.615, Table 2).

### Secondary endpoints

**Fig. 3:**
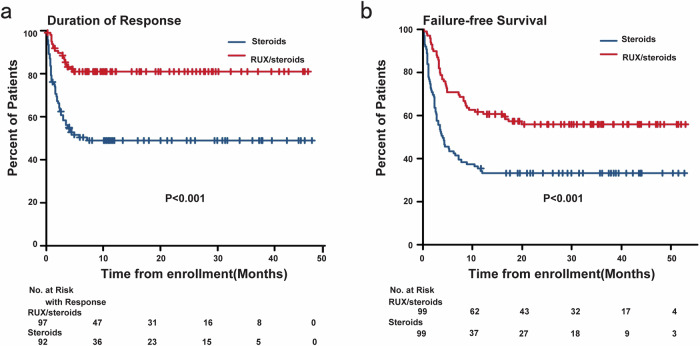
Duration of response (**a**) and failure-free survival (**b**)

The ORR on day 56 was notably higher in the RUX/steroids combination group compared to the steroids-only group (85.9% vs. 46.5%; OR = 7.07; 95% CI, 3.36–15.75; *P* < 0.001, Fig. 2b). For patients who showed a response at any time, the 6-month probability of DOR was 81.3% (95% CI, 72.9–88.4%) in the RUX/steroids group, compared to 51.1% (95% CI, 41.4–61.6%) in the steroids-only group (*P* < 0.001; Fig. 3a). In the RUX/steroids combination group, 25 patients (25.3%) required second-line therapies for recurrent aGVHD, compared to 54 patients (54.5%) in the steroids-only group (*P* < 0.001; Supplementary Table 2).

The cumulative incidence of chronic GVHD of any grade at 18 months was 19.1% (95% CI, 16.6–29.4%) in the RUX/steroids combined group and 26.2% (95% CI, 19.7–34.1%) in the steroids-only group (*P* = 0.377). Furthermore, 78.9% of patients in the RUX/steroids group and 70.1% in the steroids-only group were off immunosuppressant therapy 18 months post-transplantation (*P* = 0.487).

The 18-month failure-free survival was significantly higher in the RUX/steroids combination group compared to the steroids-only group (RUX/steroids 57.2%, steroids-only 33.3%; HR 0.46 [95%CI, 0.31–0.68]; *P* < 0.001; Fig. 3b; Supplementary Fig. 1). Both groups had comparable 18 months cumulative incidence rates of relapse (RUX/steroids 17.4% [95% CI, 10.7–25.6%], steroids-only 17.2% [95% CI, 10.5–25.3%]; *P* = 0.679; Supplementary Fig. 2) and non-relapse mortality (NRM) (RUX/steroids 12.3% [95% CI, 6.7–19.6%], steroids-only 16.2% [95% CI, 9.8–24.2%]; *P* = 0.513; Supplementary Fig. 3). The 18-month OS rates were similar between the groups, with 75.4% in the RUX/steroids group and 70.5% in the steroids-only group (HR 0.91 [95% CI, 0.54–1.54]; *P* = 0.734; Supplementary Fig. 4), as were the 18-month DFS rates (RUX/steroids 71.4%, steroids-only 67.4%; HR 0.91 [95% CI, 0.55–1.50]; *P* = 0.720; Supplementary Fig. 5, Supplementary Table 3).

For patients with grade III-IV aGVHD, the 18-month NRM tended to be lower in the RUX/steroids combination group compared to the steroids-only group (16.3% vs 45.5%; HR 0.29 [95% CI, 0.06–1.52]; *P* = 0.088; Supplementary Fig. 6). Among patients with high risk aGVHD, the 18-month DFS tended to be higher in the RUX/steroids group than in the steroids-only group (RUX/steroids 70.5%, steroids-only 61.3%; HR 0.74 [95%CI, 0.36–1.53]; *P* = 0.417).

The steroids-only group received a significantly higher cumulative dose of methylprednisolone (27.8 ± 7.6 mg/kg) compared to the RUX/steroids combination group (17.6 ± 3.6 mg/kg), and this difference was statistically significant (*P* < 0.001). Additionally, the duration of methylprednisolone treatment was significantly longer in the steroids-only group compared to the RUX/steroids combination group (48.7 ± 24.4 days vs. 29.4 ± 9.3 days; *P* = 0.032).

### Safety and tolerance analysis

The median duration of ruxolitinib exposure was 177.0 days (range, 17.0–447.0 days) in 96 patients, and this duration varied due to several reasons: disease relapse or measurable residual disease (n = 17), CMV disease (n = 5), post-transplantation lymphoproliferative disease (n = 1), pulmonary infection (n = 2), financial problems (n = 2), physician decision (n = 1), recurrent GVHD (n = 4), death (n = 3), or scheduled design (n = 61). None of the 96 patients discontinued ruxolitinib due to hepatotoxicity.

The most frequently observed AEs up to day 28 post-treatment were anemia, gamma-glutamyl transferase increase, and hyperglycemia (Table 3). Grade 3 or higher AEs occurred in 87.9% of patients in the RUX/steroids group and 84.8% in the steroids-only group (*P* = 0.534). Grade 4 AEs were more common in the steroids-only group, affecting 50.5% of patients, compared to 26.3% in the RUX/steroids group (*P* = 0.005). Thrombocytopenia, a known side effect of ruxolitinib, was also prevalent, with 24.2% of patients in the RUX/steroids group and 41.4% in the steroids-only group experiencing grade 4 thrombocytopenia. Importantly, all cases of thrombocytopenia were reversible, and none of the 96 patients discontinued ruxolitinib due to decreased platelet count. The percentages of patients who experienced fungal infections, bacterial infections, CMV reactivation at 6 months, Epstein–Barr virus reactivation and post-transplantation lymphoproliferative disease occurred at similar rates between the RUX/steroids and steroids-only groups (all *P* > 0.05; Supplementary Tables 4–5). Assessment of control of hyper glycemia and hypertension, degree of quality-of-life (assessed by MD Anderson Cancer Center Symptom inventory, MDASI), blood potassium and calcium concentrations during the first 45 days of treatment showed no statistically significant differences between the two groups, although there were trends that patients in RUX/steroids group had lower blood glucose concentrations and more reduction in MDASI severity (Supplementary Fig. 7).

**Table 3:** Most frequent adverse events up to day 28 (safety population)

Characteristic	Ruxolitinib/steroids (n = 99)				Steroids (n = 99)			
	Grade 1-2	Grade 3	Grade 4	Grade 5	Grade 1-2	Grade 3	Grade 4	Grade 5
Any event	12 (12.1)	61 (61.6)	26(26.3)	0	15 (15.2)	34 (34.3)	50 (50.5)	0
Anemia	50(50.5)	46 (46.5)	0	0	60 (60.6)	32 (32.3)	6 (6.1)	0
Platelet count decreased	26 (26.3)	34 (34.3)	24 (24.2)	0	19 (19.2)	28 (28.3)	41 (41.4)	0
Neutropenia	46 (46.5)	19 (19.2)	5 (5.1)	0	17 (17.2)	17 (17.2)	17 (17.2)	0
Leukopenia	50 (50.5)	17 (17.2)	5 (5.1)	0	21 (21.2)	11 (11.1)	19 (19.2)	0
Diarrhea	12 (12.1)	0	0	0	17 (17.2)	2 (2.0)	2 (2.0)	0
Pyrexia	26 (26.3)	0	0	0	24 (24.2)	2 (2.0)	0	0
Febrile neutropenia	0	0	0	0	0	2 (2.0)	0	0
Cytomegalovirus infection	58 (58.6)	9 (9.1)	0	0	52 (52.5)	9 (9.1)	0	0
Epstein-Barr virus infection	65 (65.7)	5 (5.1)	0	0	67 (67.7)	4 (4.0)	0	0
Hypertension	9 (9.1)	5 (5.1)	0	0	17 (17.2)	9 (9.1)	0	0
Hypokalemia	48 (48.5)	31 (31.3)	0	0	47 (47.5)	19 (19.2)	4 (4.0)	0
Alanine aminotransferase increased	50 (50.5)	5 (5.1)	0	0	54 (54.5)	13 (13.1)	0	0
Aspartate aminotransferase increased	36 (36.4)	5 (5.1)	0	0	50(50.5)	4 (4.0)	0	0
Gamma-glutamyltransferase increased	60 (60.6)	29 (29.3)	2 (2.0)	0	60 (60.6)	28 (28.3)	4 (4.0)	0
Fatigue	22 (22.2)	2 (2.0)	0	0	24 (24.2)	0	0	0
Abdominal pain	0	0	0	0	2 (2.0)	0	0	0
Back pain	4 (4.0)	0	0	0	0	0	0	0
Hypomagnesaemia	50 (50.5)	0	0	0	43 (43.4)	2 (2.0)	0	0
Hyperglycemia	89 (89.9)	0	0	0	82 (82.8)	11 (11.1)	0	0
Hypocalcemia	41 (41.4)	0	0	0	41(41.4)	2 (2.0)	0	0
Hyperkalemia	2 (2.0)	0	0	0	0	0	0	0
Blood creatinine increased	7 (7.1)	0	0	0	9 (9.1)	0	0	0
Acute kidney injury	0	0	0	0	0	0	0	0
Urinary tract infection	7 (7.1)	0	0	0	9 (9.1)	0	0	0
hemorrhagic cystitis	19 (19.2)	7 (7.1)	0	0	15(15.2)	9 (9.1)	0	0
Nausea	24 (24.2)	0	0	0	11 (11.1)	0	0	0
Vomiting	12(12.1)	0	0	0	2 (2.0)	0	0	0
Hypophosphatemia	29 (29.3)	0	0	0	37 (37.4)	0	0	0
Fall	5 (5.1)	0	0	0	2 (2.0)	0	0	0
Pneumonia	7 (7.1)	5 (5.1)	0	0	9 (9.1)	4 (4.0)	0	0
Hypoalbuminaemia	65 (65.7)	0	0	0	75 (75.8)	2(2.0)	0	0
Peripheral edema	0	0	0	0	4 (4.0)	0	0	0
Sepsis	0	0	0	0	0	0	0	0

According to Common Terminology Criteria for Adverse Events (CTCAE) Version 5.0 Published: November 27, 2017

### Changes of GVHD biomarkers and immune reconstitution

We evaluated the changes in biomarker levels in aGVHD patients treated with different first-line therapies (RUX/steroids and steroids only). There were no differences in pre-enrollment biomarker scores based on sST2 and REG3α between CR and refractory patients in either group. For the patients who achieved complete remission on day 14 and 28 after RUX/steroids combined treatment, levels of REG3α and tumor necrosis factor receptor 1 (TNFR1) significantly decreased by day 14 compared to their pre-enrollment levels (REG3α: *P*_14_ = 0.002 and *P*.adjust=0.005, *P*_28_ = 0.583 and *P*.adjust=1.000; TNFR1: *P*_*14*_ = 0.002 and *P*.adjust = 0.005, *P*_*28*_ = 0.057 and *P*.adjust = 0.172; Supplementary Fig. 8a). Meanwhile, levels of other biomarkers after RUX/steroids combined treatment did not differ significantly between groups (Supplementary Fig. 8).

In this study, the CI of CD4^+^ T cell reconstitution (CD4^+^ cell count ≥0.05 × 10^9^/L at two consecutive measurements within 100 days post-transplantation ^30,31^) was also assessed. Patients treated with RUX/steroids appeared to have a higher CI of CD4^+^ T cell immune reconstitution by day 100, compared with patients treated with steroids only (RUX/steroids 65.7% [95% CI, 54.4–76.7%], steroids-only 56.0% [95% CI, 45.2–67.4%]; *P* = 0.231; Supplementary Fig. 9a). On 28 days, 180 days and 365 days after enrollment, patients after RUX/steroids combined treatment had higher level of Treg cells compared with patients after steroids only treatment (*P*_*28*_ = 0.007, *P*_*180*_ = 0.059 and *P*_*365*_ = 0.042, Supplementary Fig. 9b, g, h). On 180 days after enrollment, patients receiving RUX/steroids combined treatment had more CD3^+^ T (*P* = 0.019), CD8^+^ T (*P* = 0.025) and NK cells (*P* = 0.030) compared with patients receiving steroids only treatment (Supplementary Fig. 9c, d, f).

## Discussion

This is the first prospective, randomized controlled trial to show that combining ruxolitinib with methylprednisolone is a superior first-line therapy for intermediate- and high-risk aGVHD compared to the standard 2 mg/kg/day methylprednisolone regimen. This novel approach resulted in significantly improved ORRs on days 28 and 56, more durable response at 6 months, and better failure-free survival compared to corticosteroid monotherapy. Responses were observed regardless of the involved organs and the patients’ risk status based on biomarkers. Moreover, the novel first-line therapy was associated with reduced exposure to steroids and was well-tolerated.

The significant improvement of durable response with the ruxolitinib/steroids combination regimen as a first-line therapy is important from several aspects. An important aspect of this novel regimen is the use of methylprednisolone at 1 mg/kg/day instead of traditional 2 mg/kg/day. This adjustment effectively controls GVHD without exposing aGVHD patients to more intense and prolonged immunosuppression. In earlier trials, a 2 mg/kg/day dose of methylprednisolone was commonly used when combined with a secondary agent as first-line treatment for newly diagnosed aGVHD.^10,14,16^ Mielcarek et al. reported that in patients with grade IIb or higher aGVHD, initial treatment with 1 mg/kg methylprednisolone was linked to an increased need for secondary immunosuppressive therapy but did not negatively impact survival.^30^ In our novel regimen, the initial lower dose of 1 mg/kg/day was selected to complement the addition of ruxolitinib to the standard first-line therapy, with the goal of minimizing the risk of refractory aGVHD.

Another important aspect of this novel regimen is that 5 mg of ruxolitinib was administered once daily. Ruxolitinib is primarily metabolized by the cytochrome P450 (CYP450) enzymes, specifically CYP2C9 and CYP3A4.^31,32^ Azoles are potent CYP3A4 inhibitors and commonly used for prophylaxis or treatment of fungal infections during aGVHD treatment. Without the concomitant use of azoles, the plasma half-life of ruxolitinib is approximately 3 h, and its accumulation following repeated dosing is insignificant, and the maximal mean inhibitory effect of twice daily dosing is close to that of once-daily dosing.^31–33^ A previous study reported that the elimination half-life of ruxolitinib increases by approximately 2.5-fold when administered in combination with fluconazole.^31^ Our previous study also demonstrated that co-administration of ruxolitinib with voriconazole doubled ruxolitinib exposure.^32^ In this study, most patients used azoles concurrently with ruxolitinib, leading to increased exposure. As a result, the ruxolitinib dose was reduced to 5 mg/day. Therefore, the use of ruxolitinib at 5 mg once daily as part of first-line therapy for aGVHD in our study is well-justified.

While improved survival would offer strong support for the efficacy of a treatment in GVHD trials, it is important to note that successful control of GVHD does not always correlate with enhanced survival. For instance, Levine JE et al. found that although there were significant differences in day 28 response rates between etanercept plus steroids and steroids alone for treating acute GVHD, survival differences were only seen in patients with related donors, but not those with unrelated donors.^11^ Among related donors, the gap in survival between treatment groups was much smaller than the difference in response rates.^11^ Similarly, Robert Zeiser reported a higher overall response rate on day 28 in the ruxolitinib group compared to the control group (62% vs. 39%; *P* < 0.001) in patients with glucocorticoid-refractory acute GVHD, but no significant difference in 18-month overall survival (37.69% in the ruxolitinib group vs. 36.18% in the control group).^20^ In GVHD treatment trials, discrepancies between response and survival outcomes are likely influenced by factors such as infections, regimen-related toxicity, relapse of malignancy, and underlying conditions unrelated to GVHD.

The difference in ORR between the RUX/steroids and steroids-only group was bigger at day 56 compared to day 28. The reason may be due to two points. Firstly, the early administration of ruxolitinib may lead to bigger difference in ORR at day 56 compared to day 28. Early ruxolitinib as first-line therapeutic strategy may minimize GVHD evolution and decrease second line therapies, which supported the efficacy of ruxolitinib for steroid-refractory disease. Secondly, the continuous administration of ruxolitinib from day 28 to day 56 after enrollment in the RUX/steroids combined group may increase the difference in ORR between the two groups. In the RUX/steroids combined group, methylprednisolone was discontinued by day 42 after enrollment, CsA was discontinued by day 102 and ruxolitinib by approximately day 180. Thus, on day 56, patients in the RUX/steroids group were treated with ruxolitinib and CsA. In the steroids-only group, patients received CsA and 0.1 mg/kg/day methylprednisolone by day 56. Thereby, the difference in ORR may be attributed to the early and sustained administration of ruxolitinib. Ruxolitinib treatment in recipients of allogeneic stem cell transplants has been shown to increase regulatory T cells, which are associated with immunologic tolerance.^34,35^ To maximize the potential benefits of ruxolitinib, calcineurin inhibitors were discontinued earlier than ruxolitinib after RUX/steroids treatment. A short duration of calcineurin inhibitor treatment and the absence of recurrent aGVHD may accelerate immune reconstitution. Monitoring immune reconstitution of lymphocyte subgroups reveals a superior recovery of CD4^+^ T cells and Treg cells. These findings emphasize the advantage of the RUX/steroids strategy in promoting immune reconstitution.

This carefully designed ruxolitinib/steroids combination regimen showed encouraging safety results. The main concerns when adding ruxolitinib to methylprednisolone are the potential for increased myelosuppression and thrombocytopenia.^20,36,37^ Rates of cytopenia, both of any grade and grade 3 or higher, were similar between the two groups. However, a major concern with ruxolitinib use is the increased risk of viral infections.^21,37,38^ Our study demonstrated that adding ruxolitinib at a dose of 5 mg/day did not increase the CMV and EBV reactivation rates.

The present trial has some limitations. This study was not blinded. The reason was that the tapering strategies for CsA and methylprednisolone differed between the two groups. The mean age of the enrolled patients was relatively young, at 35.8 years, which might have led to an overestimation of the activity of the ruxolitinib/corticosteroids combined regimen. However, it is worth noting that the ORR on day 28 in patients aged 50–65 years was 95.8% in the RUX/steroids group, compared to 70.0% in the steroids-only group. The high proportion of patients with grade II aGVHD in this study may have contributed to the high response rate, although this proportion is similar to those reported in other studies.^11,14,18^ Acute GVHD in our study was primarily diagnosed based on clinical findings, which could have influenced the therapeutic evaluation of ruxolitinib. Response with ruxolitinib was not improved largely as compared with steroids for patients with aGVHD involving the liver in this study.

In conclusion, the first-line use of 1 mg/kg methylprednisolone combined with 5 mg/day ruxolitinib was superior to the conventional therapy of 2 mg/kg/day methylprednisolone for aGVHD, as demonstrated by significantly improved durable overall response and failure-free survival. This enhancement in first-line aGVHD treatment was achieved without increasing steroid exposure, non-relapse mortality, or infectious complications.

## Materials and methods

### Study design

The study protocol has been previously published^29^ and registered in ClinicalTrials.gov (Identifier: NCT04061876). This was an open-label, multicenter, randomized phase 3 controlled trial that enrolled patients with newly diagnosed aGVHD who required initial systemic immunosuppressive therapy. The enrollment started on August 25, 2019, and was completed on June 1, 2022, involving seven Chinese transplantation centers. Patients were randomly 1:1 to receive either ruxolitinib (5 mg/day) plus methylprednisolone (1 mg/kg/day) or methylprednisolone (2 mg/kg/day) alone. The randomization was stratified by pre-transplant disease status (complete remission vs. not) and aGVHD risk (intermediate vs. high). Intermediate aGVHD risk was defined by the low risk of Minnesota aGVHD Risk Score^4^ and intermediate biomarker risk. High aGVHD risk was defined by the high risk of Minnesota aGVHD Risk Score^4^ or high biomarker risk. The diagnosis and response are clinically based and not histology based. Stratified permuted block randomization lists ensured balanced stratification. Randomization was performed through an independent interactive web-based response system, with codes generated by a statistician not involved in the study or site operations. This study was open-label, and both the investigators and participants were aware of the treatment groups due to differences in the tapering strategies for CsA and methylprednisolone. However, the study staff responsible for data analysis and outcome assessments were blinded to the treatment allocations.^29^

### Endpoints

The primary endpoint was the overall response rate (ORR) to aGVHD treatment at 28 days post-enrollment.^39^ ORR was defined as the percentage of patients in each group who attained either a partial response (PR) or complete response (CR) without requiring additional immunosuppressive agents. In aGVHD, CR was characterized by the complete absence of aGVHD symptoms, while PR was defined as an improvement of at least one stage in a single organ without worsening in others. No response (NR) was classified as either no improvement, worsening symptoms in any organ, or the emergence of new GVHD-related symptoms. Additionally, GVHD progression after 3 days of therapy or lack of improvement within 7 days was also considered NR.

Secondary endpoints included ORR on day 56 and the duration of response (DOR) at 6 months, non-relapse mortality (NRM), incidence of relapse, recurrent aGVHD, incidence of chronic GVHD, overall survival (OS), disease-free survival (DFS), and failure-free survival (FFS, detail information in Supplemental Methods). Secondary systemic therapy for aGVHD was assessed within 6 months after enrollment. The dose and duration of corticosteroids used were analyzed. The safety endpoints were assessed by the frequency of AEs, which were as defined according to the National Cancer Institute Common Terminology Criteria for Adverse Events (CTCAE, version 4.0, Supplemental Methods).

### Patients and eligibility

Inclusion criteria included: (1) age between 14 and 65 years; (2) newly diagnosed aGVHD; and (3) intermediate- or high-risk aGVHD, as determined by the Minnesota aGVHD Risk Score^4^ (high risk) or biomarker risk classification (intermediate- or high-risk).^5,28,29,40^ Exclusion criteria comprised: (1) chronic GVHD; (2) late aGVHD after donor lymphocyte infusion; (3) prior systemic immunosuppressive therapy for aGVHD; (4) contraindications to methylprednisolone; or (5) treatment with JAK inhibitor therapy after graft infusion. The study was approved by the Ethics Committee of the Chinese People’s Liberation Army General Hospital, and all participating patients provided informed consent prior to enrollment.

### Intervention

#### GVHD prophylaxis and supportive therapy

The enrolled patients had been diagnosed with hematologic malignancies and had undergone allogeneic peripheral blood stem cell transplantation. The procedures for hematopoietic stem cell mobilization, collection, conditioning regimens, and GVHD prophylaxis were conducted as previously described^19,41–43^ Most transplantation recipients received rabbit anti-thymocyte globulin (rATG), cyclosporine A (CsA), mycophenolate mofetil, and short-term methotrexate for GVHD prophylaxis (detail information in Supplemental Methods). Quantification of EBV and CMV DNA was performed by polymerase chain reaction (PCR) analysis twice weekly until 3 months after transplantation. Supportive care was provided as previously described.^19^

### GVHD treatment

Blood samples were collected prior to the initiation of corticosteroid therapy to assess biomarker status, with results expected within 24 h after starting corticosteroids. Ruxolitinib therapy was administered within 24 h after corticosteroid treatment, based on biomarker risk. Ruxolitinib treatment was not initiated until biomarker results were available. Patients identified with low biomarker-based risk were excluded, and the treatment regimen was determined by the physician.

#### Ruxolitinib plus corticosteroids treatment

In the RUX/steroids combined group, patients initially received intravenous methylprednisolone (Pfizer, NY, USA) at a dosage of 1 mg/kg/day for at least 7 days. Ruxolitinib (Jakavi, Novartis, Nurnberg, Germany) was administered orally at a daily dose of 5 mg. If GVHD patients responded to treatment, achieving either PR or CR at 7 days, the dosage of methylprednisolone was gradually reduced. A recommended taper schedule was provided to discontinue methylprednisolone by 6 weeks (Supplemental Methods). Following the discontinuation of steroid therapy and no recurrence of GVHD, CsA was tapered over a 60-day period. Following the discontinuation of CsA, if there was no recurrence of GVHD, ruxolitinib was tapered over 90 days, with a total duration of approximately 6 months.

#### Corticosteroids treatment

In the steroids-only group, patients were initially administered methylprednisolone at a dose of 2 mg/kg/day, divided into two doses daily, for at least 7 days before starting dose reduction. The methylprednisolone dosage was gradually reduced after achieving CR and tapered over 10 weeks (Supplemental Methods). CsA was administered intravenously at 2 mg/kg twice daily, aiming for target trough levels of 150–250 ng/mL. In the steroids-only group, CsA treatment lasted for about 6 months.

#### Second-line therapy

In both groups, second-line therapy was initiated for patients with refractory aGVHD, which was defined as GVHD progression after 3 days of therapy, lack of improvement within 7 days, or failure to achieve CR after 14 days of treatment (see supplemental Methods and Supplementary Table 2 for more details).

### Luminex assays for aGVHD biomarker measurement

An algorithm score based on ST2 and REG3α concentrations at the onset of GVHD was used to classify aGVHD patients into high-, medium-, and low-risk groups. Blood samples were collected for the measurement of aGVHD biomarkers at various time points, including before patients underwent the conditioning regimen, on days 7, 14, 28, 60, and 90 after transplantation, at the onset of aGVHD, and 3–7 days after enrollment (Supplemental Methods).

### Immunophenotyping

Immune monitoring was conducted on peripheral blood samples collected on 28, 56, 180, 365, 560 and 720 days after enrollment. Antibodies against CD3, CD4, CD8, CD20 and CD56 (BD Biosciences, USA) were used to detect CD3^+^ T, CD4^+^ T, CD8^+^ T, B and NK cells via BD FACS Canto II (BD Biosciences, USA). Treg cells were identified by gating the population of CD3^+^CD4^+^CD25^+^Foxp3^+^ cells.

### Sample size

The sample size calculation was based on the primary endpoint (ORR). In our previously phase I study involving 32 aGVHD patients treated with corticosteroids as first-line therapy, the expected ORR was 55%.^19^ Additionally, an ORR of 82.05% was observed in patients with aGVHD grades I–IV who received a combination of steroid and ruxolitinib as first-line therapy, including twelve patients with grade I aGVHD. Therefore, an expected ORR of 75% was established for those treated with the steroid-ruxolitinib combination. The study was designed with a 2-sided significance level α = 5% and a power of 1-β = 80%. 86 patients were required for each group as estimated using Z-Test statistics of PASS software (NCSS LCC, USA). Allowing a drop-out rate of 15%, a total of 198 patients were required (99 for each group, Fig. 1).^29^

### Statistical analysis

All randomized participants will be analyzed for outcomes, with missing data expected to be under 10%. If needed, multiple imputations will be applied, and best-case/worst-case scenarios used for handling missing data.

Continuous data are presented as median with interquartile range (IQR) or mean and standard deviation (SD), depending on the normality of the distribution. Categorical data are described as n (%). The ORR, along with its 95% CI, was assessed using the Cochran–Mantel–Haenszel test, stratified by pre-transplant disease status and biomarker risk. The Kaplan–Meier method estimated DOR, OS, DFS, and FFS, with group differences analyzed by the log-rank test. Cumulative incidence rates of recurrent aGVHD, cGVHD, NRM, and relapse were estimated using a competing risk model and compared with the Fine and Gray test. Competing events were defined as follows: for recurrent aGVHD, relapse and death without recurrent aGVHD; for cGVHD, relapse and death without cGVHD; for relapse, death without relapse; and for NRM, relapse. For biomarker and immune reconstitution analyses, the Friedman test was used to identify statistically significant differences among time points for each biomarker. If the Friedman test was significant, Wilcoxon tests were conducted as follow-up tests, with Bonferroni correction applied to adjust the p-value for each biomarker analysis. A two-sided *P* < 0.05 was considered statistically significant. All analyses were performed using SPSS 22.0 software (IBM Corporation, Armonk, NY, USA) or R version 4.1.2 (www.cran.r-project.org), and all statistical analyses were based on the intend-to-treat set.

## Supplementary information

Supplementary Materials

Study Protocol

## Data Availability

De-identified individual participant data underlying the results reported in this article will be available beginning 9 months after publication and ending 24 months post-publication. Data will be provided to investigators whose proposed use of the data has been approved. Data requests should be directed to the corresponding author (daihongrm@163.com) and will be evaluated by an independent review committee on a case-by-case basis. After 24 months, participant data will be available upon request to the corresponding author and, after de-identification, following the moderated access approach of the data repository unit at Chinese PLA General Hospital, Beijing, China.

## References

[1] Socié, G., Kean, L. S., Zeiser, R. & Blazar, B. R. Insights from integrating clinical and preclinical studies advance understanding of graft-versus-host disease. *J. Clin. Investig.* **131**, e149296 (2021). 34101618 10.1172/JCI149296 PMC8203454

[2] Moreno, D. F. & Cid, J. Graft-versus-host disease. *Med Clin.* **152**, 22–28 (2019). 10.1016/j.medcli.2018.07.012 30309668

[3] Aladağ, E., Kelkitli, E. & Göker, H. Acute graft-versus-host disease: a brief review. *Turk. J. Haematol.* **37**, 1–4 (2020). 31475512 10.4274/tjh.galenos.2019.2019.0157 PMC7057746

[4] MacMillan, M. L. et al. Validation of Minnesota acute graft-versus-host disease risk score. *Haematologica* **105**, 519–524 (2020). 31320554 10.3324/haematol.2019.220970 PMC7012472

[5] Levine, J. E. et al. A prognostic score for acute graft-versus-host disease based on biomarkers: a multicentre study. *Lancet Haematol.* **2**, e21–e29 (2015). 26687425 10.1016/S2352-3026(14)00035-0 PMC4340092

[6] Penack, O. et al. Prophylaxis and management of graft-versus-host disease after stem-cell transplantation for haematological malignancies: updated consensus recommendations of the European Society for Blood and Marrow Transplantation. *Lancet Haematol.* **11**, e147–e159 (2024). 38184001 10.1016/S2352-3026(23)00342-3

[7] Jamy, O., Zeiser, R. & Chen, Y. B. Novel developments in the prophylaxis and treatment of acute GVHD. *Blood* **142**, 1037–1046 (2023). 37471585 10.1182/blood.2023020073

[8] Kebriaei, P. et al. Adult human mesenchymal stem cells added to corticosteroid therapy for the treatment of acute graft-versus-host disease. *Biol. Blood Marrow Transplant.* **15**, 804–811 (2009). 19539211 10.1016/j.bbmt.2008.03.012

[9] Bolaños-Meade, J. et al. Phase 3 clinical trial of steroids/mycophenolate mofetil vs steroids/placebo as therapy for acute GVHD: BMT CTN 0802. *Blood* **124**, 3221–3227 (2014). quiz 3335. 25170121 10.1182/blood-2014-06-577023 PMC4239331

[10] Alousi, A. M. et al. Etanercept, mycophenolate, denileukin, or pentostatin plus corticosteroids for acute graft-versus-host disease: a randomized phase 2 trial from the blood and marrow transplant clinical trials network. *Blood* **114**, 511–517 (2009). 19443659 10.1182/blood-2009-03-212290 PMC2713466

[11] Levine, J. E. et al. Etanercept plus methylprednisolone as initial therapy for acute graft-versus-host disease. *Blood* **111**, 2470–2475 (2008). 18042798 10.1182/blood-2007-09-112987 PMC2361693

[12] Couriel, D. R. et al. A phase III study of infliximab and corticosteroids for the initial treatment of acute graft-versus-host disease. *Biol. Blood Marrow Transplant.* **15**, 1555–1562 (2009). 19896079 10.1016/j.bbmt.2009.08.003 PMC4114035

[13] Zeiser, R. et al. Efficacy and safety of itacitinib versus placebo in combination with corticosteroids for initial treatment of acute graft-versus-host disease (GRAVITAS-301): a randomised, multicentre, double-blind, phase 3 trial. *Lancet Haematol.* **9**, e14–e25 (2022). 34971577 10.1016/S2352-3026(21)00367-7

[14] Kekre, N. et al. Phase II trial of natalizumab with corticosteroids as initial treatment of gastrointestinal acute graft-versus-host disease. *Bone Marrow Transplant.* **56**, 1006–1012 (2021). 32895491 10.1038/s41409-020-01049-0

[15] Al Malki, M. M. et al. Phase 2 study of natalizumab plus standard corticosteroid treatment for high-risk acute graft-versus-host disease. *Blood Adv.* **7**, 5189–5198 (2023). 37235690 10.1182/bloodadvances.2023009853 PMC10505783

[16] Lee, S. J. et al. Effect of up-front daclizumab when combined with steroids for the treatment of acute graft-versus-host disease: results of a randomized trial. *Blood* **104**, 1559–1564 (2004). 15138163 10.1182/blood-2004-03-0854

[17] Adkins, D., Ratanatharathorn, V., Yang, H. & White, B. Safety profile and clinical outcomes in a phase I, placebo-controlled study of siplizumab in acute graft-versus-host disease. *Transplantation* **88**, 198–202 (2009). 19623014 10.1097/TP.0b013e3181abfbf7

[18] Rashidi, A., DiPersio, J. F., Sandmaier, B. M., Colditz, G. A. & Weisdorf, D. J. Steroids versus steroids plus additional agent in frontline treatment of acute graft-versus-host disease: a systematic review and meta-analysis of randomized trials. *Biol. Blood Marrow Transplant.* **22**, 1133–1137 (2016). 26970383 10.1016/j.bbmt.2016.02.021 PMC5045896

[19] Hou, C. et al. Ruxolitinib combined with corticosteroids as first-line therapy for acute graft-versus-host disease in haploidentical peripheral blood stem cell transplantation recipients. *Transplant. Cell Ther.* **27**, 75.e1–75.e10 (2021). 32961370 10.1016/j.bbmt.2020.09.015

[20] Zeiser, R. et al. Ruxolitinib for glucocorticoid-refractory acute graft-versus-host disease. *N. Engl. J. Med* **382**, 1800–1810 (2020). 32320566 10.1056/NEJMoa1917635

[21] Jagasia, M. et al. Ruxolitinib for the treatment of steroid-refractory acute GVHD (REACH1): a multicenter, open-label phase 2 trial. *Blood* **135**, 1739–1749 (2020). 32160294 10.1182/blood.2020004823 PMC7229262

[22] Zeiser, R. et al. Ruxolitinib in corticosteroid-refractory graft-versus-host disease after allogeneic stem cell transplantation: a multicenter survey. *Leukemia* **29**, 2062–2068 (2015). 26228813 10.1038/leu.2015.212 PMC4854652

[23] Delgado-Martin, C. et al. JAK/STAT pathway inhibition overcomes IL7-induced glucocorticoid resistance in a subset of human T-cell acute lymphoblastic leukemias. *Leukemia* **31**, 2568–2576 (2017). 28484265 10.1038/leu.2017.136 PMC5729333

[24] Hülsdünker, J. et al. Neutrophils provide cellular communication between ileum and mesenteric lymph nodes at graft-versus-host disease onset. *Blood* **131**, 1858–1869 (2018). 29463561 10.1182/blood-2017-10-812891 PMC5909763

[25] Stickel, N. et al. MicroRNA-146a reduces MHC-II expression via targeting JAK/STAT signaling in dendritic cells after stem cell transplantation. *Leukemia* **31**, 2732–2741 (2017). 28484267 10.1038/leu.2017.137 PMC6231537

[26] Betts, B. C. et al. Targeting JAK2 reduces GVHD and xenograft rejection through regulation of T cell differentiation. *Proc. Natl. Acad. Sci. USA* **115**, 1582–1587 (2018). 29382747 10.1073/pnas.1712452115 PMC5816153

[27] Shi, J. G. et al. The pharmacokinetics, pharmacodynamics, and safety of orally dosed INCB018424 phosphate in healthy volunteers. *J. Clin. Pharm.* **51**, 1644–1654 (2011). 10.1177/0091270010389469 21257798

[28] Mori, Y. et al. Ruxolitinib treatment for GvHD in patients with myelofibrosis. *Bone Marrow Transplant.* **51**, 1584–1587 (2016). 27721370 10.1038/bmt.2016.256

[29] Dou, L. et al. Ruxolitinib-corticosteroid as first-line therapy for newly diagnosed high-risk acute graft versus host disease: study protocol for a multicenter, randomized, phase II controlled trial. *Trials* **23**, 470 (2022). 35668528 10.1186/s13063-022-06426-2 PMC9169300

[30] Mielcarek, M. et al. Effectiveness and safety of lower dose prednisone for initial treatment of acute graft-versus-host disease: a randomized controlled trial. *Haematologica* **100**, 842–848 (2015). 25682602 10.3324/haematol.2014.118471 PMC4450631

[31] Aslanis, V. et al. Multiple administrations of fluconazole increase plasma exposure to ruxolitinib in healthy adult subjects. *Cancer Chemother. Pharm.* **84**, 749–757 (2019). 10.1007/s00280-019-03907-1 31324935

[32] Zhao, Y. et al. Co-administration with voriconazole doubles the exposure of ruxolitinib in patients with hematological malignancies. *Drug Des. Dev. Ther.* **16**, 817–825 (2022). 10.2147/DDDT.S354270 PMC8964335 35370398

[33] Quintás-Cardama, A. et al. Preclinical characterization of the selective JAK1/2 inhibitor INCB018424: therapeutic implications for the treatment of myeloproliferative neoplasms. *Blood* **115**, 3109–3117 (2010). 20130243 10.1182/blood-2009-04-214957 PMC3953826

[34] Ogiya, D. et al. The JAK-STAT pathway regulates CD38 on myeloma cells in the bone marrow microenvironment: therapeutic implications. *Blood* **136**, 2334–2345 (2020). 32844992 10.1182/blood.2019004332 PMC7702477

[35] Zeinalzadeh, E. et al. The role of Janus kinase/STAT3 pathway in hematologic malignancies with an emphasis on epigenetics. *Front. Genet.* **12**, 703883 (2021). 34992627 10.3389/fgene.2021.703883 PMC8725977

[36] Ahmed, A. et al. Ruxolitinib in adult patients with secondary haemophagocytic lymphohistiocytosis: an open-label, single-centre, pilot trial. *Lancet Haematol.* **6**, e630–e637 (2019). 31537486 10.1016/S2352-3026(19)30156-5 PMC8054981

[37] Tefferi, A. & Pardanani, A. Serious adverse events during ruxolitinib treatment discontinuation in patients with myelofibrosis. *Mayo Clin. Proc.* **86**, 1188–1191 (2011). 22034658 10.4065/mcp.2011.0518 PMC3228619

[38] de Kort, E. A., van Dorp, S., Blijlevens, N. & van der Velden, W. Corticosteroid replacement by ruxolitinib in patients with acute GVHD experiencing severe steroid-induced side effects. *Bone Marrow Transplant.* **55**, 253–255 (2020). 30971779 10.1038/s41409-019-0526-0

[39] MacMillan, M. L., DeFor, T. E. & Weisdorf, D. J. The best endpoint for acute GVHD treatment trials. *Blood* **115**, 5412–5417 (2010). 20388871 10.1182/blood-2009-12-258442

[40] Vander Lugt, M. T. et al. ST2 as a marker for risk of therapy-resistant graft-versus-host disease and death. *N. Engl. J. Med.* **369**, 529–539 (2013). 23924003 10.1056/NEJMoa1213299 PMC3943357

[41] Wang, N. et al. High risk of recurrence of malignancy noted in four-day rATG regimen after allogeneic PBSCT from matched sibling donors. *Transplant. Cell Ther.* **28**, 769.e1–769.e9 (2022). 35973670 10.1016/j.jtct.2022.08.012

[42] Wang, H. et al. Targeted dosing of anti-thymocyte globulin in adult unmanipulated haploidentical peripheral blood stem cell transplantation: a single-arm, phase 2 trial. *Am. J. Hematol.* **98**, 1732–1741 (2023). 37706580 10.1002/ajh.27068

[43] Li, H. H. et al. Similar incidence of severe acute GVHD and less severe chronic GVHD in PBSCT from unmanipulated, haploidentical donors compared with that from matched sibling donors for patients with haematological malignancies. *Br. J. Haematol.* **176**, 92–100 (2017). 27714774 10.1111/bjh.14331

