# Supplementary material for: Ruxolitinib plus steroids for acute graft versus host disease: a multicenter, randomized, phase 3 trial
Source: Signal Transduct Target Ther. 2024 Oct 23;9:288. doi: 10.1038/s41392-024-01987-x (PMC11496732; doi:10.1038/s41392-024-01987-x)
Supplement: Supplementary file 1 — Supplementary Materials [file 41392_2024_1987_MOESM1_ESM.docx]

Supplementary Materials for

Ruxolitinib plus steroids for acute graft versus host disease: a multicenter, randomized, phase 3 trial

Liping Dou^1^, Yanli Zhao^2^, Jingjing Yang^1^, Lei Deng^3^, Nan Wang^1^, Xiawei Zhang^1^, Qingyang Liu^1^, Yan Yang^4^, Zhijie Wei^2^, Fuxu Wang^5^,Yifan Jiao^1^, Fei Li^1^, Songhua Luan^1^, Liangding Hu^1^, Sujun Gao^6^, Chuanfang Liu^7^, Xiangjun Liu^8^, Jinsong Yan^4^, Xuejun Zhang^5^, Fang Zhou^3^, Peihua Lu^2^, Daihong Liu^1^

Correspondence to: [daihongrm@163.com](mailto:daihongrm@163.com); peihua_lu@126. com; zhoufang1@medmail.com.cn

**This PDF file includes:**

Materials and Methods

Figures. S1 to S9

Tables S1 to S6

**Materials and Methods**

## Endpoints

Surviving patients were followed up from the date of the graft infusion to December 1, 2023. The time before graft infusion was denoted as "-" days, and the time after graft infusion was denoted as "+" days.

Patients who survived at least 100 days post-transplantation were evaluated for chronic GVHD. The severity of aGVHD was graded based on the modified Glucksberg criteria.^1^ Diagnoses of chronic GVHD were made based on the published criteria. ^2, 3^ Chronic GVHD was stratified as “mild,” “moderate,” or “severe” based on the National Institutes of Health (NIH) criteria.^3, 4^ The criteria for treatment response included CR, PR, or NR. DOR at 6 months was defined as the time from the first response to GVHD progression, recurrence, or death and was assessed in all participants who achieved a CR and remained in the study through day 180 after enrollment. OS was defined as the time from enrollment to death due to any cause. DFS was defined as the time from enrollment to the primary disease relapse or death from any cause, whichever occurred first. Relapse was defined by morphological evidence of disease in the peripheral blood, marrow, or extramedullary sites. NRM was defined as death without evidence of disease progression or relapse, with relapse as a competing event. FFS was defined as the time from enrollment to death, relapse or progression, or the start of new systemic therapy for recurrent aGVHD, whichever came first.

The safety endpoints were determined by the frequency of adverse events (AEs). AEs were as defined according to the National Cancer Institute Common Terminology Criteria for Adverse Events (CTCAE, version 4.0). Cytopenia was identified based on CTCAE grade.

## GVHD prophylaxis and supportive therapy

All recipients of the transplantation received rabbit anti-thymocyte globulin (rATG), cyclosporine A (CsA), mycophenolate mofetil, and short-term methotrexate for GVHD prophylaxis. For patients undergoing haploidentical stem cell transplantation or unrelated donor transplantation, rabbit ATG (Thymoglobulin; Genzyme-Sanofi, Lyon, France) was given intravenously at a total dose of 10 mg/kg from day -5 to day -2, with 1.5 mg/kg given on day -5, 2.5 mg/kg given on day -4, and 2.5 mg/kg given on day -3, and 3.5 mg/kg given on day -2.^5-7^ Patients undergoing matched related donor stem cell transplantation received rabbit ATG (Thymoglobulin; Genzyme-Sanofi, Lyon, France) intravenously at a total dose of 5 mg/kg from day -5 to day -4, with 1.5 mg/kg given on day -5 and 3.5 mg/kg given on day -4.^8^ CsA was given intravenously at a dose of 2 mg/kg from day -10 before transplantation, with a target minimum concentration range of 150–250 ng/mL for the first month. The CsA dose was tapered by 25% every 2 weeks in patients who did not experience relapse within 3–6 months. If a patient relapsed after transplantation, the dose of CsA was promptly tapered. Mycophenolate mofetil was orally administered twice a day at a dose of 500 mg from day -10 to day +30. Methotrexate was infused at a dose of 15 mg/m^2^ on day +1 and at a dose of 10 mg/m^2^ on days +3, +6, and +11. Quantification of EBV and CMV DNA was performed by polymerase chain reaction (PCR) analysis twice weekly until 3 months after transplantation.

## Second-line therapy

For refractory or recurrent aGVHD, basiliximab was used as second-line therapy or alternative drugs such as methotrexate or mesenchymal stem cell infusion were considered based on the researcher's judgment. For refractory aGVHD, methylprednisolone was discontinued, and CsA was continued in both groups. Prolonged maintenance of ruxolitinib was used in the RUX/steroids combined group. A standardized follow-up booklet was given to each enrolled patient for recording the dose, frequency, and administration method of all medical agents.

## GVHD treatment

**Ruxolitinib plus corticosteroids treatment.** The reduction of methylprednisolone in the RUX/steroids combined group followed a gradual tapering schedule over 6 weeks as follows: dosage reductions were performed every 5 days until reaching 0.6 mg/kg/day. Further reductions continued to 0.4 mg/kg/day, 0.3 mg/kg/day, 0.25 mg/kg/day, and 0.18 mg/kg/day. By week 4, the dosage was reduced to 0.1 mg/kg/day. After 5 days, it was further decreased to 0.1 mg/kg every other day. Steroid therapy was finally ceased at week 6. During the trial, drug interactions were examined first before adding new agents to reduce the potential effects of other drugs on the effects of ruxolitinib. The use of fluconazole (Diflucan, Pfizer) at doses exceeding 200 mg/day was prohibited due to its potential to interfere with the metabolism of ruxolitinib.

**Corticosteroids treatment.** The methylprednisolone dosage was gradually decreased after CR and tapered over 10 weeks according to the following schedule: dosage reductions every 7 days to 1 mg/kg/day, 0.6 mg/kg/day, 0.4 mg/kg/day, 0.3 mg/kg/day, 0.25 mg/kg/day, and 0.18 mg/kg/day, then 0.1 mg/kg/day at week 7, 0.1 mg/kg every other day after 5 days, and cessation at week 10.

## Luminex assays for aGVHD biomarker measurement

Samples (diluted 1:2) and standards were run in duplicate, and absorbance was measured with an analyzer (Luminex 200). The results were calculated using versionXponent_4.2 (Luminex 200). The Luminex Assay Human Premixed Multi-Analyte Kit was purchased from R&D Systems (Catalog No. LXSAHM-05) and used for the measurement of aGVHD biomarkers (soluble suppression of tumourigenesis-2 [sST2], regenerating islet-derived protein 3-alpha [REG3a]) according to the manufacturer’s protocol. Tumor necrosis factor receptor 1 (TNFR1), interleukin-6 (IL-6) and interleukin-8(IL-8) were also tested at various time points, including before patients underwent the conditioning regimen, on days 7, 14, 28, 60, and 90 after transplantation, at the onset of aGVHD, and 3–7 days after initiating first-line therapy for aGVHD.

## Staging of aGVHD

**Organ Staging**

| **Stage** | **Skin**  **(active erythema only)** | **Liver (bilirubin,**  **mg/dL)** | **Upper GI** | **Lower GI**  **(stool output per day)** |
| --- | --- | --- | --- | --- |
| 0 | No active (erythematous) GvHD rash | <2 | No or intermittent nausea, vomiting, or anorexia |  |
| 1 | Maculopapular rash <25% BSA | 2–3 | Persistent nausea, vomiting, or anorexia | Adult: 500–999 mL/day |
| 2 | Maculopapular rash 25–50% BSA | 3.1–6 | - | Adult: 1000–1500 mL/day |
| 3 | Maculopapular rash >50% BSA | 6.1–15 | - | Adult: >1500 mL/day |
| 4 | Generalized  erythroderma | >15 | - | Severe abdominal pain with  or without ileus |
|  | (>50% BSA) plus bullous formation and desquamation  >5% BSA |  |  |  |

BSA denotes body surface area; GI, gastrointestinal; GVHD, graft-versus-host disease.

***Overall Clinical Grade***

(Based on most severe target organ involvement)

| **Grade** | **Description** |
| --- | --- |
| 0 | No stage 1–4 or any organ |
| I | Stage 1–2 skin without liver, upper GI or lower GI involvement. |
| II | Stage 1-3 rash and/or stage 1 liver and/or stage 1 upper GI and/or stage 1  lower GI |
| III | Stage 2–3 liver and/or stage 2–4 lower GI with stage 0–3 skin and/or  stage 0–1 upper GI |
| IV | Stage 4 skin or liver, with lower GI involvement, with stage 0–1 upper GI |

GI denotes gastrointestinal

## Response Definitions

(Derived from Thomas et al.^9^)

| **Response** | **Description** |
| --- | --- |
| Complete response | Score of 0 for aGvHD grading in all evaluable organs, indicating complete resolution of all signs and symptoms of aGvHD in all evaluable organs without administration of additional systemic therapies for any earlier progression, mixed response, or non- response of aGvHD |
| Partial response | Improvement of 1 stage in 1 or more organs involved with aGvHD signs or symptoms without progression in other organs or sites without administration of additional systemic therapies for an earlier progression, mixed response, or non-response of aGvHD |
| No response | Absence of improvement in any organ involved with aGvHD, without worsening in any involved organ； Worsening in 1 or more organs by 1 or more stages |

aGVHD denotes acute graft-versus-host disease.

**Response rate** is defined as the proportion of patients with complete or partial response.

**Lack of response** is defined as no response or progression.


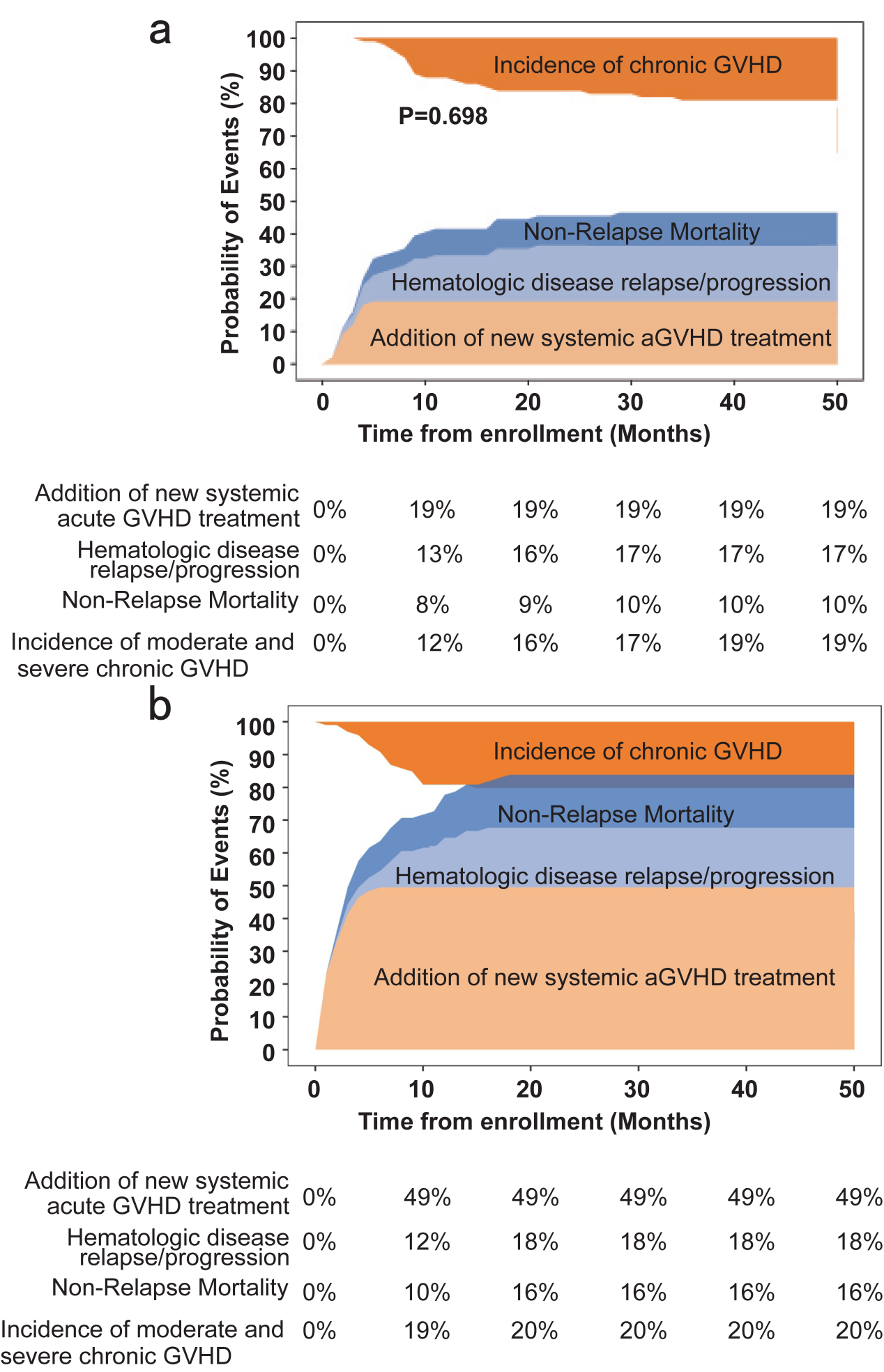


**Figure. S1. Median Failure Free Survival.**

RUX/steroids denotes ruxolitinib combined with steroids therapy; Events include hematologic disease relapse/progression, non-relapse mortality, or addition of systemic aGVHD treatment. GVHD, graft versus host disease. (a) RUX/steroids group. (b) Steroids only group.

##

##
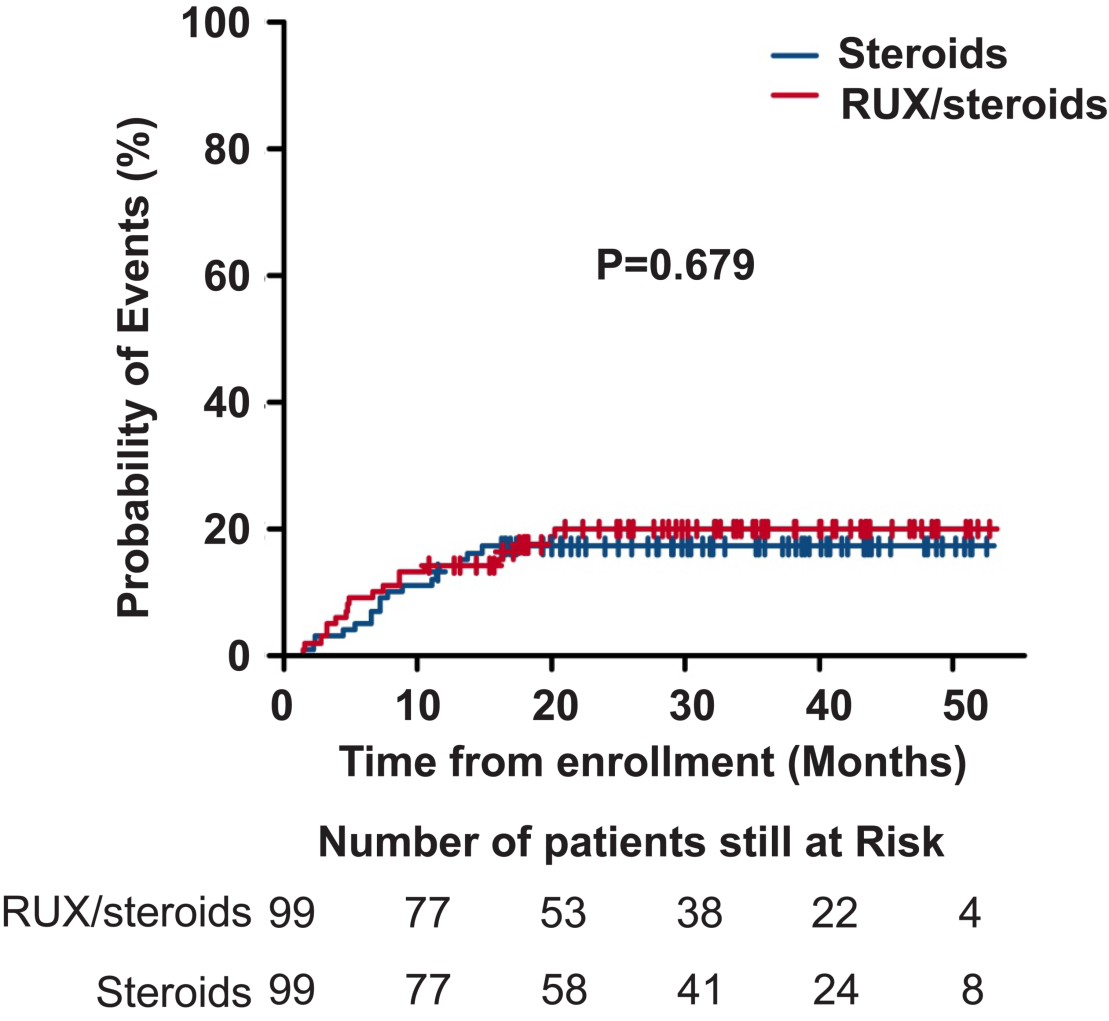


## Figure S2. Cumulative Incidence of Malignancy Relapse/Progression.

RUX/steroids denotes ruxolitinib combined with steroids therapy; Steroids denotes steroids only therapy. Competing risk was non–relapse-related death.

##
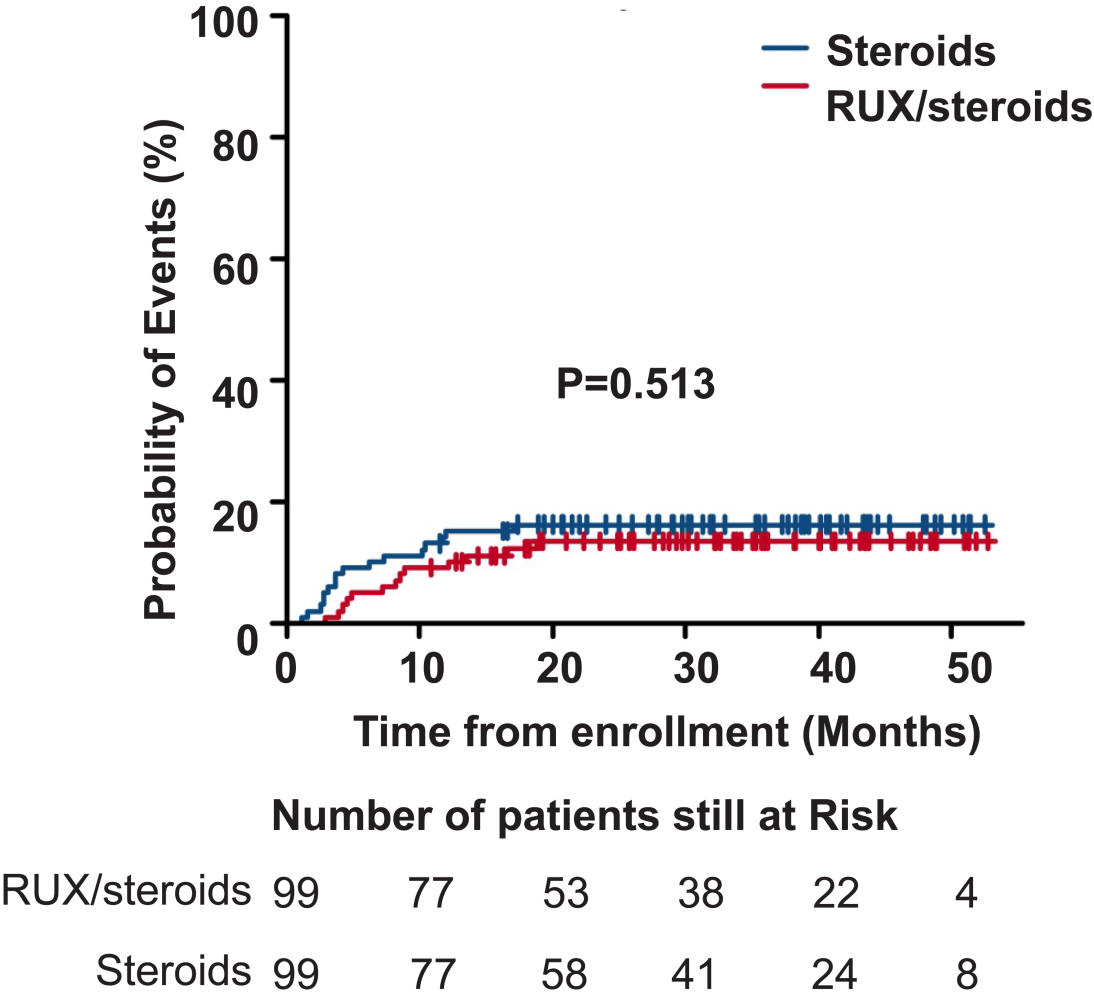


## Figure S3. Non-Relapse Mortality.

RUX/steroids denotes ruxolitinib combined with steroids therapy; Steroids denotes steroids only therapy. Competing risk was hematological disease relapse/progression.


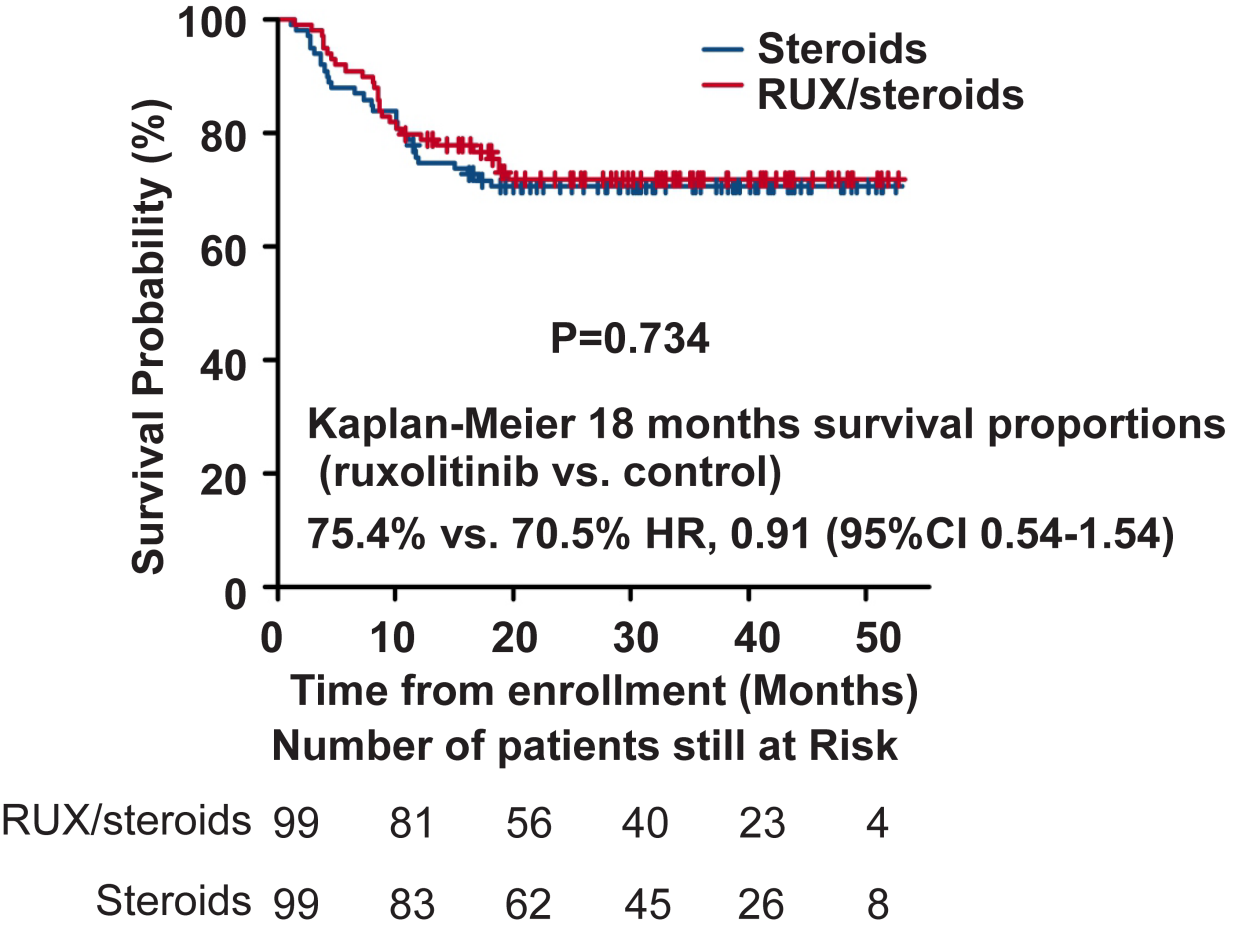


## Figure S4. Overall Survival.

RUX/steroids denotes ruxolitinib combined with steroids therapy; Steroids denotes steroids only therapy. HR, hazard ratio.

**
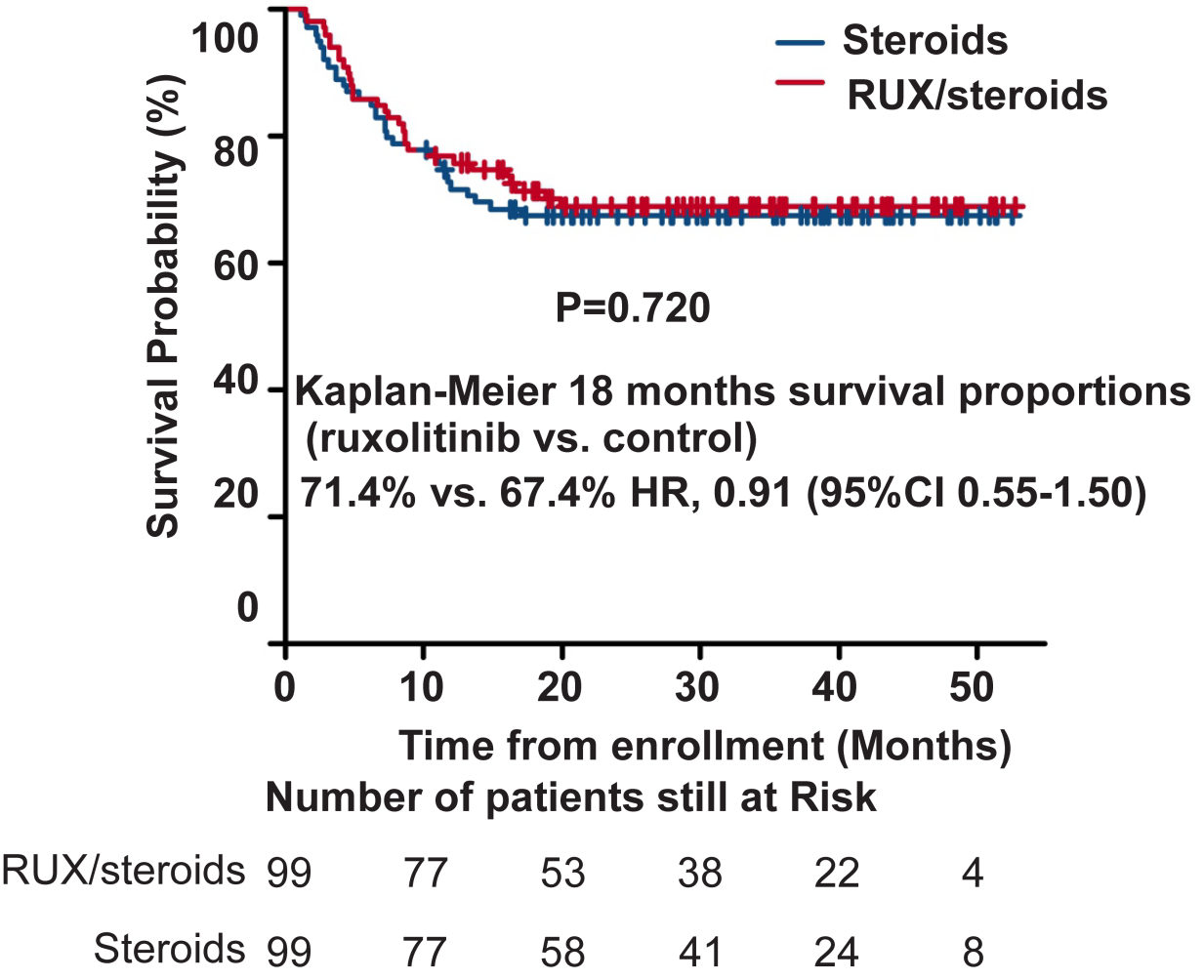
**

## Figure S5. Disease Free Survival.

RUX/steroids denotes ruxolitinib combined with steroids therapy; Steroids denotes steroids only therapy. HR, hazard ratio.

**
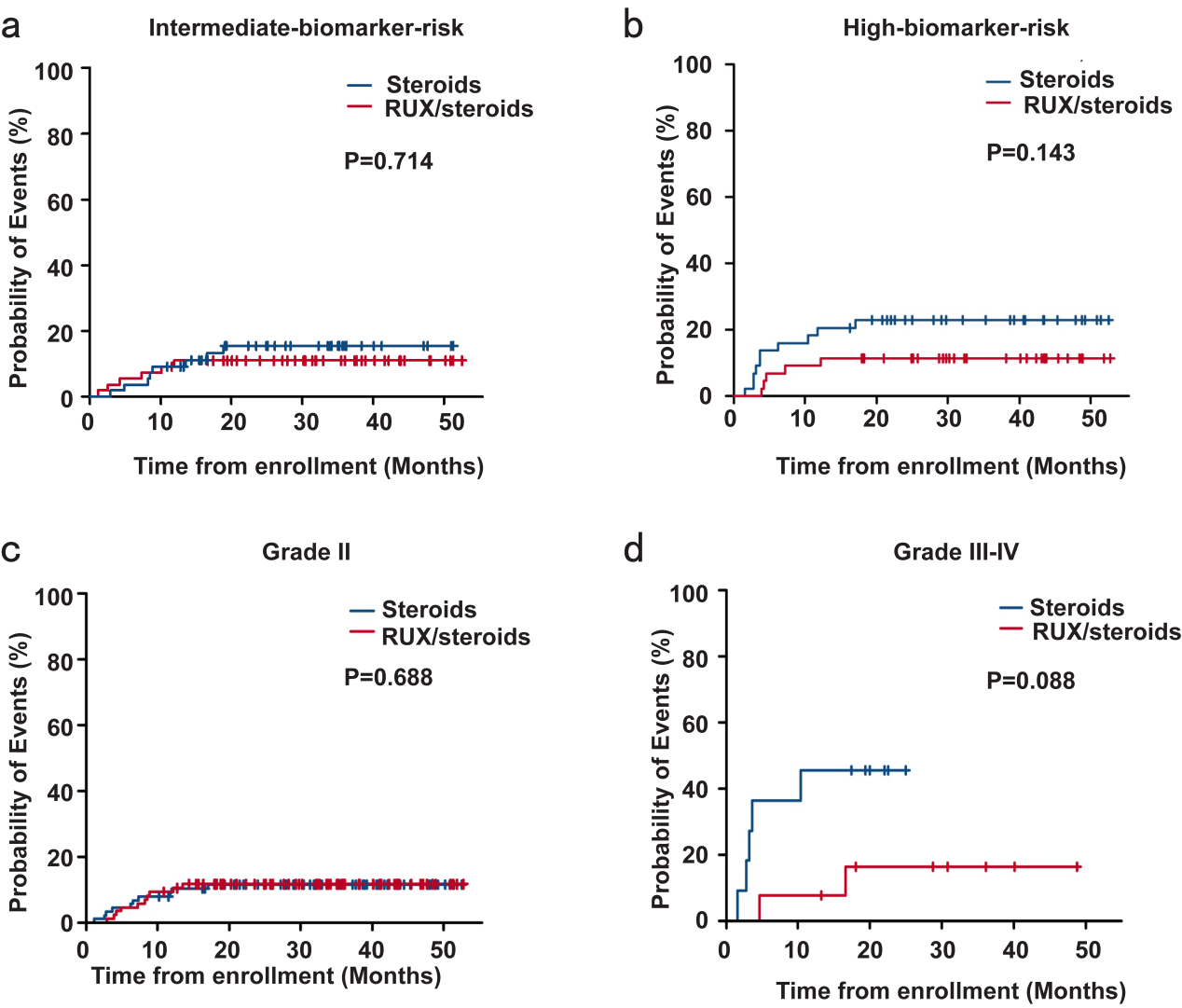
**

## Figure S6. Non-Relapse Mortality.

RUX/steroids denotes ruxolitinib combined with steroids therapy; Steroids denotes steroids only therapy. (a) Non-Relapse Mortality for patients developing acute GVHD of Intermediate-biomarker-risk. (b) Non-Relapse Mortality for patients developing acute GVHD of high-biomarker-risk. (c) Non-Relapse Mortality for patients developing acute GVHD of grade II. (d) Non-Relapse Mortality for patients developing acute GVHD of grade III-IV.

**
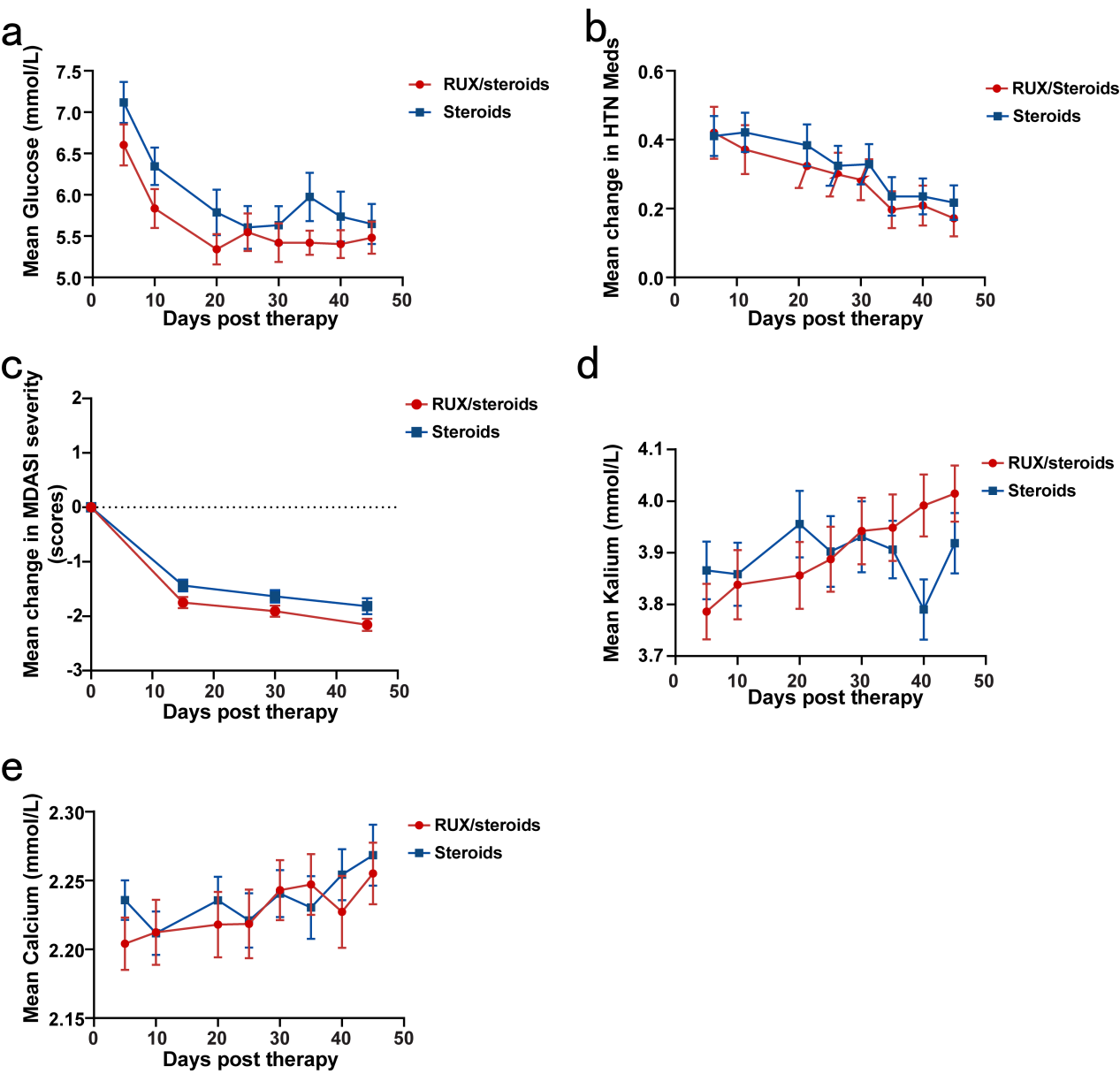
FigureS7. Measures of prednisone-associated toxicity among all patients with first-line therapy.**

(a) Mean blood glucose concentrations. (b) Mean change in number of anti-hypertensive (HTN) medications. This figure showed the mean change in the number of anti-hypertensive medications from baseline during the treatment for the two patient groups. (c) Mean change in quality of life assessed by MD Anderson Cancer Center Symptom inventory (MDASI). Measures of MDASI were assessed at baseline and then every 15 days after starting first-line treatment. (d) Mean blood kalium concentrations. (e) Mean blood calcium concentrations. RUX/steroids denotes ruxolitinib combined with steroids therapy; Steroids only denotes steroids only therapy. RUX, ruxolitinib.

## Figure S8. Changing trend of biomarkers levels in aGVHD patients with first-line therapy.

a-b, the biomarkers levels on Day 14 and 28 in CR patients with RUX/steroids (a) or steroids only (b) as first-line therapy;c-d, the biomarkers levels on Day 14 and 28 in Re patients with RUX/steroids (c) or steroids only (d) as first-line therapy; e-f, the biomarkers levels on Day 14 and 28 in CR and Refractory patients with RUX/steroids (e) or steroids only (f) as first-line therapy. RUX/steroids denotes ruxolitinib combined with steroids therapy; Steroids only denotes steroids only therapy. CR, efficacy was evaluated as complete remission at day 3, day 7, day 14 and day 28 after first therapy; Re, refractory, efficacy was evaluated as (i) the progression of GVHD at least 3 days after first therapy; (ii) a lack of improvement in GVHD (PR or better) at least 7 days after first therapy; (iii) no complete remission at least 14 days first therapy; or (iv) a loss of response; ST2, suppression of tumorigenicity 2; REG3α, regenerating islet-derived protein 3-alpha; IL-6, interleukin-6; IL-8, interleukin-8; TNFR1, TNF receptor 1; RUX, ruxolitinib; *, P<0.05; **, P≤0.01; ***, P≤0.001.
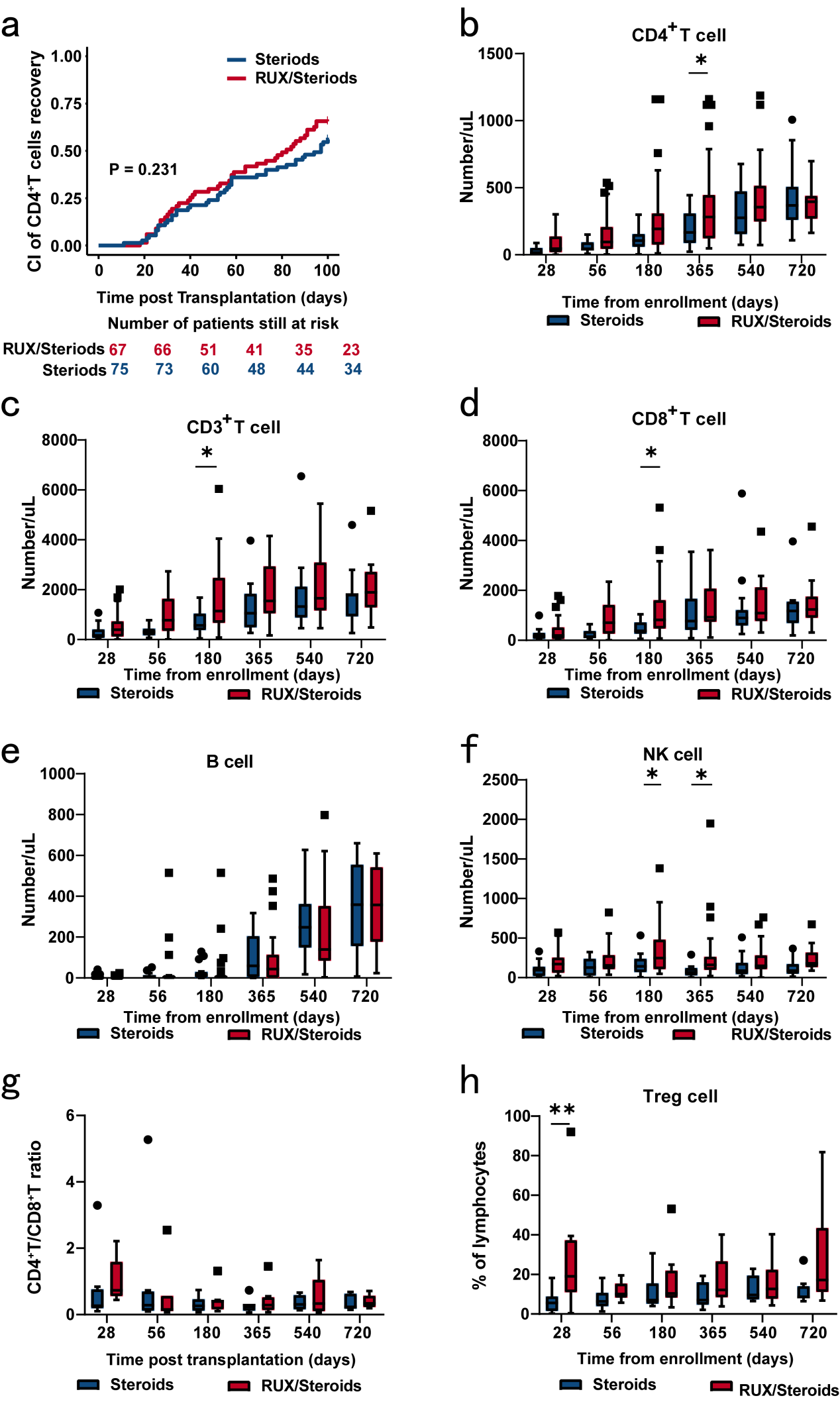
Figure S9. Immune reconstitution of aGVHD patients with first-line therapy.

(a) CD4^+^T cell immune reconstitution +100 days after transplantation; the difference values of Treg (b), CD3^+^T (c), CD8^+^ T (d), B (e)and NK cells(f) were evaluated on 28, 56, 180, 365, 540 and 720 days after enrollment, with their pre-enrollment absolute numbers as baseline. The ratio of CD4^+^T cell/CD8^+^T cells were evaluated on 28, 56, 180, 365, 540 and 720 days after enrollment (g); the difference values of Treg cells were evaluated on 28, 56, 180, 365, 540 and 720 days after enrollment, with their pre-enrollment percentages of lymphocytes as baseline(h). RUX/steroids denotes ruxolitinib combined with steroids therapy; Steroids only denotes steroids only therapy. RUX, ruxolitinib; ∆, difference value; *, *P*<0.05; **, *P*<0.01.

**Table S1.**

**Supplemental Table 1. Acute GVHD biopsy information**

|  | Ruxolitinib/steroids  (N=99) | Steroids  (N=99) | Total  (N=198) |
| --- | --- | --- | --- |
| **Skin Biopsy Results (N=135)** | 63 | 72 | 135 |
| Positive | 4 (6.3%) | 5 (6.9%) | 9 (6.7%) |
| Negative | 1 (1.6%) | 1 (1.4%) | 2 (1.5%) |
| Equivocal | 1 (1.6%) | 0 (0.0%) | 1 (0.7%) |
| Not Done | 57 (90.5%) | 66 (91.7%) | 123 (91.1%) |
| **Upper GI Biopsy Results (N=85)** | 47 | 38 | 85 |
| Positive | 5 (10.6%) | 5 (13.2%) | 10 (11.8%) |
| Negative | 2 (4.3%) | 2 (5.3%) | 4 (4.7%) |
| Equivocal | 1 (2.1%) | 0 (0.0%) | 1 (1.2%) |
| Not Done | 39 (83.0%) | 31 (81.6%) | 70 (82.4%) |
| **Lower GI Biopsy Results (N=54)** | 24 | 30 | 54 |
| Positive | 15 (62.5%) | 15 (50.0%) | 30 (55.6%) |
| Negative | 1 (4.2%) | 2 (6.7%) | 3 (5.6%) |
| Equivocal | 2 (8.3%) | 3 (10.0%) | 5 (9.3%) |
| Not Done | 6 (25%) | 10 (33.3%) | 16 (29.6%) |
| **Liver Biopsy Results (N=13)** | **6** | 7 | 13 |
| Not Done | 6 (100%) | 7 (100%) | 13 (100%) |

**Table S2.**

## Supplemental Table 2. Second-line therapies used for recurrent aGVHD

| Any posttreatment therapy, n (%) | Ruxolitinib/steroids  (N=25) | Steroids  (N=54) | Total  (N=79) |
| --- | --- | --- | --- |
| Ruxolitinib | 5(20.0) | 32(59.3) | 37(46.8) |
| Methylprednisolone | 2(8.0) | 3(5.6) | 5(6.3) |
| Mycophenolate mofetil | 5(20.0) | 2(3.7) | 7(8.9) |
| Tacrolimus | 2(8.0) | 1(1.9) | 3(3.8) |
| Mesenchymal stem cells | 3(12.0) | 11(20.4) | 14(17.7) |
| Etanercept | 0 | 2(3.7) | 2(2.5) |
| Sirolimus | 4(16.0) | 7(13.0) | 11(13.9) |
| Methotrexate | 3(12.0) | 3(5.6) | 6(7.6) |
| Basiliximab | 8(32.0) | 11(20.4) | 19(24.1) |
| Cyclosporine | 1(4.0) | 1(1.8) | 2(2.5) |
| Pomalidomide | 0 | 2(3.7) | 2(2.5) |

**Table S3.**

## Supplemental Table 3. Primary causes of death

| Primary cause of death, n (%) | Ruxolitinib/steroids  N=99 | Steroids  N=99 |
| --- | --- | --- |
| Patient deaths | 24(24.2) | 27(27.3) |
| Infections | 4(4.0) | 7(7.1) |
| Malignancy relapse | 13(13.1) | 12(12.1) |
| Acute GVHD | 2(2.0) | 5(5.1) |
| Haemorrhage/vascular | 2(2.0) | 0 |
| Thrombotic microangiopathy | 2(2.0) | 0 |
| Alveolar hemorrhage | 0 | 0 |
| Organ failure not related to GVHD or infection | 3(3.0) | 3(3.0) |
| Multiple organ failure | 3(3.0) | 2(2.0) |
| Sinusoidal obstruction syndrome | 0 | 1(1.0) |

**Table S4.**

## Supplemental Table 4. Systemic infections reported on trial according to study arm

|  |  | Ruxolitinib/steroids  N=99 | Steroids  N=99 |
| --- | --- | --- | --- |
| Total number of infections |  | 84（84.8%） | 85（85.9%） |
| Infection site | Blood | 2（2.0%） | 4（4.0%） |
|  | Brain | 2(2.0) | 4（4.0） |
|  | Intestine | 14(14.1) | 11（11.1） |
|  | Lung | 43(43.4) | 39（39.4） |
|  | urinary tract | 26(26.3) | 18(18.2) |
|  | Skin | 2(2.0) | 8(8.1) |
|  | Upper respiratory tract | 12(12.1) | 5(5.1) |
|  | Other | 0 | 2(2.0) |
|  |  |  |  |
| Time of onset post randomization | Median days |  |  |
|  | 0-90 | 77(77.8) | 76(76.8) |
|  | 91-180 | 7(7.1) | 4(4.0) |
|  | 18-270 | 0 | 0 |
|  | 271-365 | 0 | 5(5.1) |
|  |  |  |  |
| Infection severity | Grade 1-2 | 41(41.4) | 10(10.1) |
|  | Grade3 | 43(43.4) | 65（65.7） |
|  | Grade4 | 0 | 10(10.1) |

**Table S5.**

## Supplemental Table 5. Grade of systemic infections reported on trial according to study arm

| Type of infection  Maximum severity grade | Ruxolitinib/steroids  N=99 | Steroids  N=99 |
| --- | --- | --- |
| Number of patients with at least 1 event | 84（84.8%） | 85（85.9%） |
| Grade1 | 0 | 2（2.0%） |
| Grade2 | 41（41.4%） | 13（13.1%） |
| Grade3 | 41（41.4%） | 59（59.6%） |
| Grade4 | 2（2.0%） | 11（11.1%） |
|  |  |  |
| Fungal infections | 21（21.2%） | 21（21.2%） |
| Grade1 | 0 | 0 |
| Grade2 | 7（7.1%） | 2（2.0%） |
| Grade3 | 14（14.1%） | 17（17.2%） |
| Grade4 | 0 | 2（2.0%） |
|  |  |  |
| Viral infection | 79（79.8%） | 75（75.8%） |
| Grade1 | 1(1.0%) | 2（2.0%） |
| Grade2 | 38（38.4%） | 13（13.1%） |
| Grade3 | 38（38.4%） | 56（56.6%） |
| Grade4 | 2（2.0%） | 4（4.0%） |
|  |  |  |
| Bacterial infection | 41（41.4%） | 41（41.4%） |
| Grade1 | 0 | 0 |
| Grade2 | 12（12.1%） | 13（13.1%） |
| Grade3 | 29（29.3%） | 24（24.2%） |
| Grade4 | 0 | 4（4.0%） |

**Table S6.**

## Supplemental Table 6. List of Abbreviations

The following abbreviations and special terms were used in this article.

| **Abbreviation** | **Definition** |
| --- | --- |
| AE | adverse event |
| aGVHD | acute graft versus host disease |
| cGVHD | chronic graft versus host disease |
| CI | confidence interval |
| CMV | cytomegalovirus |
| CR | complete response |
| CsA | cyclosporine A |
| CTCAE | Common Terminology Criteria for Adverse Events |
| CYP450 | cytochrome P450 |
| DFS | disease-free survival |
| DNA | deoxyribonucleic acid |
| DOR | duration of response |
| EBV | Epstein–Barr virus |
| FFS | failure-free survival |
| GVHD | graft versus host disease |
| HR | hazard ratio |
| IL | interleukin |
| IQR | interquartile range |
| JAK | Janus kinase |
| NIH | National Institutes of Health |
| NR | no response |
| NRM | non-relapse mortality |
| OR | odd ratios |
| ORR | overall response rate |
| OS | overall survival |
| PCR | polymerase chain reaction |
| PR | partial response |
| rATG | rabbit anti-thymocyte globulin |
| REG3α | regenerating islet-derived 3-alpha |
| RUX | ruxolitinib |
| SD | standard deviation |
| SR | steroid-refractory |
| sST2 | soluble suppression of tumorigenesis-2 |
| TNFR1 | tumor necrosis factor receptor 1 |

# Supplementary References

1. Dou L, Peng B, Li X, et al. Ruxolitinib-corticosteroid as first-line therapy for newly diagnosed high-risk acute graft versus host disease: study protocol for a multicenter, randomized, phase II controlled trial. Trials. 2022. 23(1): 470.

2. Yang J, Peng B, Wang L, et al. Elevated REG3α predicts refractory aGVHD in patients who received steroids-ruxolitinib as first-line therapy. Ann Hematol. 2022. 101(3): 621-630.

3. Lee SJ, Vogelsang G, Gilman A, et al. A survey of diagnosis, management, and grading of chronic GVHD. Biol Blood Marrow Transplant. 2002. 8(1): 32-9.

4. Filipovich AH, Weisdorf D, Pavletic S, et al. National Institutes of Health consensus development project on criteria for clinical trials in chronic graft-versus-host disease: I. Diagnosis and staging working group report. *Biol Blood Marrow Transplant*. 2005;11(12):945-956.

5. Li HH, Li F, Gao CJ, et al. Similar incidence of severe acute GVHD and less severe chronic GVHD in PBSCT from unmanipulated, haploidentical donors compared with that from matched sibling donors for patients with haematological malignancies. *Br J Haematol*. 2017;176(1):92-100.

6. Admiraal R, Nierkens S, de Witte MA, et al. Association between anti-thymocyte globulin exposure and survival outcomes in adult unrelated haemopoietic cell transplantation: a multicentre, retrospective, pharmacodynamic cohort analysis. *Lancet Haematol*. 2017;4(4):e183-e191.

7. Li HH, Li F, Gao CJ, et al. Similar incidence of severe acute GVHD and less severe chronic GVHD in PBSCT from unmanipulated, haploidentical donors compared with that from matched sibling donors for patients with haematological malignancies. Br J Haematol. 2017. 176(1): 92-100.

8. Wang N, Wang H, Fang S, et al. High Risk of Recurrence of Malignancy Noted in Four-day rATG Regimen After Allogeneic PBSCT From Matched Sibling Donors. Transplant Cell Ther. 2022. 28(11): 769.e1-769.e9.

9. Thomas ED, Storb R, Clift RA, et al. Bone-marrow transplantation (second of two parts). N Engl J Med. 1975. 292(17): 895-902.
