# Supplementary material for: Ruxolitinib plus steroids for acute graft versus host disease: a multicenter, randomized, phase 3 trial
Source: Signal Transduct Target Ther. 2024 Oct 23;9:288. doi: 10.1038/s41392-024-01987-x (PMC11496732; doi:10.1038/s41392-024-01987-x)
Supplement: Supplementary file 2 — Study Protocol [file 41392_2024_1987_MOESM2_ESM.docx]

**Clinical Study Protocol**

**Ruxolitinib-Corticosteroid as First-Line Therapy for Newly Diagnosed High-risk Acute Graft Versus Host Disease: A Multicenter, Randomized, Phase II Controlled Trial**

| **Product:** | **Ruxolitinib** |
| --- | --- |
| **ID Number:** | **S2019-177** |
| **ClinicalTrials.gov Number:** | **04061876** |
| **Phase of Study:** | **2** |
| **Sponsor:** | **Chinese PLA General Hospital** |
| **Original Protocol (Version 1):** | **18 May 2019** |
| **Amendment (Version 2):** | **23 May 2022** |
| **Study Start:** | **25 Aug 2019** |
| **Primary Completion:** | **1 July 2022** |

**The study protocol was published in the journal of Trials in 2022 (doi: 10.1186/s13063-022-06426-2).**

| **This study will be performed in accordance with ethical principles that have their origin in the Declaration of Helsinki and conducted in adherence to the study Protocol, Good Clinical Practices, as well as ICH GCP consolidated guidelines (E6) and applicable regulatory requirements.** |
| --- |

**INVESTIGATOR'S AGREEMENT**

I have read the study protocol of **Ruxolitinib-Corticosteroid as First-Line Therapy for Newly Diagnosed High-risk Acute Graft Versus Host Disease: A Multicenter, Randomized, Phase II Controlled Trial** (Version 2 dated 23 MAY 2022) and agree to conduct the study as outlined. I agree to maintain the confidentiality of all information received or developed in connection with this Protocol.

(Printed Name of Investigator)

(Signature of Investigator) (Date)

# SYNOPSIS

| **Name of Investigational Product:** Ruxolitinib | |
| --- | --- |
| **Title of Study:** Ruxolitinib-Corticosteroid as First-Line Therapy for Newly Diagnosed High-risk Acute Graft Versus Host Disease: A Multicenter, Randomized, Phase II Controlled Trial | |
| **Protocol Number:** S2019-177 | **Study Phase:** 2 |
| **Indication:** Acute graft-versus-host disease | |
| \| **Primary Objective** \| **Primary Endpoint** \| \| --- \| --- \| \| Compare the efficacy of ruxolitinib in combination with corticosteroids versus corticosteroids in terms of overall response rate (ORR) at Day 28 in subjects with acute graft-versus-host disease (acute GVHD). \| ORR at Day28, defined as the proportion of participants demonstrating a complete response (CR), or partial response (PR) \| \| **Secondary Objective** \| **Secondary Endpoints** \| \| Compare additional response and longer-term efficacy outcomes between treatment cohorts. \| Durable ORR at Day56, defined as the proportion of patients in each arm whose response on day 28 lasts until day 56 after randomization. \| \| DOR at Month 6 for responders will be calculated. The DOR is defined from the time of the onset of response to loss of response. Subjects who died or discontinued will be censored at the  death date or the previous assessment. \| \| Response duration, defined as the time from the first response to the first recurrence/progression of acute GVHD or additional immunosuppressants for GVHD. \| \| NRM at Month 24, defined as the time from enrollment to death due to any causes other than hematologic disease relapse. \| \| Cumulative incidence of relapse (CIR) at Month 24, defined as the proportion of subjects whose underlying hematologic disease relapses. \| \| Disease-free survival (DFS) at Month 24, defined as the time from enrollment to relapse of primary disease or death from any causes, whichever occurred first. \| \| Failure-free survival (FFS) at Month 24, defined as the time from randomization to disease relapse or progression, non-relapse mortality, or the addition of new therapy for acute GVHD. \| \| Overall survival (OS) at Month 24, defined as the time from randomization to death due to any causes. \| \| Assess the incidence and severity of adverse events (AEs) and serious adverse events. \| Clinical safety data (eg, AEs, infections) will be tabulated and listed. \| \| Evaluate the use and discontinuation of  corticosteroids. \| Average and cumulative corticosteroid dose at Days 365; \| \| Evaluate the use and discontinuation of  immunosuppressive medications. \| Proportion of subjects who discontinue immunosuppressive medications at Days365. \| \| Evaluate the incidence of recurrent acute GVHD \| Incidence rate of recurrent acute GVHD through Month 24. \| \| Evaluate the incidence of cGVHD. \| Incidence rate of cGVHD at Month 24. \| \| **Overall Study Design:**  This trial is an open-label, multicenter, prospective randomized two-arm phase II study comparing the efficacy and safety of ruxolitinib plus methylprednisolone (1 mg/kg) vs. methylprednisolone (2 mg/kg) alone. The biomarkers of acute GVHD, such as sST2, sTNFR1, IL-6, IL-8, and REG3α will be tested at acute GVHD onset. Patients with newly diagnosed intermediate-risk or high-risk acute GVHD, as defined by Minnesota acute GVHD risk score and the biomarker score will be included. The treatment efficacy will be evaluated with respect to the level of biomarkers and clinical manifestations. Written informed consent must be obtained from the patients before enrollment in the study. The consent form and related materials are available from the corresponding author on request.  **Study Population:**  Eligible patients with intermediate-risk or high-risk acute GVHD graded according to the modified Glucksberg criteria, Minnesota acute GVHD risk score and the biomarker score and untreated with systemic acute GVHD therapy will be enrolled in this study.  **Key Inclusion Criteria:**   - Male or female, 14 to 65 years old. - Have undergone first allogeneic hematopoietic stem cell transplantation (allo-HSCT) from any donor source using bone marrow, peripheral blood stem cells, or cord blood for hematologic malignancies. - Develop a de novo acute GVHD, defined as intermediate- or high-risk using the Minnesota acute GVHD risk score and MAGIC biomarker algorithm.   **Key Exclusion Criteria:**   - Has received more than one allogeneic stem cell transplant. - Female patients with pregnant or breast-feeding. - Has Acquired Immune Deficiency Syndrome (AIDS), or hepatitis B/C infection, uncontrolled bacterial, fungal, or viral infections. - Allergies to ruxolitinib. - Vital organ dysfunction unrelated to GVHD, including persistent bilirubin abnormalities or severe heart and respiratory diseases. - Relapse of the primary disease or graft rejection after transplantation. - Presence of GVHD overlap syndrome. - Presence of cGVHD or associated clinical signs or DLI-induced acute GVHD or interferon. - Previous history of JAK1/2 inhibitor treatment after transplantation, except JAK inhibitors administered before the transplantation. - absolute neutrophil count (ANC) <0.5×10^9^/L or platelet count (PLT) < 20×10^9^/L - Serum creatinine > 2.0 mg/dL or creatinine clearance < 40 mL/min measured or calculated by Cockroft-Gault equation. - Patients with poor compliance should not be included in the study based on the judgment of the research team, for example if they refused to take medicine at will many times during the previous treatment.   **Study Drug, Dosage, and Mode of Administration:**  **Ruxolitinib plus corticosteroids treatment (ruxolitinib group):**  The initial dose of methylprednisolone is 1 mg/kg/day intravenously given in this arm for at least 7 days. The dosage of ruxolitinib is 5 mg PO QD. CSA will be administered intravenously at a dose of 2 mg/kg twice daily, targeting minimum concentration levels of 150–250 ng/mL. If GVHD response to treatment is assessed as PR/CR at 7 days, methylprednisolone will be tapered, followed by reduction and discontinuation of CSA and then ruxolitinib. The reduction of the methylprednisolone in the ruxolitinib group is gradual, suggesting it be tapered off over 6 weeks as follows: dosage reduced to 0.6 mg/kg/day, 0.4 mg/kg/day, 0.3 mg/kg/day, 0.25 mg/kg/day, and 0.18 mg/kg every 5 days, followed by 0.1 mg/kg/day at week 4, 0.1 mg/kg every other day after 5 days and stopped at week 6. After steroid discontinuation and CR or stable PR maintained for 6 weeks, CSA is tapered over 60 days. After CSA discontinuation and no presence of recurrent GVHD, ruxolitinib is tapered off over 90 days, which is totally maintained for approximately 6 months.  **Corticosteroid treatment (corticosteroids group):**  The initial dose of methylprednisolone is 2 mg/kg/day given twice daily for at least 7 days and then reduced. The dosage of methylprednisolone in the corticosteroids group is decreased gradually after CR and tapered off over 10 weeks as the following: dosage reduced to 1 mg/kg/day to 0.6 mg/kg/day, 0.4 mg/kg/day, 0.3 mg/kg/day, 0.25 mg/kg/day, and 0.18 mg/kg every 7 days, and then 0.1 mg/kg/day at week 4, 0.1 mg/kg every other day after 5 days, and stopped at week 10. CsA was administered intravenously at a dose of 2 mg/kg twice daily, targeting minimum concentration levels of 150–250 ng/mL. The recommended duration of CsA is 6 months.  In both arms, for refractory acute GVHD, i.e., the progression of GVHD after 3 days of therapy, no improvement within 7 days, or no CR after 14 days of therapy, the second-line therapy will be started. For refractory acute GVHD or recurrent acute GVHD, basiliximab may be used as second-line therapy, and other alternative drugs included methotrexate or mesenchymal stem cells may be used at the researcher’s decision. For refractory acute GVHD, methylprednisolone is discontinued and CsA is continued in both arms. Prolonged maintenance of ruxolitinib is used in the ruxolitinib group. A standardized follow-up booklet is given to each enrolled patient for recording the dose, frequency, and administration method of the drugs. **Estimated Duration of Participation:** Subject participation is expected to average 24 months, which includes the following:   - A screening period lasting up to 28 days. - A treatment period lasting as long as the subject is benefiting from treatment. - A safety follow-up period lasting 180 days after treatment ends. - A survival follow-up period lasting until death or study withdrawal. \| \| \| **Estimated Number of Subjects:** 198 subjects. \| \| \| **Principal Coordinating Investigator:** Daihong Liu \| \| \| **Statistical Methods:**  The sample size is calculated according to the primary endpoint (ORR) of the study. Based on our published phase I study of 32 acute GVHD patients who received steroids as first-line therapy, an expected proportion of 55% for the patients treated with corticosteroids only was established. Based on our published data of patients with acute GVHD grade I–IV who received steroid-ruxolitinib (5 mg/day) as first-line therapy, the ORR was 82.05%. Patients with acute GVHD grade I were also included in the phase I analysis. Thus, an expected proportion of 75% for the patients treated with steroid-ruxolitinib (5mg/day) was established. This study is planned to detect a response difference between treatment arms at a two-sided significance level α=5% with a power of 1-β=80%. The sample size was estimated using PASS software based on the primary endpoint (ORR). Allowing a withdrawal rate of 10%, 198 patients (99/group) will be required.  Continuous data are described as median with interquartile range (IQR) or mean and standard deviation (SD) according to the normality of distribution. The categorical data are described as n (%). The ORR was calculated with its 95% confidence interval (CI) and compared by the stratified Cochran–Mantel–Haenszel test. Additionally, a logistic regression model, which included aGVHD risk (high vs. intermediate), and disease status before transplantation (CR vs. no CR) as covariates, was established to explore the association between treatment and ORR on day 28. Post-hoc subgroup analysis was performed by biomarker risk, age and GVHD grade, although these were not prespecified in the protocol. The cumulative incidence of recurrent aGVHD was analysed using the Fine and Gray test in a competing risk framework. The Kaplan–Meier method was used to estimate the ORR on day 56, OS, DFS, and FFS, and the log-rank test was used to evaluate the difference between the groups. The cumulative incidence rates of NRM and relapse were estimated using a competing risk model and compared using the Fine and Gray test. A Cox proportional hazard regression model was used for multivariable regression analysis for OS and DFS. Multivariable regression analysis was performed for NRM using the Fine–Gray proportional hazard regression for competing events. The following variables were included in the univariable analysis: primary disease, cytogenetic risk, age and gender of the donor and recipient, source of graft, treatment arm, aGVHD risk, and disease status before transplantation. Variables with P<0.1 in univariate analysis or variables known to influence the outcomes were included in the multivariable analysis. Competing events were defined as follows: for GVHD, death without the event; for relapse, death without relapse; and for NRM, relapse. Any difference for which the two-sided P<0.05 was considered statistically significant. All analyses were carried out using SPSS 22.0 software (IBM Corporation, Armonk, NY, USA) or R version 4.1.2 (www.cran.r-project.org). All statistical analyses were based on the intend to treat set. \| \| | |

**TABLE OF CONTENTS**

[SYNOPSIS 3](#_Toc132729885)

[LIST OF ABBREVIATIONS 13](#_Toc132729886)

[1. INTRODUCTION 16](#_Toc132729887)

[1.1. Overview of Acute Graft-Versus-Host Disease 16](#_Toc132729888)

[1.2. MAGIC Algorithm Probability 17](#_Toc132729889)

[1.3. Ruxolitinib Background and Study Rationale 17](#_Toc132729890)

[1.4. Potential Risks and Benefits of the Treatment Regimen 20](#_Toc132729891)

[2. STUDY OBJECTIVES AND ENDPOINTS 21](#_Toc132729892)

[3. SUBJECT ELIGIBILITY 24](#_Toc132729893)

[3.1. Subject Inclusion Criteria 24](#_Toc132729894)

[3.2. Subject Exclusion Criteria 24](#_Toc132729895)

[3.3. Lifestyle Considerations 25](#_Toc132729896)

[3.3.1. Meals and Dietary Restrictions 25](#_Toc132729897)

[3.3.2. Activity 26](#_Toc132729898)

[4. INVESTIGATIONAL PLAN 27](#_Toc132729899)

[4.1. Overall Study Design 27](#_Toc132729900)

[4.2. Recruitment procedures 27](#_Toc132729901)

[4.3. GVHD biomarker Luminex assays 28](#_Toc132729902)

[4.4. Randomization 29](#_Toc132729903)

[4.5. Number of Subjects 29](#_Toc132729904)

[4.5.1. Planned Number of Subjects 29](#_Toc132729905)

[4.5.2. Statistical Rational for the Sample Size 30](#_Toc132729906)

[4.5.3. Replacement of Subjects 30](#_Toc132729907)

[4.6. Duration of Treatment and Subject Participation 30](#_Toc132729908)

[4.7. Overall Study Duration 31](#_Toc132729909)

[4.8. Study Termination 31](#_Toc132729910)

[5. TREATMENT 32](#_Toc132729911)

[5.1. Study Drugs 32](#_Toc132729912)

[5.1.1. Ruxolitinib plus corticosteroids (Ruxolitinib Group) 32](#_Toc132729913)

[5.1.2. Corticosteroids (Corticosteroids Group) 33](#_Toc132729914)

[5.2. Criteria for Permanent Discontinuation of Study Drug 34](#_Toc132729915)

[5.3. Treatment Compliance 34](#_Toc132729916)

[5.4. Withdrawal of Subjects from Study Treatment 35](#_Toc132729917)

[5.4.1. Withdrawal Criteria 35](#_Toc132729918)

[5.4.2. Withdrawal Procedures 36](#_Toc132729919)

[5.4.3. Study Completion 37](#_Toc132729920)

[6. STUDY ASSESSMENTS 38](#_Toc132729921)

[6.1. Screening 38](#_Toc132729922)

[6.2. Treatment 38](#_Toc132729923)

[6.3. End of Treatment 39](#_Toc132729924)

[6.4. Re-Treatment 39](#_Toc132729925)

[6.5. Follow-Up 40](#_Toc132729926)

[6.5.1. Safety Follow-Up 40](#_Toc132729927)

[6.5.2. Post-Treatment GVHD Follow-Up 40](#_Toc132729928)

[6.5.3. Survival Follow-Up 40](#_Toc132729929)

[6.6. Unscheduled Visits 41](#_Toc132729930)

[7. CONDUCT OF STUDY ASSESSMENTS AND PROCEDURES 42](#_Toc132729931)

[7.1. Administration of Informed Consent Form 42](#_Toc132729932)

[7.2. Demography and Medical History 42](#_Toc132729933)

[7.2.1. Demographics and General Medical History 42](#_Toc132729934)

[7.2.2. Disease Characteristics and Treatment History 42](#_Toc132729935)

[7.3. Prior and Concomitant Medications 43](#_Toc132729936)

[7.4. Safety Assessments 43](#_Toc132729937)

[7.4.1. Adverse Events 43](#_Toc132729938)

[7.4.2. Serious Adverse Events 48](#_Toc132729939)

[7.4.3. Physical Examination 50](#_Toc132729940)

[7.4.4. Vital Signs 51](#_Toc132729941)

[7.4.5. ECOG Performance Status 51](#_Toc132729942)

[7.4.6. Laboratory Assessments 52](#_Toc132729943)

[7.5. Efficacy Assessments 53](#_Toc132729944)

[7.5.1. GVHD Staging and Grading 54](#_Toc132729945)

[7.5.2. Chronic GVHD Assessment 57](#_Toc132729946)

[7.5.3. Graft Failure and Donor Chimerism 57](#_Toc132729947)

[7.5.4. Post-Transplant Lymphoproliferative Disorder Assessment 57](#_Toc132729948)

[7.5.5. Relapse/Recurrence of Underlying Hematologic Disease 58](#_Toc132729949)

[7.6. Blood Sample Collection 58](#_Toc132729950)

[8. STATISTICS 59](#_Toc132729951)

[8.1. Selection of Sample Size 59](#_Toc132729952)

[8.2. Level of Significance 59](#_Toc132729953)

[8.3. Statistical Analyses 59](#_Toc132729954)

[8.3.1. Primary Analyses 59](#_Toc132729955)

[8.3.2. Secondary Analyses 60](#_Toc132729956)

[8.3.3. Safety Analyses 61](#_Toc132729957)

[8.4. Handling of missing data 61](#_Toc132729958)

[8.5. Analyses for the Data Monitoring Committee 61](#_Toc132729959)

[9. ETHICAL APPROVAL 62](#_Toc132729960)

[10. REFERENCES.......................................................................................................62](#_Toc132729960)

[APPENDIX A. Table of Assessments 67](#_Toc132729961)

[Table 1: Schedule of Assessments 67](#_Toc132729962)

[APPENDIX B. PROTOCOL AMENDMENT SUMMARY OF CHANGES 69](#_Toc132729963)

[Amendment (Version) 2 (23 May 2022) 69](#_Toc132729964)

# LIST OF ABBREVIATIONS

The following abbreviations and special terms are used in this clinical study Protocol.

| **Abbreviation** | **Definition** |
| --- | --- |
| AE | adverse event |
| acute GVHD | acute graft-versus-host disease |
| allo-HSCT | allogeneic hematopoietic stem cell transplantation |
| ALP | alkaline phosphatase |
| ALT | alanine aminotransferase |
| ANC | absolute neutrophil count |
| AST | aspartate aminotransferase |
| BMT | Bone Marrow Transplant |
| CI | confidence interval |
| CFR | Code of Federal Regulations |
| cGVHD | chronic graft-versus-host disease |
| CIBMTR | Center for International Blood and Marrow Transplant Research |
| CMV | cytomegalovirus |
| CR | complete response |
| CSA | cyclosporine A |
| CTCAE | Common Terminology Criteria for Adverse Events |
| DFS | disease-free survival |
| DLT | dose-limiting toxicity |
| DMC | Data Monitoring Committee |
| DNA | deoxyribonucleic acid |
| DOR | duration of response |
| EBV | Epstein–Barr virus |
| ECOG | Eastern Cooperative Oncology Group |
| eCRF | electronic case report form |
| EOT | end of treatment |
| **Abbreviation** | **Definition** |
| FDA | Food and Drug Administration |
| FFS | failure-free survival |
| GCP | Good Clinical Practice |
| GI | gastrointestinal |
| GVH | graft versus host |
| GVHD | graft-versus-host disease |
| GVT | graft versus tumor |
| HBsAg | hepatitis B surface antigen |
| HBV | hepatitis B virus |
| HCV | hepatitis C virus |
| HLA | human leukocyte antigen |
| HSCT | hematopoietic stem cell transplant |
| ICF | informed consent form |
| IFN-γ | interferon-γ |
| IFN-γR | interferon-γ receptor |
| IL | interleukin |
| IL-2R | interleukin 2 receptor |
| IQR | interquartile range |
| JAK | Janus kinase |
| LLN | lower limit of normal |
| MAGIC | Mount Sinai Acute GVHD International Consortium |
| MAP | MAGIC algorithm probability |
| NIH | National Institutes of Health |
| NK | natural killer |
| NRM | nonrelapse mortality |
| ORR | overall response rate |
| OS | overall survival |
| PCR | polymerase chain reaction |
| **Abbreviation** | **Definition** |
| PO | orally |
| PR | partial response |
| PTLD | post-transplant lymphoproliferative disorder |
| QD | once daily |
| REG3α | regenerating islet-derived 3-alpha |
| RNA | ribonucleic acid |
| SAE | serious adverse event |
| SR | steroid-refractory |
| sST2 | soluble suppression of tumorigenesis-2 |
| STAT | signal transducers and activators of transcription |
| sSTNFR1 | soluble tumor necrosis factor receptor 1 |
| TNF | tumor necrosis factor |
| Treg | regulatory T cell |
| TTR | time to response |
| TYK | tyrosine kinase |

# 1. INTRODUCTION

## 1.1. Overview of Acute Graft-Versus-Host Disease

Acute graft-versus-host disease (acute GVHD) remains a major transplantation-related complication despite standard prophylaxis ^[1-31]^. Systemic corticosteroid therapy is the first-line treatment for newly diagnosed acute GVHD patients ^[4, 5]^. However, steroid-refractory acute GVHD (SR-acute GVHD) occurs in approximately 35–50% of patients and is associated with high mortality ^[1, 6-8]^, indicating an intrinsic variability of glucocorticoid sensitivity at acute GVHD onset ^[9]^. The steroid resistance in recurrent acute GVHD further supports this speculation ^[4]^. Enhancing glucocorticoid sensitivity in newly diagnosed acute GVHD patients for the first-line therapy could result in therapeutic benefit. Methylpredinisone at a dose of 2 mg/kg also renders patients susceptible to infections, osteoporosis, and various metabolic disturbances ^[8, 10]^. Thus, there is an urgent requirement for novel active first-line treatments to improve efficiency and decrease the side effects in newly diagnosed acute GVHD patients.

The pathogenesis of acute GVHD is complicated. Early tissue injuries, the activation of innate immune cells and donor T cells, and the subsequent immune response lead to healthy tissue damage ^[11, 12]^. Janus kinases (JAKs) are intracellular signaling molecules that regulate the activities of immune cells, including neutrophil cells, antigen-presenting cells, T cells, and B cells underlying GVHD, and therefore regulate acute GVHD pathogenesis ^[11, 13]^. Cytokines and chemokines such as interleukin (IL) -1, interferon-gamma, IL-2, tumor necrosis factor, IL-6, IL-17, and IL-33 are involved in GVHD pathogenesis ^[5, 11, 14]^. Intracellular signaling downstream of multiple cytokines related to acute GVHD is partialy transduced by JAK signaling pathways ^[15]^.

## 1.2. MAGIC Algorithm Probability

The clinical stage of acute GVHD is defined according to each involved organ’s clinical assessment of symptoms. The organ stages are totaled in an overall grade (I–IV) ^[16]^. Grade III/IV acute GVHD is associated with high mortality rate (50%–70%) and the possibility of steroid resistance. Serum biomarkers have also emerged as an additional potential measurement of acute GVHD severity ^[17, 18]^. The Mount Sinai Acute GVHD International Consortium (MAGIC) is a group of 25 stem cell transplantation centers conducting GVHD research. It has established an algorithm that combines two biomarkers, namely ST2 and REG3α. The MAGIC algorithm probability (MAP) predicts the response to first-line treatment of corticosteroids and 6-month non-relapse mortality (NRM). When measured at acute GVHD onset, the MAP could categorize the patients into three risk groups, each group with significantly different risk of NRM. The proportion of patients resistant to treatment at week 4 was higher in the high MAP risk group than in the low-risk group (67% vs. 30%, p = 0.03) ^[14, 19]^. Therefore, novel and active first-line therapies are urgently required in patients with high-risk acute GVHD prone to steroid resistance ^[9, 20]^.

## 1.3. Ruxolitinib Background and Study Rationale

Ruxolitinib, a selective inhibitor of Janus kinase (JAK) 1/2, is the first Food and Drug Administration-approved medication for SR-acute GVHD ^[4, 15, 21]^. The REACH2 phase 3 randomized trial showed that in patients with SR-acute GVHD, the overall complete response (CR) at day 28 was higher in the ruxolitinib group than the control group (62% vs. 39%; p < 0.001). The durable overall response rate (ORR) at day 56 was significantly higher in the ruxolitinib group than that in the control group (40% vs 22%; p < 0.001) ^[22]^. The incidence of infection was similar in ruxolitinib and control therapy, with grade 3 severity of 22% and 19%, respectively. The incidence of cytomegalovirus (CMV) reactivation was 26%, and Epstein-Barr virus (EBV) reactivation was 6% in the ruxolitinib group ^[22]^. Delgado MC et al. showed that ruxolitinib enhances cell sensitivity to dexamethasone-induced apoptosis in vitro. The combination of corticosteroid and ruxolitinib alteres the balance between pro- and anti-apoptotic factors in cells with corticosteroid resistance ^[23]^. Thus, the addition of ruxolitinib may improve the efficacy of corticosteroids in SR-acute GVHD patients. Treatment with methylprednisolone at 2 mg/kg/day or prednisone at 2.0 to 2.5 mg/kg/day is the standard first-line systemic therapy for acute GVHD. Methylprednisolone is the most commonly used corticosteroid. Also, other types of steroids are available if administered at an equivalent steroid dose. In the present study, methylprednisolone was adopted to standardize the research.

We previously reported the results of the first-line treatment for newly diagnosed acute GVHD patients with a combination of different doses of ruxolitinib with corticosteroid ^[4, 24]^. This phase I dose-finding study investigated the optimal dose of ruxolitinib combined with corticosteroid (ClinicalTrials.gov Identifier: NCT04397367). The average accumulated dose of methylprednisolone was 17.6 mg/kg (standard deviation [SD] = 8.2) with a mean withdrawal time of 42.6 ± 16.8SD days. Patients were treated with three different doses of ruxolitinib: 10 mg twice daily for the first three patients, 5 mg twice daily for the following 12 patients, and 5 mg once daily for the remaining 17 patients. In the first three patients, intolerable hematologic toxicity related to 10 mg twice daily ruxolitinib was observed. CMV and EBV diseases were seen in 2 of 12 patients who received a dose of ruxolitinib 5 mg twice daily, with one suffering from CMV infection and another developing post-transplant lymphoproliferative disorder. As observed in the last 17 patients, 5 mg/day Ruxolitinib combined with methylprednisolone (1 mg/kg/day) was well tolerated with decreased CMV reactivation and promising signals of efficacy as demonstrated by 28-days ORR of 82.05% ^[24]^. After initiation of the novel first-line therapy, remission of acute GVHD in all patients occurred at a median time of 3.2 (interquartile range [IQR], 1–7) days. Of all patients, 10 patients (31.2%) developed recurrent acute GVHD after complete remission. The causes of recurrent GVHD included ruxolitinib and cyclosporine reduction (n=8) and therapeutic donor lymphocyte infusion (DLI; n=1). The relapse rate of primary disease in all patients with acute GVHD was 15.6% (5/32). With a median follow-up of 260 days, the 1-year overall survival (OS) and disease-free survival (DFS) were 73.4% (CI: 56.6%–95.1%) and 61.2% (95% CI 37.4%–95.6%), respectively. Cyclosporine A (CSA) is tapered over 60 days in the Ruxolitinib group with durable complete response. Short duration of CSA may accelerate immune reconstitution, which is good for reducing relapse of primary disease and infection. These data suggested that a regimen of ruxolitinib plus corticosteroids may improve the long-term outcomes of patients with newly diagnosed acute GVHD with tolerance.

## 1.4. Potential Risks and Benefits of the Treatment Regimen

Adverse events that have been most frequently reported in at least 10% of subjects receiving ruxolitinib monotherapy include anemia, thrombocytopenia, diarrhea, nausea, fatigue, and upper respiratory tract infection.

As a result of ruxolitinib-mediated immunomodulation, an increased incidence of infections could possibly occur with ruxolitinib therapy. Strict clinical monitoring is indicated to identify and treat infections in study subjects should they occur.

Because of the potential for myelosuppression, subjects will have hematologic parameters closely monitored during clinical studies. If there are clinically relevant declines in hematology parameters, therapy may be interrupted until resolution or discontinuation. As ruxolitinib also has the potential to cause WBC margination (ie, a transient decrease in ANC), assessment of hematology parameters should be performed before study drug administration and at all applicable study visits.

As described in 1.3., ruxolitinib in previous clinical studies shows the capability of GVHD reduction, preservation of the beneficial GVT effect, and improvement in survival.

# 2. STUDY OBJECTIVES AND ENDPOINTS

| Primary Objective | Primary Endpoint |
| --- | --- |
| Compare the efficacy of ruxolitinib in combination with 1mg/kg methylprednisolone versus 2 mg/kg methylprednisolone alone in terms of overall response rate (ORR) at Day 28 in subjects with acute graft-versus-host disease (acute GVHD). | ORR at Day28, defined as the proportion of participants demonstrating a complete response (CR), or partial response (PR) |
| Secondary Objective | Secondary Endpoints |
| Compare additional response and longer-term efficacy outcomes between treatment cohorts. | Durable ORR at Day56, defined as the proportion of patients in each arm whose response on day 28 lasts until day 56 after randomization. |
|  | DOR at Month 6 for responders will be calculated. The DOR is defined from the time of the onset of response to loss of response. Subjects who died or discontinued will be censored at the  death date or the previous assessment. |
|  | Response duration, defined as the time from the first response to the first recurrence/progression of acute GVHD or additional immunosuppressants for GVHD. |
|  | NRM at Month 24, defined as the time from enrollment to death due to any causes other than hematologic disease relapse. |
|  | Cumulative incidence of relapse (CIR) at Month 24, defined as the proportion of subjects whose underlying hematologic disease relapses. |
|  | Disease-free survival (DFS) at Month 24, defined as the time from enrollment to relapse of primary disease or death from any causes, whichever occurred first. |
|  | Failure-free survival (FFS) at Month 24, defined as the time from randomization to disease relapse or progression, non-relapse mortality, or the addition of new therapy for acute GVHD. |
|  | Overall survival (OS) at Month 24, defined as the time from randomization to death due to any causes. |
| Assess the incidence and severity of adverse events (AEs) and serious adverse events. | Clinical safety data (eg, AEs, infections) will be tabulated and listed. |
| Evaluate the use and discontinuation of  corticosteroids. | Average and cumulative corticosteroid dose at Days 365; proportion of subjects who discontinue corticosteroids at Days 56 and 100. |
| Evaluate the use and discontinuation of  immunosuppressive medications. | Proportion of subjects who discontinue immunosuppressive medications at Days 365. |
| Evaluate the incidence of recurrent acute GVHD. | Incidence rate of recurrent acute GVHD through Month 12. |
| Evaluate the incidence of cGVHD. | Incidence rate of cGVHD at Month 24. |

# 3. SUBJECT ELIGIBILITY

Eligible patients with acute GVHD graded according to the modified Glucksberg criteria and untreated with systemic acute GVHD therapy will be enrolled in this study. Grade of acute GVHD response to treatment will be determined by a special team of three experienced doctors from the transplantation centers. All centers are experienced in the management of GVHD and stem cell transplantation. However, some conditions, such as poor patient compliance to doctor’s orders, will interfere with patients’ participation in the study. Such patients should not be included in the study at the judgment of the research team. Throughout the study period, the directors of the centers would supervise the implementation and review of the research documents. Patients who fulfill all inclusion criteria could be included in the study.

## 3.1. Subject Inclusion Criteria

A subject who meets all of the following criteria may be included in the study:

1. Male or female, 14 to 65 years old.

2. Have undergone first allogeneic hematopoietic stem cell transplantation (allo-HSCT) from any donor source using bone marrow, peripheral blood stem cells, or cord blood for hematologic malignancies.

3. Develop a de novo acute GVHD, defined as intermediate- or high-risk using the Minnesota acute GVHD risk score and MAGIC biomarker algorithm.

## 3.2. Subject Exclusion Criteria

1. Has received more than one allogeneic stem cell transplant.

2. Female patients with pregnant or breast-feeding.

3. Has Acquired Immune Deficiency Syndrome (AIDS), or hepatitis B/C infection, uncontrolled bacterial, fungal, or viral infections.

4. Allergies to ruxolitinib.

5. Vital organ dysfunction unrelated to GVHD, including persistent bilirubin abnormalities or severe heart and respiratory diseases.

6. Relapse of the primary disease or graft rejection after transplantation.

7. Presence of GVHD overlap syndrome.

8. Presence of cGVHD or associated clinical signs or DLI-induced acute GVHD or interferon.

9. Previous history of JAK1/2 inhibitor treatment after transplantation, except JAK inhibitors administered before the transplantation.

10. absolute neutrophil count (ANC) <0.5×10^9^/L or platelet count (PLT) < 20×10^9^/L

11. Serum creatinine > 2.0 mg/dL or creatinine clearance < 40 mL/min measured or calculated by Cockroft-Gault equation.

12. Patients with poor compliance should not be included in the study based on the judgment of the research team, for example if they refused to take medicine at will many times during the previous treatment.

## 3.3. Lifestyle Considerations

### 3.3.1. Meals and Dietary Restrictions

Subjects should be instructed to refrain from the consumption of pomegranates or pomegranate juice and grapefruit or grapefruit juice, as these are known to inhibit cytochrome CYP3A enzymes and may increase the exposure to ruxolitinib.

### 3.3.2. Activity

No restrictions are required.

# 4. INVESTIGATIONAL PLAN

## 4.1. Overall Study Design

This trial is an open-label, multicenter, prospective randomized two-arm phase II study comparing the efficacy and safety of ruxolitinib plus methylprednisolone (1 mg/kg) vs. methylprednisolone (2 mg/kg) alone (Figure 1). The planned sample size is 198 patients for internet-based randomization. The biomarkers of acute GVHD, such as sST2, sTNFR1, IL-6, IL-8, and REG3α will be tested at acute GVHD onset. Patients with newly diagnosed intermediate-risk or high-risk acute GVHD, as defined by Minnesota acute GVHD risk score and the biomarker score will be included. The treatment efficacy will be evaluated with respect to the level of biomarkers and clinical manifestations. Written informed consent must be obtained from the patients before enrollment in the study. The consent form and related materials are available from the corresponding author on request.

## 4.2. Recruitment procedures

The participants will be recruited in one of the transplantation centers located in Beijing, Dalian, Shijiazhuang, and Jinan, respectively. All these centers are highly experienced in the management of GVHD and stem cell transplantation. Throughout the study period, the directors of the centers supervise the implementation and review of the research documents. An independent research assistant recruits the eligible participants. In each transplant center, the doctors participating in this study are trained uniformly for treatment, taking informed consent, and study visits. The trained nurses coordinate study visits and record data. Each center will recruit patients (enrollment from August 2019 to October 2022, five patients from all centers every month). No financial incentives were provided to trial investigators or participants for enrolment.

**Table 1: List of participating study centers**

| **Study site** | **Principal investigator** |
| --- | --- |
| Chinese PLA General Hospital, the First Medical Center | Daihong Liu |
| Beijing Lu Daopei Hospital | Peihua Lu, Yanli Zhao, Zhijie Wei |
| the 960 Hospital of the PLA Joint Logistics Support Force | Fang Zhou |
| The Second Hospital of Hebei Medical University | Fuxu Wang |
| The Second Hospital of Dalian Medical University | Yan Yang, Jinsong Yan |
| Chinese PLA General Hospital, the Fifth Medical Center | Liangding Hu |
| Qilu Hospital, Shandong University | Chuanfang Liu |

The transplantation knowledge was provided to the patients and their relatives in order to emphasize the management of transplantation and promote participant retention. Also, a standardized follow-up booklet is granted to each enrolled patient for recording and reminding the detailed follow-up items and timepoints. An in-time communication between investigators and subjects by mobile phones will facilitate the completion of follow-up.

## 4.3. GVHD biomarker Luminex assays

Peripheral blood samples are collected at acute GVHD onset ^[17, 28, 29]^. Designated personnel are responsible for collecting and transporting these blood samples that are shipped to Bofurui Biolab for MAP analyses for ST2 and REG3a by flow cytometry. Luminex Assay Human Premixed Multi-Analyte Kits (R&D, MN, USA, Catalog No. LXSAHM-05) is used for measurement of ST2 and REG3a, according to the manufacturer’s protocol. Samples (diluted as 1:2) and standards are run in duplicate, the absorbance is measured (Luminex 200), the data are estimated using versionXponent_4.2 (Luminex 200), and MAP is calculated accordingly.

## 4.4. Randomization

The eligibility of patients is determined by study site staff. Then, the participants who meet the enrollment criteria will be randomized to receive one of the two treatments in a 1:1 ratio and stratified by disease status before transplantation (complete remission vs. non- complete-remission) and risk of acute GVHD (intermediate-risk vs. high-risk). Stratified permuted block randomization lists will be used to generate the stratified randomization. Randomization is implemented through an interactive web-based response system independent of study site staff and investigators. The codes are generated independently of the study by a statistician. The next assignment in the random sequence remained concealed because the treatment was assigned remotely. Treatment allocations are not masked to investigators or participants as CSA and methylprednisolone dosage are different between the two groups. The study staff who conducted the data analysis and assessments of outcomes will be masked for treatment allocations.

## 4.5. Number of Subjects

### 4.5.1. Planned Number of Subjects

This study will enroll approximately 99 subjects with clinically or pathologically confirmed Grade II to IV acute GVHD per treatment cohort, for a total of 198 subjects at approximately 7 study sites.

### 4.5.2. Statistical Rational for the Sample Size

The sample size is calculated according to the primary endpoint (ORR) of the study. Based on our published phase I study of 32 acute GVHD patients who received steroids as first-line therapy, an expected proportion of 55% for the patients treated with corticosteroids only was established ^[4]^. Based on our published data of patients with acute GVHD grade I–IV who received steroid-ruxolitinib (5 mg/day) as first-line therapy, the ORR was 82.05% ^[24]^. Patients with acute GVHD grade I were also included in the phase I analysis. Thus, an expected proportion of 75% for the patients treated with steroid-ruxolitinib (5mg/day) was established. This study is planned to detect a response difference between treatment arms at a two-sided significance level α=5% with a power of 1-β=80%. The sample size was estimated using PASS software based on the primary endpoint (ORR). Allowing a withdrawal rate of 10%, 198 patients (99/group) will be required.

### 4.5.3. Replacement of Subjects

Not applicable.

## 4.6. Duration of Treatment and Subject Participation

After signing the informed consent form (ICF), screening assessments may be completed over 1-2 days. Each subject enrolled in the study may continue to receive study treatment as long as benefit is being observed and/or treatment withdrawal criteria are not met. If the subject discontinues study treatment, the treatment period will end, and the subject will enter the follow-up period. The safety follow-up period will last 365 days, and the survival follow-up period will last until death or study withdrawal. Study participation is expected to average approximately 24 months per individual subject but may vary based on clinical outcomes.

## 4.7. Overall Study Duration

The study begins when the first subject signs the informed consent. Subjects who are still on-study at the time of the primary endpoint analysis will continue to receive study treatment until treatment withdrawal criteria are met. All subjects will be followed for survival until death, withdrawal of consent, or the end of the study, whichever occurs first. The study will end once 75% of subjects have died or are lost to follow-up. Provisions will be made to ensure access to treatment for subjects who are continuing to benefit from study treatment at the time of study completion.

## 4.8. Study Termination

The investigator retains the right to terminate study participation at any time, according to the terms specified in the study contract. The investigator is to notify the independent ethics committee (IEC) in writing of the study's completion or early termination, send a copy of the notification to the sponsor or sponsor's designee, and retain 1 copy for the site study regulatory file.

# 5. TREATMENT

## 5.1. Study Drugs

### 5.1.1. Ruxolitinib plus corticosteroids (Ruxolitinib Group)

**5.1.1.1. Administration and Tapering**

The initial dose of methylprednisolone is 1 mg/kg/day intravenously given in this arm for at least 7 days. The dosage of ruxolitinib is 5 mg PO QD. CSA will be administered intravenously at a dose of 2 mg/kg twice daily, targeting minimum concentration levels of 150–250 ng/mL. If GVHD response to treatment is assessed as PR/CR at 7 days, methylprednisolone will be tapered, followed by reduction and discontinuation of CSA and then ruxolitinib. The reduction of the methylprednisolone in the ruxolitinib group is gradual, suggesting it be tapered off over 6 weeks as follows: dosage reduced to 0.6 mg/kg/day, 0.4 mg/kg/day, 0.3 mg/kg/day, 0.25 mg/kg/day, and 0.18 mg/kg every 5 days, followed by 0.1 mg/kg/day at week 4, 0.1 mg/kg every other day after 5 days and stopped at week 6. After steroid discontinuation and CR or stable PR maintained for 6 weeks, CSA is tapered over 60 days. After CSA discontinuation and no presence of recurrent GVHD, ruxolitinib is tapered off over 90 days, which is totally maintained for approximately 6 months ^[2]^.

In view of the durable response and safety observed in previous ruxolitinib studies, the clinical benefits of ruxolitinib outweigh the risks of treatment. Retrospective data indicated that prolonged maintenance of ruxolitinib is allowed even after refractory GVHD and responses in acute GVHD can be observed at 11 weeks after ruxolitinib treatment ^[4, 15]^. Prolonged maintenance of ruxolitinib is allowed in the case of recurrent acute GVHD or refractory acute GVHD (the progression of GVHD after 3 days of therapy, no improvement within 7 days, or no CR after 14 days of therapy) after ruxolitinib plus corticosteroid treatment ^[4]^. If patients in the ruxolitinib group do not reach the CR by day 28, ruxolitinib will also be continued beyond 6 months in the event of no contraindications. Tapering of ruxolitinib is permitted after day 56 in patients who have a response to the second line therapy for recurrent acute GVHD or refractory acute GVHD. An additional follow‐up through at least day 180 is required to determine the durability of treatment responses ^[27]^. During the trial, drug interactions should be examined first before adding new agents in order to reduce the potential effects of other drugs on ruxolitinib. Fluconazole >200 mg/day is prohibited as it disrupts the metabolism of ruxolitinib.

### 5.1.2. Corticosteroids (Corticosteroids Group)

**5.1.2.1. Administration and Tapering**

The initial dose of methylprednisolone is 2 mg/kg/day given twice daily for at least 7 days and then reduced ^[8]^. The dosage of methylprednisolone in the corticosteroids group is decreased gradually after CR and tapered off over 10 weeks as the following: dosage reduced to 1 mg/kg/day to 0.6 mg/kg/day, 0.4 mg/kg/day, 0.3 mg/kg/day, 0.25 mg/kg/day, and 0.18 mg/kg every 7 days, and then 0.1 mg/kg/day at week 4, 0.1 mg/kg every other day after 5 days, and stopped at week 10. CsA was administered intravenously at a dose of 2 mg/kg twice daily, targeting minimum concentration levels of 150–250 ng/mL. The recommended duration of CsA is 6 months.

**5.1.2.2. Criteria and Procedures for Dose Interruptions or Adjustments of** **Corticosteroids**

## The dosage of methylprednisolone in the corticosteroids group is decreased gradually after CR and tapered off over 10 weeks as the following: dosage reduced to 1 mg/kg/day to 0.6 mg/kg/day, 0.4 mg/kg/day, 0.3 mg/kg/day, 0.25 mg/kg/day, and 0.18 mg/kg every 7 days, and then 0.1 mg/kg/day at week 4, 0.1 mg/kg every other day after 5 days, and stopped at week 10.

## 5.2. Criteria for Permanent Discontinuation of Study Drug

The occurrence of unacceptable toxicity not caused by the underlying disease or malignancy will be presumed to be related to study drug treatment and will require that the study drug be permanently discontinued. Unacceptable toxicity is defined as follows:

- Occurrence of an AE that is related to treatment with the study drug that, in the

judgment of the investigator or the sponsor's medical monitor, compromises the

subject's ability to continue study-specific procedures or is considered not to be in the

subject's best interest.

- Persistent AE requiring a delay of therapy for more than 14 days, unless a greater

delay has been approved by the sponsor.

## 5.3. Treatment Compliance

Treatment compliance with all study-related medications should be emphasized to the subject by the site personnel, and appropriate steps should be taken to optimize compliance during the study. Ruxolitinib compliance will be calculated, by the sponsor, based on the drug accountability documented by the site staff and monitored by the sponsor/designee (tablet counts). Subjects will be instructed to bring all study-related medications with them to each study visit in order for site personnel to conduct tablet counts to assess study drug accountability. The drug accountability documentation will be used by the sponsor to calculate treatment compliance. Although commercial supplies of corticosteroids will be used, dose changes and interruptions will also be documented in the medical record and monitored by the sponsor or its designee. As corticosteroid dose strengths and administration types will vary, compliance with corticosteroids will not be calculated.

## 5.4. Withdrawal of Subjects from Study Treatment

The decision to discontinue study treatment will not constitute study completion. In the event that the decision is made to discontinue study treatment, the treatment period will be considered complete, and the follow-up period will begin.

### 5.4.1. Withdrawal Criteria

Subjects must be withdrawn from study treatment for the following reasons:

- The subject has experienced an unacceptable toxicity.
- Relapse of underlying malignancy.
- The subject is unable to tolerate ruxolitinib at a dose of 5 mg QD.
- Additional systemic therapy is required for GVHD progression or lack of response, including corticosteroid doses greater than those used on Study Day 1.
- Further participation would be injurious to the subject's health or well-being, in the investigator's medical judgment.
- The subject becomes pregnant.
- Consent is withdrawn.
- The study is terminated by the sponsor.

A subject **may** be withdrawn from study treatment as follows:

- If, during the course of the study, a subject is found not to have met eligibility criteria, then the medical monitor, in collaboration with the investigator, will determine whether the subject should be withdrawn from the study.
- If a subject is noncompliant with study procedures or study drug administration in the investigator's opinion, the sponsor should be consulted for instruction on handling the subject.

### 5.4.2. Withdrawal Procedures

In the event that the decision is made to permanently discontinue the study drug, the subject will be withdrawn from the study and the EOT visit should be conducted. Reasonable efforts should be made to have the subject return for a follow-up visit. The last date of the last dose of study drug and the reason for subject withdrawal will be recorded in the eCRF.

**If a subject is withdrawn from study treatment:**

- The study monitor or sponsor must be notified.
- The reason(s) for withdrawal must be documented in the subject's medical record and in the eCRF.
- The EOT visit should be performed.
- The date of the EOT visit should be registered in the IVRS.
- Subjects must be followed for safety until the time of the follow-up visit or until study drug–related toxicities resolve, return to baseline, or are deemed irreversible, whichever is longest.

If the subject discontinues study treatment and actively withdraws consent for collection of follow-up data (safety follow-up or disease assessment), then no additional data collection should occur; however, subjects will have the option of withdrawing consent for study treatment but continuing in the follow-up period of the study for safety/efficacy assessments.

### 5.4.3. Study Completion

A subject will be considered as completing the study if they meet any of the following criteria:

- Subject dies and a date of death is available.
- Subject is known to have died; however, the date of death cannot be obtained. (NOTE: Every effort must be made to obtain the date of death.)
- Subject has discontinued study treatment and has withdrawn consent for collection of follow-up anticancer and survival data.

# 6. STUDY ASSESSMENTS

## 6.1. Screening

The screening period is the interval between signing the ICF and the day the subject is

randomized in the study (Day 1). The screening period may not exceed 2 days. Informed consent must be obtained before performing any study-specific procedures that are not considered standard of care. Assessments that are required to demonstrate eligibility may be performed over the course of 1 or more days during this period.

Procedures conducted as part of the subject's routine clinical management (eg, complete blood count) and obtained before signing of informed consent may be used for screening or baseline purposes, provided that the procedure meets the Protocol-defined criteria and has been performed in the timeframe of the study. Results from the screening visit evaluations will be reviewed to confirm subject eligibility before randomization or the administration of study drug. Tests with results that fail eligibility requirements may be repeated once during the screening period if the investigator believes the results to be in error or believes there has been a change in eligibility status (eg, following recovery from an infection). For screening assessments that are repeated, the most recent available result before randomization will be used to determine subject eligibility. Treatment should start as soon as possible but within 1 days after the date of randomization.

## 6.2. Treatment

The treatment period begins on the day the subject receives the first dose of study drug through the point at which the principal investigator determines the subject will be permanently discontinued from study drug. Dates for subsequent study visits will be determined based upon this day and should occur within ± 3 days of the scheduled date unless delayed for safety reasons. During the Day 1 visit, results from screening visit evaluations should be reviewed to determine whether the subject continues to meet the eligibility requirements as specified in the Protocol.

## 6.3. End of Treatment

If a decision is made that the subject will permanently discontinue study treatment, the EOT visit should be conducted. If the EOT visit coincides with a regular study visit, the EOT evaluations will supersede those of that scheduled visit, and the data should be entered in the EOT visit in the eCRF. The subject should be encouraged to return for the follow-up visit.

## 6.4. Re-Treatment

For subjects who experience acute GVHD recurrence after taper of randomized treatment is completed, investigators have the option to use open-label ruxolitinib plus corticosteroids for the treatment of recurrent acute GVHD irrespective of initial randomized treatment. For the purpose of this Protocol, treatment of recurrent acute GVHD with open-label ruxolitinib plus corticosteroids will be called the re-treatment phase. Subjects entering the re-treatment phase will be required to follow the assessment schedule. Corticosteroid tapering will be performed

at the investigator's discretion. Investigators wishing to initiate a taper of ruxolitinib earlier than Day 180 may do so upon consultation with and approval from the sponsor's medical monitor. Subjects may be re-treated with ruxolitinib only once. Subjects ending the re-treatment phase will repeat the EOT visit and subsequent safety and survival follow-up visits.

## 6.5. Follow-Up

### 6.5.1. Safety Follow-Up

The safety follow-up period is the interval between the EOT visit and the scheduled follow-up visit, which should occur 180 days after the EOT visit (or after the last dose of study drug if the EOT visit was not performed). Adverse events and SAEs must be reported up until at least 30 days after the last dose of study drug, the date of the follow-up visit, or until toxicities resolve, return to baseline, or are deemed irreversible, whichever is longer.

If a subject withdrew from treatment due to reasons other than disease progression, GVHD staging and grading will be repeated at the safety follow-up visit.

### 6.5.2. Post-Treatment GVHD Follow-Up

Subjects who completed study treatment or discontinued treatment for reasons other than GVHD progression will be followed every 28 days (± 7 days) after the safety follow-up visit until any of the following occurs:

- GVHD progression.
- Initiation of a new anti-GVHD therapy.
- Relapse/recurrence of underlying hematologic disease.
- A maximum of 12 months from EOT or a maximum of 24 months from Day 1 is

reached, whichever occurs first.

### 6.5.3. Survival Follow-Up

Subjects who complete post-treatment follow-up or experience GVHD progression or require a new anti-GVHD therapy should be contacted by telephone, email, or visit at least every 8 weeks (± 7 days) to assess for new GVHD therapy and survival status until death, withdrawal of consent, or the end of the study, whichever occurs first.

## 6.6. Unscheduled Visits

Unscheduled visits may be held at any time at the investigator's discretion, and appropriate clinical and laboratory measurements performed based on AEs or other findings.

# 7. CONDUCT OF STUDY ASSESSMENTS AND PROCEDURES

## 7.1. Administration of Informed Consent Form

Valid informed consent must be obtained from the study subject before conducting any study-specific procedures using an ICF approved by the local ICH/IEC that contains all elements required by ICH E6 and describes the nature, scope, and possible consequences of the study in a form understandable to the study subject. Local and institutional guidelines for ICF content and administration must be followed; the original signed ICF must be retained by the investigator, and a copy of the signed ICF must be provided to the study subject. The informed consent process for each subject must be documented in writing within the subject source documentation. Subjects of childbearing potential must agree to take appropriate measures to avoid pregnancy in order to participate in the study.

## 7.2. Demography and Medical History

### 7.2.1. Demographics and General Medical History

Demographic data and a complete medical and medication history will be collected at screening.

### 7.2.2. Disease Characteristics and Treatment History

A disease-targeted medical and medication history including hematologic malignancy type, current GVHD staging, date of diagnosis, sites of disease, prior anticancer therapy, ablation therapy, prophylaxis therapy, donor type, and other details related to the disease under study will be collected at screening.

## 7.3. Prior and Concomitant Medications

Prior and concomitant medications will be reviewed to determine subject eligibility. All concomitant medications and measures must be recorded in the eCRF, and any medication received or procedure performed within 30 days before randomization and up to the safety follow-up visit will be recorded in the eCRF. The medication record will be maintained after signing the ICF to document concomitant medications, including any changes to the dose or regimen. Concomitant medications include any prescription, over-the-counter, or natural/herbal preparations taken or administered during the study period. Concomitant treatments and/or procedures that are required to manage a subject's medical condition during the study will also be recorded in the eCRF.

## 7.4. Safety Assessments

Assessments will be performed at three stages: 1) prior-treatment screening to assess the inclusion and exclusion criteria (once from days -7 to -1 before medication); 2) treatment evaluation to determine the response and adjustment of medication maintenance (twice weekly for week 1, once weekly from week 2 to 6, once every 2 weeks from week 7 to 12, and once every month from week 13 to 24 after the start of medication n); 3) post-treatment evaluation for follow-up (every 2 months for 6 months).

### 7.4.1. Adverse Events

Adverse events (AE) will be monitored from the time the subject signs the ICF. Subjects will be instructed to report all AEs during the study and will be assessed for the occurrence of AEs throughout the study. In order to avoid bias in eliciting AEs, subjects will be asked general, nonleading questions such as "How are you feeling?" All AEs (serious and nonserious) must be recorded on the source documents and eCRFs regardless of the assumption of a causal relationship with the study drug.

**7.4.1.1. Definitions**

For the purposes of this Protocol, an AE is defined as any untoward medical occurrence associated with the use of a drug in humans, whether or not considered drug related that occurs after a subject provides informed consent. Abnormal laboratory values or test results occurring after informed consent constitute AEs only if they induce clinical signs or symptoms, are considered clinically meaningful, require therapy (eg, hematologic abnormality that requires transfusion), or require changes in the study drug(s).

**7.4.1.2. Reporting**

Adverse events that begin or worsen after informed consent should be recorded on the Adverse Events form of the eCRF. Conditions that were already present at the time of informed consent should be recorded on the Medical History form in the eCRF. Monitoring for the occurrence of new AEs should be continued for at least 30 days after the last dose of study drug. Adverse events (including laboratory abnormalities that constitute AEs) should be described using a diagnosis whenever possible rather than by individual underlying signs and symptoms. When a clear diagnosis cannot be identified, each sign or symptom should be reported as a separate AE.

The term “disease progression” should be recorded as an AE/SAE itself only if there are no other identifiable AEs/SAEs associated with the disease progression at the time of reporting. For events associated with disease progression, the relevant signs and symptom should be reported using a diagnosis whenever possible, rather than the individual underlying signs and symptoms. When a clear diagnosis cannot be identified, each sign or symptom should be reported as a separated AE. If the events

resulting from disease progression meet the criteria for an SAE (eg, resulted in hospitalization, a life-threatening event, or death), the specific event(s) should be reported as an SAE(s). In both cases (ie, AEs or SAEs related to disease progression), for each event it should be indicated whether the event (diagnosis or signs and symptoms) is related to disease progression.

The severity of AEs will be assessed using CTCAE v4.0 Grades 1 through 5. If an event is not classified by CTCAE, the severity of the AE will be graded according to the scale below to estimate the grade of severity.

| **Grade 1** | Mild; asymptomatic or mild symptoms; clinical or diagnostic observations only; intervention not indicated. |
| --- | --- |
| **Grade 2** | Moderate; minimal, local, or noninvasive intervention indicated; limiting age-appropriate activities of daily living. |
| **Grade 3** | Severe or medically significant but not immediately life-threatening; hospitalization or prolongation of hospitalization indicated; disabling; limiting self-care activities of daily living. |
| **Grade 4** | Life-threatening consequences; urgent intervention indicated. |
| **Grade 5** | Death due to AE |

The occurrence of AEs should be sought by nondirective questioning of the subject during the screening process after signing the ICF and at each visit during the study. Adverse events may also be detected when they are volunteered by the subject during the screening process or between visits, or through physical examination, laboratory test, or other assessments. To the extent possible, each AE should be evaluated to determine:

- The severity grade (CTCAE Grade 1 to 5).
- Whether there is at least a reasonable possibility that the AE is related to the study

treatment: suspected (yes) or not suspected (no).

- The start and end dates, unless unresolved at final follow-up.
- The action taken with regard to study drug.
- The event outcome (eg, not recovered/not resolved, recovered/resolved, recovering/resolving, recovered/resolved with sequelae, fatal, unknown).
- The seriousness, as per serious adverse event (SAE) definition.

Unlike routine safety assessments, SAEs are monitored continuously and have special reporting requirements. All AEs should be treated appropriately. If an AE is treated with a concomitant medication or nondrug therapy, this action should be recorded on Adverse Event form and the treatment should be specified on the Prior/Concomitant Medications or Procedures and Non-Drug Therapy form in the eCRF.

Once an AE is detected, it should be followed until it has resolved or until it is judged to be permanent; assessment should be made at each visit (or more frequently if necessary) of any changes in severity, the suspected relationship to the study drug, the interventions required to treat the event, and the outcome.

When the severity of an AE changes over time for a reporting period (eg, between visits), each change in severity will be reported as a separate AE until the event resolves. For example, 2 separate AEs will be reported if a subject has Grade 1 diarrhea, meeting the definition of an AE, that lasts for 3 days before worsening to a Grade 3 severity. The Grade 1 event will be reported as an AE with a start date equal to the day the event met the Grade 1 AE definition and a stop date equal to the day that the event increased in severity from Grade 1 to Grade 3. The Grade 3 event will also be reported as an AE, with the start date equal to the day the event changed in intensity from Grade 1 to Grade 3 and a stop date equal to the day that the event either changed severity again or resolved. For analysis purposes, this will be considered 1 AE for this subject, and the highest reported severity will be used.

**7.4.1.3. Laboratory Test Abnormalities**

Laboratory abnormalities that constitute an AE in their own right (considered clinically meaningful, induce clinical signs or symptoms, require concomitant therapy, or require changes in study drug) should be recorded on the Adverse Event form in the eCRF. Whenever possible, a diagnosis rather than a symptom should be provided (eg, "anemia" instead of "low hemoglobin"). Laboratory abnormalities that meet the criteria for AEs should be followed until they have returned to normal or an adequate explanation of the abnormality is found. When an abnormal laboratory test result corresponds to a sign or symptom of a previously reported AE, it is not necessary to separately record the laboratory test result as an additional event. Laboratory abnormalities that do not meet the definition of an AE should not be reported as AEs. A Grade 3 or 4 AE does not automatically indicate an SAE unless it meets the definition of serious, and/or per the investigator's discretion. A dose modification for the laboratory abnormality may be required and should not contribute to the designation of a laboratory test abnormality as an SAE.

### 7.4.2. Serious Adverse Events

**7.4.2.1. Definitions**

An SAE is defined as an event that meets at least 1 of the following criteria:

- Is fatal or life-threatening.
- Requires inpatient hospitalization or prolongation of existing hospitalization, unless hospitalization is a result of:
- A routine treatment or monitoring of the studied indication not associated with any deterioration in condition.
- An elective or preplanned treatment for a pre-existing condition that is unrelated to the indication under study and has not worsened since signing the ICF.
- A treatment on an emergency outpatient basis for an event not fulfilling any of the definitions of a SAE and not resulting in hospital admission.
- Any social reasons and respite care, in the absence of any deterioration in the subject's general condition.
- Results in persistent or significant disability, incapacity, or a substantial disruption of a person's ability to conduct normal life functions.
- Constitutes a congenital anomaly or birth defect.
- Is considered to be an important medical event or a medically significant event that may not result in death, be immediately life-threatening, or require hospitalization but may be considered serious when, based upon appropriate medical judgment, the event may jeopardize the subject or may require medical or surgical intervention to prevent 1 of the outcomes listed above.

**7.4.2.2. Reporting**

To ensure subject safety, every SAE, regardless of suspected causality (including events that may not be associated with the study drug[s] but may be associated with a study procedure or disease progression), unless otherwise specified by the Protocol, occurring after the subject has signed the ICF and up to the last study visit, or up to 30 days after the subject has stopped study treatment, whichever is later, must be reported to the sponsor (or designee) within 24 hours of learning of its occurrence. Any SAEs occurring more than 30 days after the last dose of study drug should be reported to the sponsor, or its designee, only if the investigator suspects a causal relationship to the study drug. An SAE occurring at a different time interval or otherwise considered completely unrelated to a previously reported SAE should be reported separately as a new event. Previously planned (ie, before providing informed consent) surgeries should not be reported as SAEs unless the underlying medical condition worsens over the course of the study.

Information about all SAEs is collected and recorded on the Adverse Event form of the eCRF. The investigator must assess and record the causal relationship of each SAE to each specific study drug (ruxolitinib and the corticosteroid during the time it is given).

The investigator must also complete the Serious Adverse Event Report Form, and send the completed and signed form to the sponsor or designee within 24 hours of becoming aware of the SAE. The investigator must provide a causality assessment, that is, assess whether there is at least a reasonable possibility that the SAE is related to the study treatment: suspected (yes) or not suspected (no).

The contact information of the sponsor's study-specific representatives is listed in the

investigator manual provided to each site. The original copy of the SAE Report Form and the confirmation sheet must be kept at the study site.

Investigational site personnel must report any new information regarding the SAE within 24 hours of becoming aware of the information in the same manner that the initial SAE Report Form was sent. Follow-up information is recorded on an amended or new SAE Report Form, with an indication that it is follow-up to the previously reported SAE and the date of the original report. The follow-up report should include information that was not provided on the previous SAE Report Form, such as the outcome of the event (eg, resolved or ongoing), treatment provided, action taken with study drug because of the SAE (eg, dose reduced, interrupted, or discontinued), or subject disposition (eg, continued or withdrew from study participation). Each recurrence, complication, or progression of the original event should be reported as follow-up to that event, regardless of when it occurs.

### 7.4.3. Physical Examination

The targeted physical examination will be a symptom-directed evaluation conducted by the investigator or a medically qualified designee. The targeted physical examination will include height (screening only) and assessment(s) of the body systems or organs, as indicated by subject symptoms, AEs, or other findings. Clinically notable abnormalities that are considered clinically significant in the judgment of the investigator are to be reported as AEs.

### 7.4.4. Vital Signs

Vital sign measurements include blood pressure, pulse, respiratory rate, body temperature, and body weight. Blood pressure and pulse will be taken with the subject in the recumbent, semirecumbent, or sitting position. Clinically notable abnormalities that are considered clinically significant in the judgment of the investigator are to be reported as AEs.

### 7.4.5. ECOG Performance Status

ECOG performance status (Table 2) will be assessed at screening and other study visits. Performance status must be assessed by a medically qualified individual and recorded in the eCRF.

**Table 2: ECOG Performance Status Grades**

| **Grade** | **Performance Status** |
| --- | --- |
| **0** | Fully active, able to carry on all predisease performance without restriction. |
| **1** | Restricted in physically strenuous activity but ambulatory and able to carry out work of a light or sedentary nature, eg, light house work, office work. |
| **2** | Ambulatory and capable of all self-care but unable to carry out any work activities. Up and about more than 50% of waking hours. |
| **3** | Capable of only limited self-care, confined to bed or chair more than 50% of waking hours. |
| **4** | Completely disabled. Cannot carry on any self-care. Totally confined to bed or chair. |
| **5** | Dead. |

### 7.4.6. Laboratory Assessments

Blood draws for laboratory assessments will occur at study visits. Blood draws will be completed before the subject receives the morning dose of study drug.

All laboratory assessments will be performed at a local (site) laboratory using institutional best practices. Results and normal reference ranges will be entered into the eCRF.

**7.4.6.1. Chemistry**

All chemistry panel assessments will be performed at a local (site) laboratory from blood samples collected using institutional best practices before administration of study drug. Results and normal reference ranges will be entered into the eCRF.

**7.4.6.2. Hematology**

Hematology assessments, including complete blood count with differential, will be performed at a local (site) laboratory using institutional best practices before administration of study drug. Results and normal reference ranges will be entered into the eCRF.

**7.4.6.3. Pregnancy Testing**

A serum pregnancy test will be required for all women of childbearing potential during screening and at the EOT visit. Urine pregnancy tests will be conducted every 28 days. Urine pregnancy tests will be performed locally. If a urine pregnancy test is positive, the results should be confirmed with a serum pregnancy test.

If the serum pregnancy test is negative after a urine test was positive, the investigator will assess the potential benefit/risk to the subject and determine whether it is in the subject's best interest to resume study drug and continue participation in the study.

**7.4.6.4. Hepatitis Screening**

Subjects with active HBV or HCV infection that requires treatment or who are at risk for HBV reactivation are excluded from the study. At risk for HBV reactivation is defined as hepatitis B surface antigen positive or anti–hepatitis B core antibody positive. Prior test results obtained as part of standard of care before allo-HSCT confirming that a subject is immune and not at risk for reactivation (ie, hepatitis B surface antigen negative, surface antibody positive) may be used for purposes of eligibility, and tests do not need to be repeated. Subjects with prior positive serology results must have negative polymerase chain reaction results. Subjects whose immune status is unknown or uncertain must have results confirming immune status before enrollment.

**7.4.6.5. HIV Screening**

Subjects with an active HIV infection are excluded from the study. Prior HIV screening results obtained as standard of care for allo-HSCT confirming the subject is HIV-negative may be used for determining eligibility, and tests do not need to be repeated. Subjects whose HIV status is unknown must have results confirming negative status before enrollment.

## 7.5. Efficacy Assessments

Assessments will be performed at three stages: 1) prior-treatment screening to assess the inclusion and exclusion criteria (once from days -7 to -1 before medication); 2) treatment evaluation to determine the response and adjustment of medication maintenance (twice weekly for week 1, once weekly from week 2 to 6, once every 2 weeks from week 7 to 12, and once every month from week 13 to 24 after the start of medication n); 3) post-treatment evaluation for follow-up (every 2 months for 6 months).

### 7.5.1. GVHD Staging and Grading

Acute GVHD grading will be performed by the investigator on a weekly basis for the first 8 weeks after randomization, then every 28 days thereafter. GVHD staging and grading will also occur on Days 100, 180, and 365 and at the EOT visit.

On-treatment acute GVHD grading should be performed relative to the Day 1 assessment.

If subjects withdrew due to reasons other than GVHD progression, then GVHD staging and grading will be assessed at the safety follow-up visit and every 28 days thereafter during survival follow-up until progression of GVHD, start of new anti-GVHD therapy, or death.

Data regarding the quantification of acute GVHD symptoms (extent of skin rash, total bilirubin level, volume of diarrhea) should be reported using MAGIC guidelines ^[19]^; response will be assessed as per CIBMTR modifications to the IBMTR response index.

- Skin:
- Only areas involved with active erythema should be used for determination of body surface area staging based on the rule of nines.
- A portion of a body area segment may be used for the quantification.
- Desquamation or fluid-filled bullae should be reported if present, as these findings are the hallmark of Stage 4 skin GVHD.
- Liver:
- Liver GVHD staging is based solely on total (not conjugated/direct) serum bilirubin levels.
- Liver GVHD manifesting as transaminitis without concomitant elevation in serum bilirubin should be diagnosed when the presence of GVHD is confirmed by liver biopsy (where appropriate) and score it as Stage 0.
- If bilirubin levels were elevated before the diagnosis of GVHD in another target organ and do not increase further, liver GVHD should not be diagnosed in the absence of biopsy confirmation. However, if hyperbilirubinemia develops at the same time or after the onset of GVHD in another target organ, liver GVHD is presumed to be present in the absence of an identified alternative cause.
- Upper GI:
- Symptoms of concern for upper GI GVHD include anorexia, nausea, vomiting, and dyspepsia, and assessment depends on close attention to caloric intake and symptom reporting.
- An upper GI endoscopy should be performed whenever possible to confirm upper GI GVHD; however, the diagnosis may be made without biopsy confirmation.
- GVHD is typically not considered as a possible etiology when nausea lasts fewer than 3 days, or with fewer than 2 vomiting episodes per day for at least 2 days, or anorexia without weight loss.
- Lower GI:
- Staging of lower GI GVHD relies on accurate measurement of daily stool volumes and documentation of the presence of hematochezia or severe abdominal pain.
- In cases where stool volume cannot be closely measured, volume should be calculated based on average of 200 mL per episode multiplied by the number of episodes in a 24-hour period.
- At the time of GVHD onset, staging should be based on the highest daily volume during the 3 days before diagnosis (excluding volumes attributable to procedures such as bowel preps or endoscopy).
- After the initiation of treatment, lower GVHD staging should be based on the diarrhea volume using the following measurements (in the order of preference): 1) average of 3 consecutive days, 2) average of 2 consecutive days, or 3) the volume on day of assessment.
- Severe abdominal pain, ileus, and/or grossly bloody stool should be documented when present because Stage 4 lower GI GVHD is staged based on the presence of these symptoms and is independent of volume of diarrhea.

### 7.5.2. Chronic GVHD Assessment

Subjects will be assessed for signs and symptoms of cGVHD according to local institutional practice at screening, during the treatment phase, at the end of treatment, and during re-treatment (if applicable). Definitive and possible manifestations of cGVHD should be assessed as per NIH consensus guidelines for cGVHD^[32]^.

### 7.5.3. Graft Failure and Donor Chimerism

Monitoring of graft failure will be primarily based on the monitoring of blood counts with subsequent confirmation by chimerism studies, as clinically indicated. Donor chimerism after a HSCT involves identifying the genetic profiles of the recipient and of the donor and then evaluating the ratio of donor to recipient cells in the recipient’s blood, bone marrow, or other tissue. Chimerism testing using peripheral blood or bone marrow will be performed at the treating investigator’s discretion according to local institutional practice. In general. Genomic polymorphisms should be assessed via polymerase chain reaction analysis of short tandem repeat loci from isolated lymphocytes or myeloid cells. Fluorescence in situ hybridization analysis may also be used in cases with sex-mismatched transplants. If a subject experiences graft failure (ie, initial blood or marrow donor chimerism > 5% declining to < 5% on subsequent measurements), any action taken, including rapid taper of immunosuppression, administration of nonscheduled donor lymphocyte infusion, stem cell boost or other intervention(s), should be recorded on the appropriate eCRF.

### 7.5.4. Post-Transplant Lymphoproliferative Disorder Assessment

Staining for Epstein-Barr virus for PTLD testing will be performed according to local institutional practice at the treating investigator’s discretion.

### 7.5.5. Relapse/Recurrence of Underlying Hematologic Disease

Subjects will be followed for relapse or recurrence of their underlying hemotologic disease as per institutional standards during treatment and follow-up. Details on hematologic disease relapse will be recorded on the appropriate eCRF.

New malignancies should be reported as separate AEs.

## 7.6. Blood Sample Collection

Blood samples for GVHD biomarkers will be obtained at acute GVHD onset for all subjects. Then, the subjects will be randomized to receive one of the two treatments according to the risk of acute GVHD.

# 8. STATISTICS

## 8.1. Selection of Sample Size

The sample size is calculated according to the primary endpoint (ORR) of the study. Based on our published phase I study of 32 acute GVHD patients who received steroids as first-line therapy, an expected proportion of 55% for the patients treated with corticosteroids only was established ^[4]^. Based on our published data of patients with acute GVHD grade I–IV who received steroid-ruxolitinib (5 mg/day) as first-line therapy, the ORR was 82.05% ^[24]^. Patients with acute GVHD grade I were also included in the phase I analysis. Thus, an expected proportion of 75% for the patients treated with steroid-ruxolitinib (5mg/day) was established. This study is planned to detect a response difference between treatment arms at a two-sided significance level α=5% with a power of 1-β=80%. The sample size was estimated using PASS software based on the primary endpoint (ORR). The calculated sample size is shown in Figure 1 (N1=99, N2=99); allowing a withdrawal rate of 10%, 198 patients (99/group) will be required.

## 8.2. Level of Significance

The 1-sided significance level for the primary and key secondary endpoint analysis is 0.025. The 2-sided significance level for other analyses is 0.05. All CIs will be 95%.

## 8.3. Statistical Analyses

### 8.3.1. Primary Analyses

The baseline characteristics of patients will be reported in detail. The discrete variables are described by a median with IQR. The mean and standard deviation (SD) is used for quantitative variables. A logistic regression model is established as the primary study endpoint of GVHD treatment, as described above. In this model, the treatment grouping factor (ruxolitinib plus corticosteroids vs. corticosteroids alone), stratified variables of acute GVHD risk (high-risk vs. intermediate risk), and disease status before transplantation (complete remission vs. non- complete-remission) are included as covariates. The subgroup analysis is based on the significant effect of biomarker risk stratification on survival outcomes.

### 8.3.2. Secondary Analyses

The estimated rates with two-sided 95% confidence intervals (CIs) are used for secondary endpoints (proportion of CR patients and patients who stopped treatment). The ORR on day 28 is calculated with its 95% CI. Kaplan–Meier method is used to estimate the DOR, OS, and DFS, and the log-rank test is used to evaluate the difference between the groups ^[21]^. The cumulative incidence of NRM and relapse is estimated using a competing risk model and compared using the Fine and Gray test. The Cox proportional hazard regression model is used for multivariable regression analysis for OS and DFS. The multivariable regression analysis for NRM is performed using the Fine–Gray proportional hazard regression for competing events. Potential risk factors considered in the regression analysis include primary disease, cytogenetic risk, age and gender of the donor and recipient, graft source, treatment arm, acute GVHD risk, and disease status before transplantation. The cumulative incidence of recurrent acute GVHD is analyzed using the Fine and Gray test in a competing risk framework. The threshold for statistical significance is set at 0.05, and all tests are two-sided. All analyses are carried out using SPSS 22.0 software (IBM Corporation, Armonk, NY, USA) and R version 4.1.2 (www.cran.r-project.org). The treatment-induced differences in OS and DFS will be based on the Cox proportional hazard regression model. The reported treatment difference in NRM and relapse will be based on the multivariate analysis using Fine–Gray proportional hazard regression.

### 8.3.3. Safety Analyses

A TEAE is either an AE reported for the first time or worsening of a pre-existing condition after first dose of study drug. Analysis of AEs will be limited to TEAEs, but data listings will include all AEs, regardless of their timing to study drug administration. Severity of AEs will be based on NCI CTCAE v4.0.

## 8.4. Handling of missing data

All randomized participants will be included in the primary analysis of all outcomes. The proportion of missing values on the primary and secondary outcomes would be <10%. Thus, a secondary analysis should be considered using multiple imputations and present best-case/worst-case scenarios if ignoring the missing data is not plausible.

## 8.5. Analyses for the Data Monitoring Committee

All data from the trial would be maintained confidential by the hospital’s data monitoring committee (DMC); also, it would help in data storage and analysis. The DMC of Chinese PLA General Hospital is composed of staffs in the clinical research center who will inspect the trial dataset. DMC is independent from the sponsor. The charter of DMC can be obtained from the clinical research center if necessary.

# 9. ETHICAL APPROVAL

**Ethics Committee of PLA General Hospital**

**Approval Document of Clinical Research Project**

**No. S 2019-177-01**

| **Preview project** | **Project name** | Prospective, Randomized, Controlled Study of Novel Strategies for Treatment of Acute GVHD Based on Risk Stratification | | | |
| --- | --- | --- | --- | --- | --- |
|  | **Project Source** | International Cooperation Project□  National Scientific Research Project□  Military Scientific Research Project□  Enterprise Cooperation Project□  Beijing Scientific Research Project■  Hospital Scientific Research Project□  Researcher Spontaneous Project□  Other□ | | | |
|  | **Project No** | 7172200 | **Starting Time** | 2019.6 | |
|  | **Office** | Department of Hematology, the First Medical Center of PLA General Hospital | **Principal Investigator** | Daihong Liu | |
|  | **Job Title** | Chief Physician/Professor | **Contact number** | 13681207138 | |
|  | **Review Date** | 2019.7 | **Meeting Place** | NA | |
|  | **Review Approach** | □Conference Review■ Expedited review | | | |
| **Processing and Review of Submitted Documents** | Preliminary review documents:  Test scheme: Version number: 2019.5.18  Version Date: May 18, 2019  Informed consent form (including instructions to patients): version number: 2019.5.18 Version Date: May 18, 2019  Recruitment Advertisement (if any): Version number: Version Date:  Other information provided to the subject (if any): | | | |  |
| **Review Comments** | **1. This clinical study was agreed upon by this ethics committee.**  **Comments and suggestions: ■ No□Yes**  **2. Annual/regular follow-up review by the Ethics Committee on the implementation of the study: ■ Yes□No**  **The frequency of review is the date of approval of the study: □3 months□6 months■12 months.**  **3. The Ethics Committee has the right to change the frequency of annual/periodic follow-up reviews based on actual progress.**  **4. If the project is not started within one year from the date of approval, the approved document will automatically become invalid.** | | | |  |
| **Signature of the Chair Cor the authorized vice-chair/ EC member):**  **Ethics Committee(seal):**  **Year Mouth date** | | | | |  |

Address: No. 28, Fuxing Road, Haidian District, Beijing 100853 Contact: Jiang Cao Tel: 010-66937166

**Ethics Committee of PLA General Hospital**

**Decisions Letter for Scientific Research Project**

**No. S 2019-177-02**

**Approval No. of Ethics Committee**

| **Preview Information** | **Project name** | | Prospective, Randomized, Controlled Study of Novel Strategies for Treatment of Acute GVHD Based on Risk Stratification | | | | |
| --- | --- | --- | --- | --- | --- | --- | --- |
|  | **Project Source** | | Beijing Scientific Research Project | | | | |
|  | **Project Type** | | - | | | | |
|  | **Project No** | | 2019-177 | | **Starting and Ending Times** | **2019/05/27-** **2022/10/01** | |
|  | **Office** | | Department of Hematology | | **Principal Investigator** | **Daihong Liu** | |
|  | **Job Title** | | Professor | | **Contact number** | 13681171597 | |
| **Review Type** | **Amendment Review** | | | | | | |
| **Review Approach** | **Expedited Review** | | | | | | |
| **Date** | 2022/9/28 | **Meeting Place** | | - | | |  |
| Please find attached list of the documents for review and The other documents submitted this time (including version No. and version date) | | | | | | |  |
| The review result on the trial/research by the Ethics Committee is as follows:  Approval | | | | | | |  |
| **The details of the comments:**  About the research program:  No  About the informed consent:  No  About the recruitment Ads:  No8 | | | | | | |  |

Address: No. 28, Fuxing Road, Haidian District, Beijing 100853 Contact: Jiang Cao Tel: 010-66937166

1/4

| Others:  No |
| --- |
| The Approval Period of EC Decisions Letter(Approval):  If the trial/research is not initiated in 1 year, the trial/research needs to be reviewed again.  The approval period of EC approval certificate means that a period of time in which the trial/research is initiated the EC approval certificate is effective from the approval date. If the trial/research is not initiated in the approval period, the trial/research needs to be reviewed again. If the trial/research is initiated in the approval period, this approval certificate is effective. |
| Does it need to change the regular review frequency (applicable for tracking review)?  **■**No  **□**Yes,  The frequency of regular review revised:  **□**3 months  **□**6 months  **□**12 months  口others (specify): |
| **Signature of the Chair Cor the authorized vice-chair/ EC member):**  **Ethics Committee(seal):**  Year Mouth date |

Address: No. 28, Fuxing Road, Haidian District, Beijing 100853 Contact: Jiang Cao Tel: 010-66937166

2/4

| Note:   1. The "Approval" trial/research shall be implemented following the protocol approved by the Ethic Committee, and conforms to the principles of NMPA/GCP and Declaration of Helsinki. 2. During the research process, any revisions made to the documents related to the protocol and Informed Consent Form can't be implemented before obtaining the approval from the Ethics Committee. 3. The Serious Adverse Events or accidents affected the subject" safety or welfare occurred in this center shall be reported timely in writing to the Ethics Committee while reporting to NMPA, because the Ethics Committee has the right to make new decision on its evaluation. 4. The trial /research involving the export of human genetic resources or special examination should be approved by the related departments before the trial /research is initiated. 5. Please conduct the trial/research within the approval period, otherwise the approval certificate of ethical review is expired. 6. The trial/research whose the approval certificate of ethical review is expired should be reviewed again. |
| --- |
| Declaration:  The composition and process program of this Ethics Committee are eligible for <Good Clinical Practice>, <Declaration of Helsinki>, <Guideline for Ethical Review of Drug Clinical Trials>, <International Ethical Guidelines for Biomedical Research Involving Human Subjects>, <Regulations for ethical review of biomedical research involving human (National)> and relevant laws and regulations. |

Address: No. 28, Fuxing Road, Haidian District, Beijing 100853 Contact: Jiang Cao Tel: 010-66937166

3/4

**Attachment:**

**List of Documents Submitted for Ethics Review**

**No. S 2019-177-02**

1. Amendment Scheme Application Report
2. Informed Consent (Version number:V2 Version Date: 2022/05/23)
3. Research Scheme (Version number:V2 Version Date: 2022/05/23)
4. The List of Researchers at the Cooperation Center.

Address: No. 28, Fuxing Road, Haidian District, Beijing 100853 Contact: Jiang Cao Tel: 010-66937166

4/4

# 10. REFERENCES

[1] Malard F, Huang XJ, Sim J. Treatment and unmet needs in steroid-refractory acute graft-versus-host disease. Leukemia. 2020. 34(5): 1229-1240.

[2] Dou L, Hou C, Ma C, et al. Reduced risk of chronic GVHD by low-dose rATG in adult matched sibling donor peripheral blood stem cell transplantation for hematologic malignancies. Ann Hematol. 2020. 99(1): 167-179.

[3] Dou LP, Li HH, Wang L, et al. Efficacy and Safety of Unmanipulated Haploidentical Related Donor Allogeneic Peripheral Blood Stem Cell Transplantation in Patients with Relapsed/Refractory Acute Myeloid Leukemia. Chin Med J (Engl). 2018. 131(7): 790-798.

[4] Hou C, Dou L, Jia M, et al. Ruxolitinib Combined with Corticosteroids as First-Line Therapy for Acute Graft-versus-Host Disease in Haploidentical Peripheral Blood Stem Cell Transplantation Recipients. Transplant Cell Ther. 2021. 27(1): 75.e1-75.e10.

[5] Zeiser R, Blazar BR. Acute Graft-versus-Host Disease - Biologic Process, Prevention, and Therapy. N Engl J Med. 2017. 377(22): 2167-2179.

[6] Xhaard A, Launay M, Sicre de Fontbrune F, et al. A monocentric study of steroid-refractory acute graft-versus-host disease treatment with tacrolimus and mTOR inhibitor. Bone Marrow Transplant. 2020. 55(1): 86-92.

[7] Escamilla Gómez V, García-Gutiérrez V, López Corral L, et al. Ruxolitinib in refractory acute and chronic graft-versus-host disease: a multicenter survey study. Bone Marrow Transplant. 2020. 55(3): 641-648.

[8] de Kort EA, van Dorp S, Blijlevens N, van der Velden W. Corticosteroid replacement by ruxolitinib in patients with acute GVHD experiencing severe steroid-induced side effects. Bone Marrow Transplant. 2020. 55(1): 253-255.

[9] Baake T, Jörß K, Suennemann J, et al. The glucocorticoid receptor in recipient cells keeps cytokine secretion in acute graft-versus-host disease at bay. Oncotarget. 2018. 9(21): 15437-15450.

[10] Jagasia M, Zeiser R, Arbushites M, Delaite P, Gadbaw B, Bubnoff NV. Ruxolitinib for the treatment of patients with steroid-refractory GVHD: an introduction to the REACH  trials. Immunotherapy. 2018. 10(5): 391-402.

[11] Zeiser R. Advances in understanding the pathogenesis of graft-versus-host disease. Br J Haematol. 2019. 187(5): 563-572.

[12] Li H, Kaiser TK, Borschiwer M, et al. Glucocorticoid resistance of allogeneic T cells alters the gene expression profile in the inflamed small intestine of mice suffering from acute graft-versus-host disease. J Steroid Biochem Mol Biol. 2019. 195: 105485.

[13] Schroeder MA, Choi J, Staser K, DiPersio JF. The Role of Janus Kinase Signaling in Graft-Versus-Host Disease and Graft Versus Leukemia. Biol Blood Marrow Transplant. 2018. 24(6): 1125-1134.

[14] Srinagesh HK, Ferrara J. MAGIC biomarkers of acute graft-versus-host disease: Biology and clinical application. Best Pract Res Clin Haematol. 2019. 32(4): 101111.

[15] Zeiser R, Socié G. The development of ruxolitinib for glucocorticoid-refractory acute graft-versus-host disease. Blood Adv. 2020. 4(15): 3789-3794.

[16] Schoemans HM, Lee SJ, Ferrara JL, et al. EBMT-NIH-CIBMTR Task Force position statement on standardized terminology & guidance for graft-versus-host disease assessment. Bone Marrow Transplant. 2018. 53(11): 1401-1415.

[17] Srinagesh HK, Levine JE, Ferrara J. Biomarkers in acute graft-versus-host disease: new insights. Ther Adv Hematol. 2019. 10: 2040620719891358.

[18] Levine JE, Braun TM, Harris AC, et al. A prognostic score for acute graft-versus-host disease based on biomarkers: a multicentre study. Lancet Haematol. 2015. 2(1): e21-9.

[19] Major-Monfried H, Renteria AS, Pawarode A, et al. MAGIC biomarkers predict long-term outcomes for steroid-resistant acute GVHD. Blood. 2018. 131(25): 2846-2855.

[20] Jiménez-Jorge S, Labrador-Herrera G, Rosso-Fernández CM, et al. Assessing the impact on intestinal microbiome and clinical outcomes of antibiotherapy optimisation strategies in haematopoietic stem cell transplant recipients: study protocol for the prospective multicentre OptimBioma study. BMJ Open. 2020. 10(7): e034570.

[21] Jagasia M, Perales MA, Schroeder MA, et al. Ruxolitinib for the treatment of steroid-refractory acute GVHD (REACH1): a multicenter, open-label phase 2 trial. Blood. 2020. 135(20): 1739-1749.

[22] Zeiser R, von Bubnoff N, Butler J, et al. Ruxolitinib for Glucocorticoid-Refractory Acute Graft-versus-Host Disease. N Engl J Med. 2020. 382(19): 1800-1810.

[23] Delgado-Martin C, Meyer LK, Huang BJ, et al. JAK/STAT pathway inhibition overcomes IL7-induced glucocorticoid resistance in a subset of human T-cell acute lymphoblastic leukemias. Leukemia. 2017. 31(12): 2568-2576.

[24] Yang J, Peng B, Wang L, et al. Elevated REG3α predicts refractory acute GVHD in patients who received steroids-ruxolitinib as first-line therapy. Ann Hematol. 2022. 101(3): 621-630.

[25] von Bubnoff N, Ihorst G, Grishina O, et al. Ruxolitinib in GvHD (RIG) study: a multicenter, randomized phase 2 trial to determine the response rate of Ruxolitinib and best available treatment (BAT) versus BAT in steroid-refractory acute graft-versus-host disease (acute GVHD) (NCT02396628). BMC Cancer. 2018. 18(1): 1132.

[26] MacMillan ML, DeFor TE, Weisdorf DJ. The best endpoint for acute GVHD treatment trials. Blood. 2010. 115(26): 5412-7.

[27] Przepiorka D, Luo L, Subramaniam S, et al. FDA Approval Summary: Ruxolitinib for Treatment of Steroid-Refractory Acute Graft-Versus-Host Disease. Oncologist. 2020. 25(2): e328-e334.

[28] Paczesny S, Krijanovski OI, Braun TM, et al. A biomarker panel for acute graft-versus-host disease. Blood. 2009. 113(2): 273-8.

[29] Hartwell MJ, Özbek U, Holler E, et al. An early-biomarker algorithm predicts lethal graft-versus-host disease and survival. JCI Insight. 2018. 3(16).

[30] Kekre N, Antin JH. Emerging drugs for graft-versus-host disease. Expert Opin Emerg Drugs. 2016. 21(2): 209-18.

[31] Toubai T, Magenau J. Immunopathology and biology-based treatment of steroid-refractory graft-versus-host disease. Blood. 2020. 136(4): 429-440.

[32] Jagasia MH, Greinix HT, Arora M, et al. National Institutes of Health Consensus Development Project on Criteria for Clinical Trials in Chronic Graft-versus-Host Disease: I. The 2014 Diagnosis and Staging Working Group report. Biol Blood Marrow Transplant. 2015. 21(3): 389-401.e1.

# APPENDIX A. Table of Assessments

## Table 1: Schedule of Assessments

| Visist Day  Item | Sceening | D1 | D3 | D7 | D14 | D21 | D28 | D35 | D42 | D49 | D56 | D100 | D180 | +3m | +4.5m | +6m | +9m | +12m | +18m | +24m | +36m |
| --- | --- | --- | --- | --- | --- | --- | --- | --- | --- | --- | --- | --- | --- | --- | --- | --- | --- | --- | --- | --- | --- |
| Informed consent |  |  |  |  |  |  |  |  |  |  |  |  |  |  |  |  |  |  |  |  |  |
| I/E criteria |  |  |  |  |  |  |  |  |  |  |  |  |  |  |  |  |  |  |  |  |  |
| Contact IVRS |  |  |  |  |  |  |  |  |  |  |  |  |  |  |  |  |  |  |  |  |  |
| Demogragpy/disease history |  |  |  |  |  |  |  |  |  |  |  |  |  |  |  |  |  |  |  |  |  |
| Prior/concomitant medications |  |  |  |  |  |  |  |  |  |  |  |  |  |  |  |  |  |  |  |  |  |
| Supportive care medications |  |  |  |  |  |  |  |  |  |  |  |  |  |  |  |  |  |  |  |  |  |
| Physical examination |  |  |  |  |  |  |  |  |  |  |  |  |  |  |  |  |  |  |  |  |  |
| Vital signs |  |  |  |  |  |  |  |  |  |  |  |  |  |  |  |  |  |  |  |  |  |
| ECOG PS |  |  |  |  |  |  |  |  |  |  |  |  |  |  |  |  |  |  |  |  |  |
| acute GVHD grading |  |  |  |  |  |  |  |  |  |  |  |  |  |  |  |  |  |  |  |  |  |
| acute GVHD organ |  |  |  |  |  |  |  |  |  |  |  |  |  |  |  |  |  |  |  |  |  |
| acute GVHD response |  |  |  |  |  |  |  |  |  |  |  |  |  |  |  |  |  |  |  |  |  |
| cGVHD assessment |  |  |  |  |  |  |  |  |  |  |  |  |  |  |  |  |  |  |  |  |  |
| Chimerism/graft failure  assessment |  |  |  |  |  |  |  |  |  |  |  |  |  |  |  |  |  |  |  |  |  |
| EBV infection assessment |  |  |  |  |  |  |  |  |  |  |  |  |  |  |  |  |  |  |  |  |  |
| CMV infection assessment |  |  |  |  |  |  |  |  |  |  |  |  |  |  |  |  |  |  |  |  |  |
| Underlying disease relapse assessment |  |  |  |  |  |  |  |  |  |  |  |  |  |  |  |  |  |  |  |  |  |
| Ruxolitinib dose monitoring |  |  |  |  |  |  |  |  |  |  |  |  |  |  |  |  |  |  |  |  |  |
| Steroid dose monitoring |  |  |  |  |  |  |  |  |  |  |  |  |  |  |  |  |  |  |  |  |  |
| New acute GVHD therapies |  |  |  |  |  |  |  |  |  |  |  |  |  |  |  |  |  |  |  |  |  |
| Survival follow-up |  |  |  |  |  |  |  |  |  |  |  |  |  |  |  |  |  |  |  |  |  |

# APPENDIX B. PROTOCOL AMENDMENT SUMMARY OF CHANGES

## Amendment (Version) 2 (23 May 2022)

**Overall Rationale for the Amendment:**

The primary purpose of this amendment is to clarify the sample size and procedural requirements for study subjects to enter the re-treatment phase. Additional clarification on study procedures has been included as well.

**1. Synopsis; Section 4.5.1 Planned Number of Subjects; Section 8.1, Selection of Sample Size**

**Description of change:** Modified the sample size based on our published phase I study of 32 acute GVHD patients who received steroids as first-line therapy, an expected proportion of 55% for the patients treated with corticosteroids only was established. Based on our published data of patients with acute GVHD grade I–IV who received steroid-ruxolitinib (5 mg/day) as first-line therapy, the ORR was 82.05%.

Thus, this study will enroll approximately 99 subjects with clinically or pathologically confirmed Grade II to IV acute GVHD per treatment cohort, for a total of 198 subjects at approximately 7 study sites.

**Rationale for change:** To include less subjects in this clinical trial with sufficient effect at the same time.

**2. Synopsis; Section2. STUDY OBJECTIVES AND ENDPOINTS**

**Description of change:** Clarified the secondary endpoints.

**Rationale for change:** Clarification.
